# An attempt to reproduce a previous meta-analysis and a new analysis regarding the impact of directly observed therapy on tuberculosis treatment outcomes

**DOI:** 10.1371/journal.pone.0217219

**Published:** 2019-05-23

**Authors:** Brian McKay, Maria Castellanos, Mark Ebell, Christopher C. Whalen, Andreas Handel

**Affiliations:** Department of Epidemiology and Biostatistics, The University of Georgia, Athens, Georgia, United States of America; University Magna Graecia of Catanzaro, ITALY

## Abstract

Directly observed therapy (DOT) is almost universally used for the treatment of TB. Several meta-analyses using different methods have assessed the effectiveness of DOT compared to self-administered therapy (SAT). The results of these meta-analyses often conflict with some concluding DOT is superior and others that there is little or no difference. Meta-analyses can guide policymaking, but such analyses must be reliable. To assess the validity of a previous meta-analysis, we tried to reproduce it. We encountered problems with the previous analysis that did not allow for a meaningful reproduction. We describe the issues we encountered here. We then performed a new meta-analysis comparing the treatment outcomes of adults given treatment with SAT versus DOT. Outcomes in the new analysis are loss to follow-up, treatment failure, cure, treatment completed, and all-cause mortality. All data, documentation, and code used to generate our results is provided. Our new analysis included four randomized and three observational studies with 1603 and 1626 individuals respectively. The pooled relative risks (RR) are as follows: Lost to follow-up (RR = 1.2, 95% CI 0.9, 1.7), Treatment Failure (RR = 1.1, 95% CI 0.6, 2), Cure (RR = 0.9, 95% CI 0.8, 1.1), Treatment Completion (RR = 1, 95% CI 0.9, 1.1), Mortality (RR = 0.9, 95% CI 0.6, 1.3). Based on data from our new meta-analysis, the magnitude of the difference between DOT and SAT for all reported outcomes is small, and none of the differences are statistically significant.

## Introduction

Directly observed therapy (DOT) for the treatment of tuberculosis (TB) is a component of the World Health Organization’s (WHO) recommended DOTS program. While the DOTS program has been successful in reducing tuberculosis incidence, the average annual decrease is far short of the needed reduction to meet the goals of the WHO End TB strategy [1]. DOT requires patients with TB to be observed by another individual while taking their medicine. Evidence to support DOT’s effect on cure rates [2,3], adherence [2,3], or any other measure of treatment success is not very compelling [4,5]. Leading to an intense debate about the use and effectiveness of DOT [6–9].

A meta-analysis of data from six randomized controlled trials (RCT) suggested that DOT did not increase the cure or treatment completion significantly [3]. We believe that due to the small number of RCT and the high likelihood that there will not be any new ones addressing this issue, it is necessary also to consider the evidence from observational studies. Tian and others reported the findings from eight RCTs and fifteen observational studies [10]. They found DOT to be more successful than SAT when the analysis was restricted to observational studies, but no difference was observed among RCT. Their analysis included retrospective cohort and cross-sectional studies, which are generally considered to provide lower quality evidence. To ensure the best quality evidence, we believe only prospective studies should be considered [11].

One previous meta-analysis included both RCT and prospective cohort studies (PCS) [12]. This study by Pasipanodya and Gumbo (which will be referred to as PG), included five RCT and five PCS. The inclusion of prospective studies helps provide valuable information without the high risk of bias associated with retrospective studies. However, PG’s study was criticized due to methodological concerns [13–15]. Thus, calls to gather more evidence to define the effectiveness of DOT versus SAT are still being made [8].

Since the question of DOT vs. SAT is a significant public health issue, we deemed it valuable to try and reproduce the results found by PG, taking into account and addressing previously raised criticisms as needed. In doing so, our investigation found other inconsistencies in PG beyond those already raised [13–15]. This prevented us from reproducing the results presented in PG. We instead performed an entirely new analysis that is reproducible and based on what we consider to be the most appropriate data and analysis methodology. Our analysis confirms results for RCT studies that there is little evidence for the superiority of DOT versus SAT in regards to any of the WHO-defined treatment outcomes.

## Methods

### Standards

We followed the Preferred Reporting Items for Systematic Review and Meta-Analysis (PRISMA) guidelines [16].

### Search strategy

We performed a systematic search of the literature from January 1st, 1960 to July 7th, 2016. The search included Pub Med and gray literature such as The Cochrane library. The studies found were reviewed and assessed by two researchers independently for inclusion (BM and MC), and consensus resolved any conflict. We attempted to contact authors if the published data were not complete or were reported in a manner that results in the desired population were not available. Further details can be found in the supplementary material (SM).

### Study selection criteria

Included studies met the following criteria: patients were diagnosed by microscopic examination of sputum smear or culture, assigned to either DOT or SAT, evaluated for treatment failure during the treatment, and were treated with short-course chemotherapy regimen including isoniazid, rifampin, and pyrazinamide. Study designs were limited to RCT and PCS. Studies were excluded based on the following criteria: study population includes children (< 15 years), retrospective design, included specialized re-treatment regimens or biased assignment of patients receiving re-treatment. Studies were not excluded based on the language unless reasonable attempts to have them translated failed.

### Exposure definitions

We used DOT definitions created by the WHO, which defines DOT as “an element of the DOTS program and refers to the direct observation of patients swallowing their medication” [17]. The observers in some studies are family members, community members or lay medical personnel. The training level of these individuals is not always clear, but studies have shown the impact of training level on outcomes is negligible [2,18–21]. When a patient’s treatment involves little or no supervision, it is considered SAT.

### Outcomes

Outcomes are defined by the WHO [17]. Included in the final analysis are lost to follow-up (LTFU), treatment failure, treatment completion, death, and cure. Lost to follow-up is used in place of the term “default” and is defined as an individual that does not complete treatment.

### Data abstraction and quality assessment of included studies

Data were abstracted independently by two researchers (BM and MC), and any disagreements were settled by consensus. We contacted the authors of studies that include children, retreatment, or extrapulmonary TB in an attempt to get the data excluding these individuals. Similarly, the authors of papers that only report some of the treatment outcomes or do not report the data in a usable format were also contacted.

To assess study quality, we used the “Cochrane Risk of Bias Tool for Cohort Studies” (CRBCS) to evaluate bias and quality of the PCS [22], and the Jadad score for RCT [23]. Two investigators (BM and MC) assessed study quality independently, and any disagreements were resolved by consensus. We compared the results of our data re-abstraction, study selection, and study quality to those obtained in PG. A detailed examination of differences in study inclusion between PG and our analysis is included in the SM.

### Data analysis

All analyses were done in R [24] using the “metafor” package [25]. Relative risk (RR) was used as the primary outcome. RR is preferred over risk difference (RD) because RR accounts for the baseline risk and provides a more consistent summary of effects [26].

Relative risk was calculated as SAT risk over DOT risk. A RR less than 1 for a positive outcome or a RR greater than 1 for an adverse outcome indicates that DOT is favored.

Since some of the studies had no events for outcomes, we used a continuity correction of adding .5 to all the cells in any 2x2 table with a single 0. We did not exclude studies with zero outcomes from the RR analysis [27]. PG reported effect size using the DerSimonian Laird (DL) method. In a meta-analysis of only a few studies, with each study having a diverse sample and effect size, the DL method can lead to biased estimates of overall effect and underestimate the amount of heterogeneity [28–31]. We believe the use of restricted maximum likely hood is more appropriate and used it for all the random effects models [28,32].

To decide on the statistical model to use, PG used fixed effects if *I*^2^ < 30% and random effects if *I*^2^ > 30%. While this approach is common, we contend that it is not entirely appropriate. Instead, the model choice should be determined based on the assumptions about the underlying biology and distribution of the included studies [33]. Since the studies will likely be from different settings with different populations, there is no reason to assume *a priori* that effect sizes should be the same. Therefore, a random effects model is more appropriate irrespective of the amount of heterogeneity, and it was used for all effect size calculations [33].

PG performed a meta-regression to investigate and explain the observed heterogeneity between studies. We decided that due to the small sample size it was not appropriate. Instead, we examined the effects of study design via subgroup analysis. As done by PG, publication bias is assessed using visual inspection and the Egger test of funnel plot symmetry [34]. Since the overall sample of studies is small, these tests may lack sufficient power. We therefore also included Orwin’s method of “N Fail Safe” and the “Trim and Fill” methods to provide additional information when judging publication bias [35].

We also performed a systematic sensitivity analysis to identify studies that may be outliers and studies with a significant level of influence on the estimated effect size.

## Results

### Reproducibility of PG

#### Study selection

Our set of studies included four RCTs and two PCSs. These studies differed from studies reported in PG: one RCT [36] and 4 PCS [37–40] included in PG should not have been included by PG based on their stated exclusion and inclusion criteria. We found two additional observational studies that met the inclusion criteria [41,42] which were not included in PG. With the information provided by PG, it is not possible to tell whether the studies were not found by PG or if they were included in the search results but not included in the analysis. Further details are provided in the SM.

#### Quality assessment of studies

We re-evaluated the quality of all the included studies from PG with the Jadad score for comparison. Assessing the quality of the RCT, we found a low risk of bias and overall high quality for three RCT which agreed with PG. There was disagreement for one RCT that we found to have a high risk of bias [36]. Assessing the quality of PCS with the Jadad score, we did not agree with PG that there was a low risk of bias. Using the Jadad score all of the PCS are given a score of 1, but when re-evaluated with the CRBCS all but one of the studies was shown to be of good quality. Further details are provided in the SM.

#### Treatment outcomes

**Fig 1:**
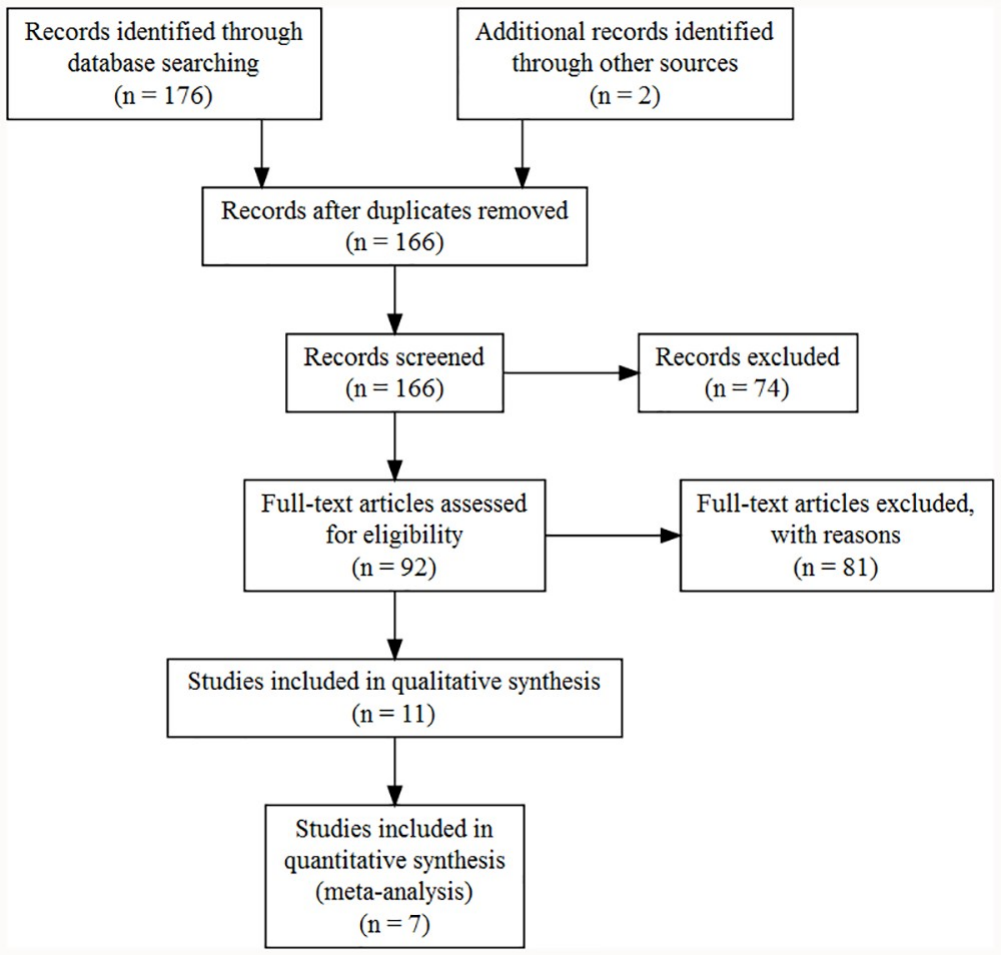
PRISMA diagram. Results of systematic literature review.

We were unable to reproduce PG’s results for lost to follow-up (Fig 1 and Table 2 in [12]), even when we tried to use the same underlying studies, data, and methods. We provide detailed information on our inability to reproduce those results (Figures P-R and Tables Z-AA in SM). PG analyzed the outcomes of relapse and acquired drug resistance. Since none of the studies which reported these outcomes meet the inclusion criteria (either ours or those in PG), and since these studies were not designed to assess the differences between DOT and SAT for these outcomes [13], we decided that those analyses were not meaningful and did not try to perform them.

### New meta-analysis of DOT vs. SAT

#### Study Selection and Characteristics

Our systematic review generated 166 unique results, 11 [39–49] studies compared DOT and SAT treatment outcomes in a prospective manner (Fig 1). We contacted the authors of 4 papers in an attempt to get the data on the population of interest; specifically, data on adults only [39,40,42,48] and data stratified by new or re-treatment patients [42] for certain outcomes. We received responses from authors of 2 studies, but neither was able to provide the data. We received no response from the corresponding authors of the other papers. In total 7 [41–44,46,47,49] studies met the inclusion criteria for at least one of the outcomes of interest.

The total number of individuals across all included studies is 3229. This number is less than the 13751 individuals reported in PG due to erroneous inclusion of several additional studies, as described above. Of those 3229 individuals, 1603 were randomized to interventions, 914to DOT, and 689 to SAT. The remaining 1626 patients came from PCS and were not randomly assigned to a treatment group, 998 received DOT, and 628 received SAT. A spreadsheet which provides detailed information on the systematic search and data abstraction is provided in the SM.

#### Quality assessment of included studies

Assessing the quality of the RCT [43,44,47,49], we found a low risk of bias and overall high quality. Assessing the quality of the PCS, we found one study to be of high quality with a low risk of bias [46]. The other two studies were found to be at an increased risk of bias [41,42]. A spreadsheet detailing the quality assessment of all the studies is included in the SM.

#### Lost to follow-up

**Fig 2:**
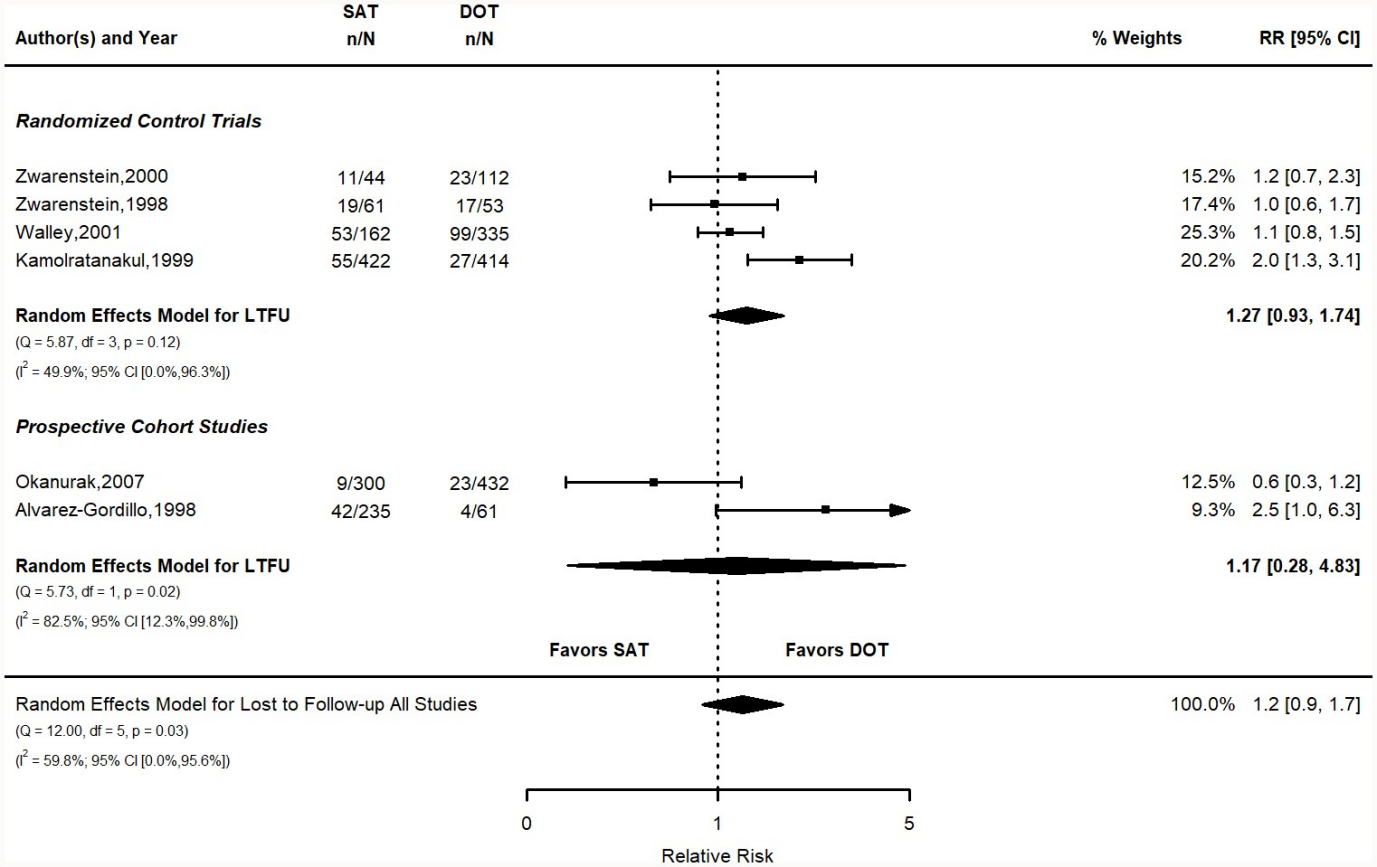
LTFU relative risk stratified by study design. Q = Cochrane’s Q; df = Degrees of Freedom; p = P-value associated with Q; I^2^ = Proportion of variation due to heterogeneity and corresponding 95% confidence interval.

The pooled risk of LTFU in the SAT group was 19.7% (95% CI 10.2, 29.1) vs. 16.1% (95% CI 6.5, 25.7) for the DOT group (Fig 2). The calculated risks for individual studies are shown in the supplementary materials (Table A in S1 Text). The pooled RR was 1.2 (95% CI 0.9, 1.7). The pooled RD (RD = SAT—DOT) was 3.7% (95% CI -1.2, 8.7). Figure for the RD is in the SM (Figure A in S1 Text). Both measures indicate that DOT is favored in reducing the risk of LTFU, but the results are not statistically significant. This result disagrees with the significant difference favoring DOT reported in PG [12].

#### Treatment failure

**Fig 3:**
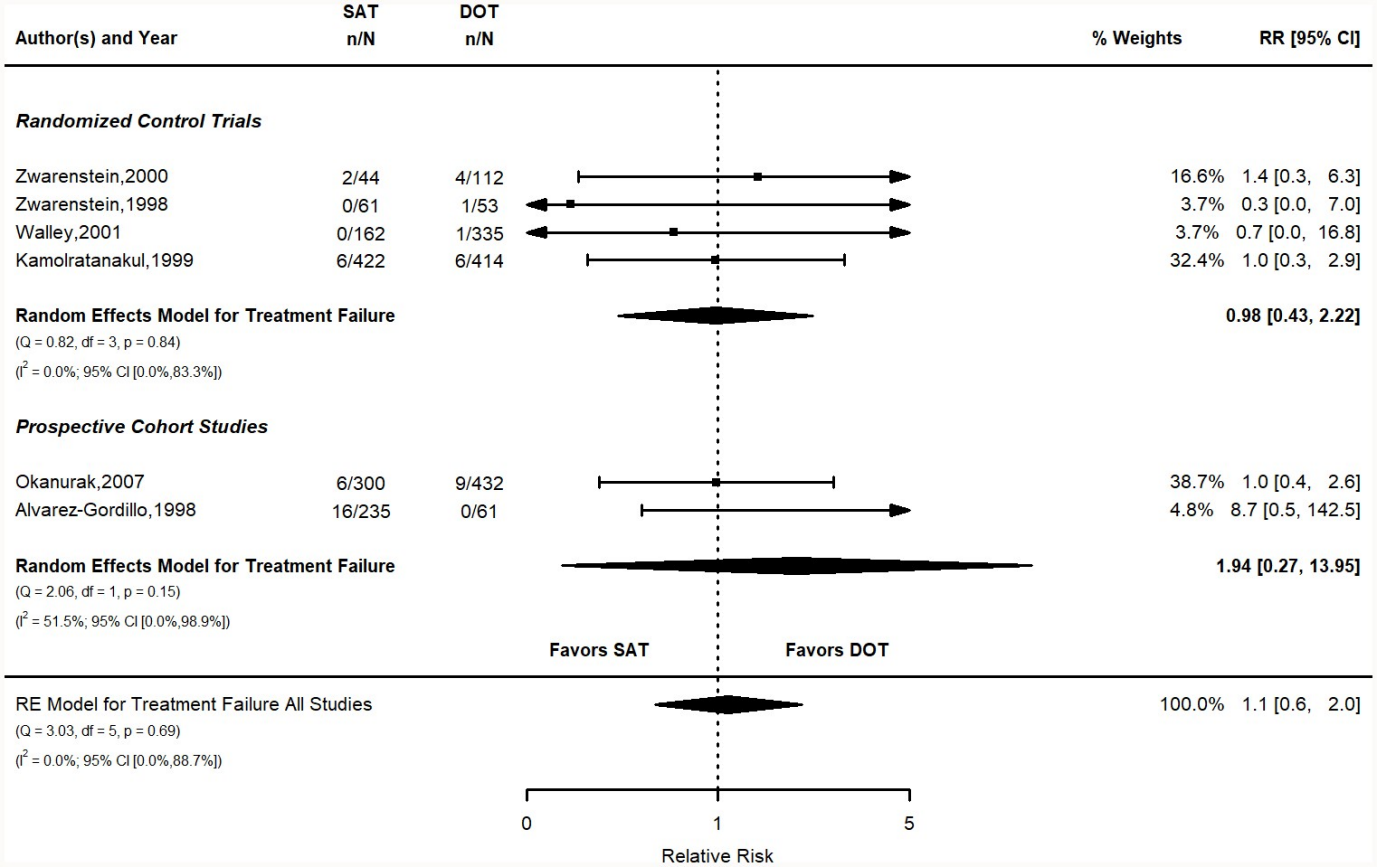
Treatment failure relative risk stratified by study design. Q = Cochrane’s Q; df = Degrees of Freedom; p = P-value associated with Q; I^2^ = Proportion of variation due to heterogeneity and corresponding 95% confidence interval.

The pooled risk of treatment failure in the SAT group was 2.1% (95% CI 0.3, 3.8) and 1.3 (95% CI 0.4, 2.1) for the DOT group (Fig 3). The calculated risks for individual studies are shown in the supplementary materials (Table B in S1 Text). The pooled RR was 1.1 (95% CI 0.6, 2). The pooled RD was 0.5% (95% CI -1, 1.9). Figure for the RD is in the SM (Figure B in S1 Text). Both measures indicate that DOT is slightly favored over SAT, but the results are not statistically significant.

#### Completion of treatment

**Fig 4:**
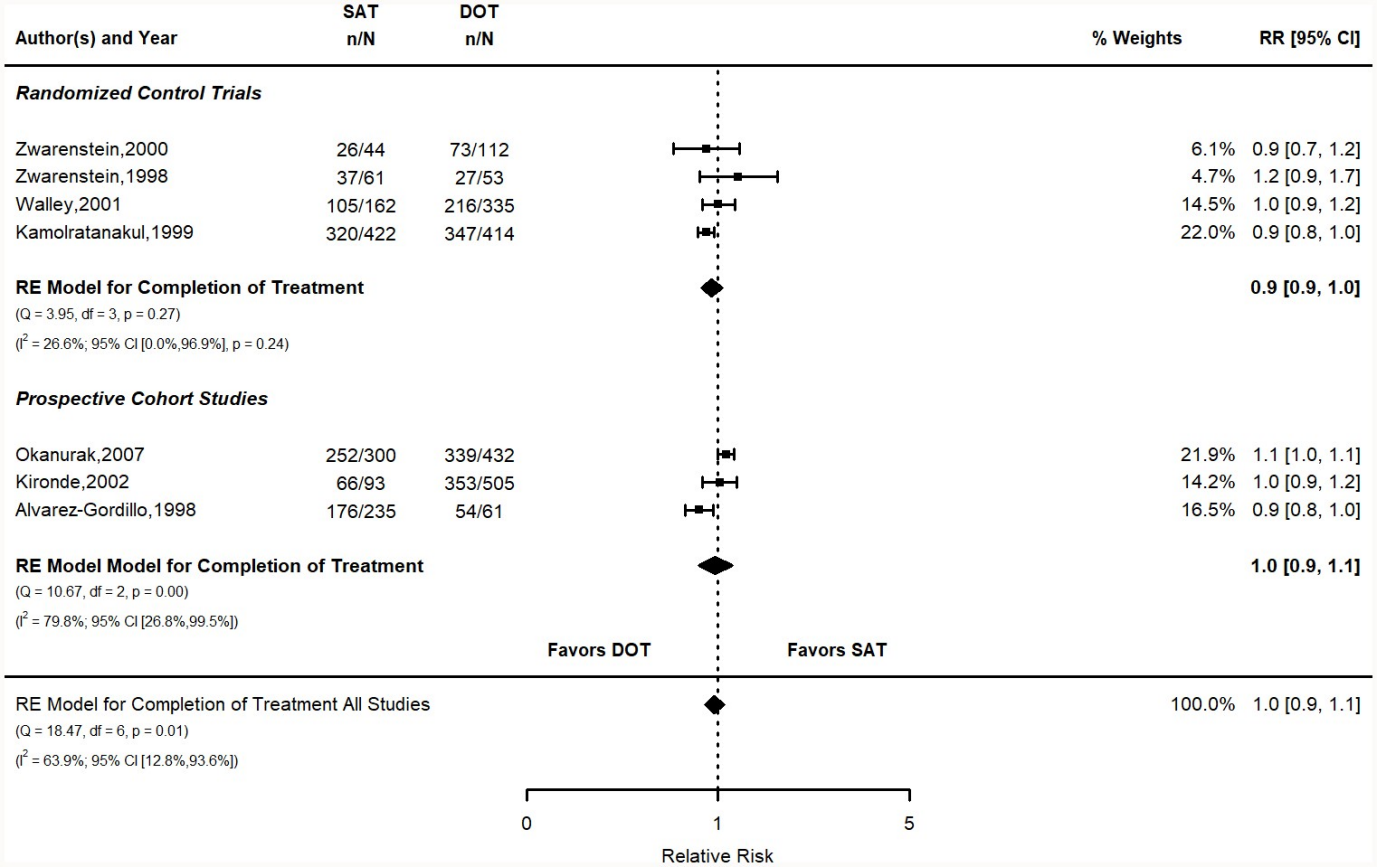
Completion of treatment relative risk stratified by study design. Q = Cochrane’s Q; df = Degrees of Freedom; p = P-value associated with Q; I^2^ = Proportion of variation due to heterogeneity and corresponding 95% confidence interval.

The pooled risk of completing treatment in the SAT group was 71.5% (95% CI 65, 78) vs. 72.3% (95% CI 63.4, 81.2) for the DOT group (Fig 4). The calculated risks for individual studies are shown in the supplementary materials (Table C in S1 Text). The pooled RR was 1 (95% CI 0.9, 1.1). The pooled RD was -2.1% (95% CI -8, 3.9). Figure for the RD is in the SM (Figure C in S1 Text). Both measures show no evidence of any difference between DOT and SAT.

#### Death

**Fig 5:**
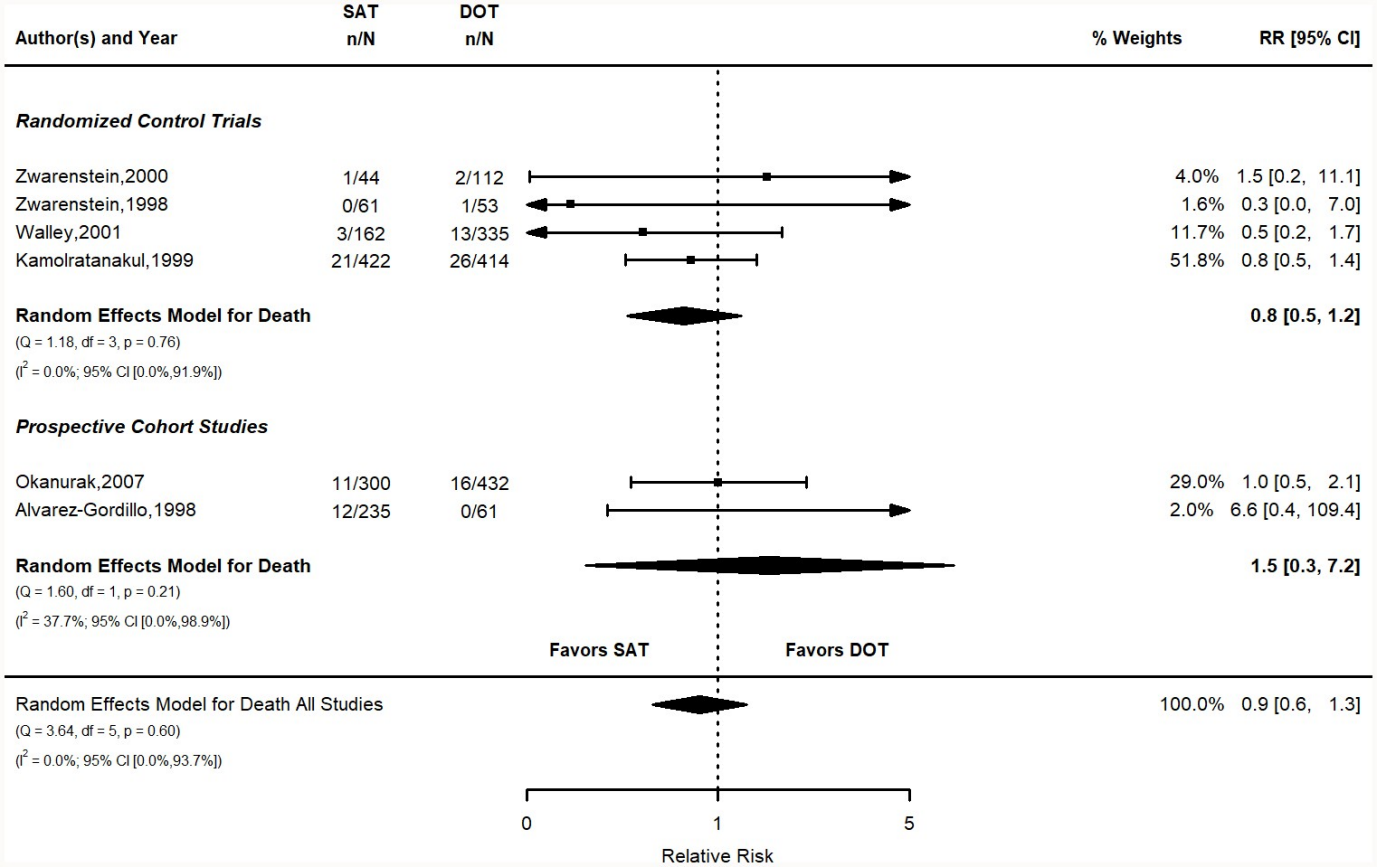
Death relative risk stratified by study design. Q = Cochrane’s Q; df = Degrees of Freedom; p = P-value associated with Q; I^2^ = Proportion of variation due to heterogeneity and corresponding 95% confidence interval.

The pooled risk of death in the SAT group was 3.1% (95% CI 1.6, 4.6) vs. 3.2% (95% CI 1.6, 4.7) for the DOT group (Fig 5). The calculated risks for individual studies are shown in the supplementary materials (Table D in S1 Text). The pooled RR was 0.9 (95% CI 0.6, 1.3). The pooled RD was -0.1% (95% CI -2.1, 1.9). Figure for the RD is in the SM (Figure D in S1 Text). The overall RR indicates that SAT is slightly favored over DOT, but the results are not statistically significant or consistent between study design type.

#### Cure

**Fig 6:**
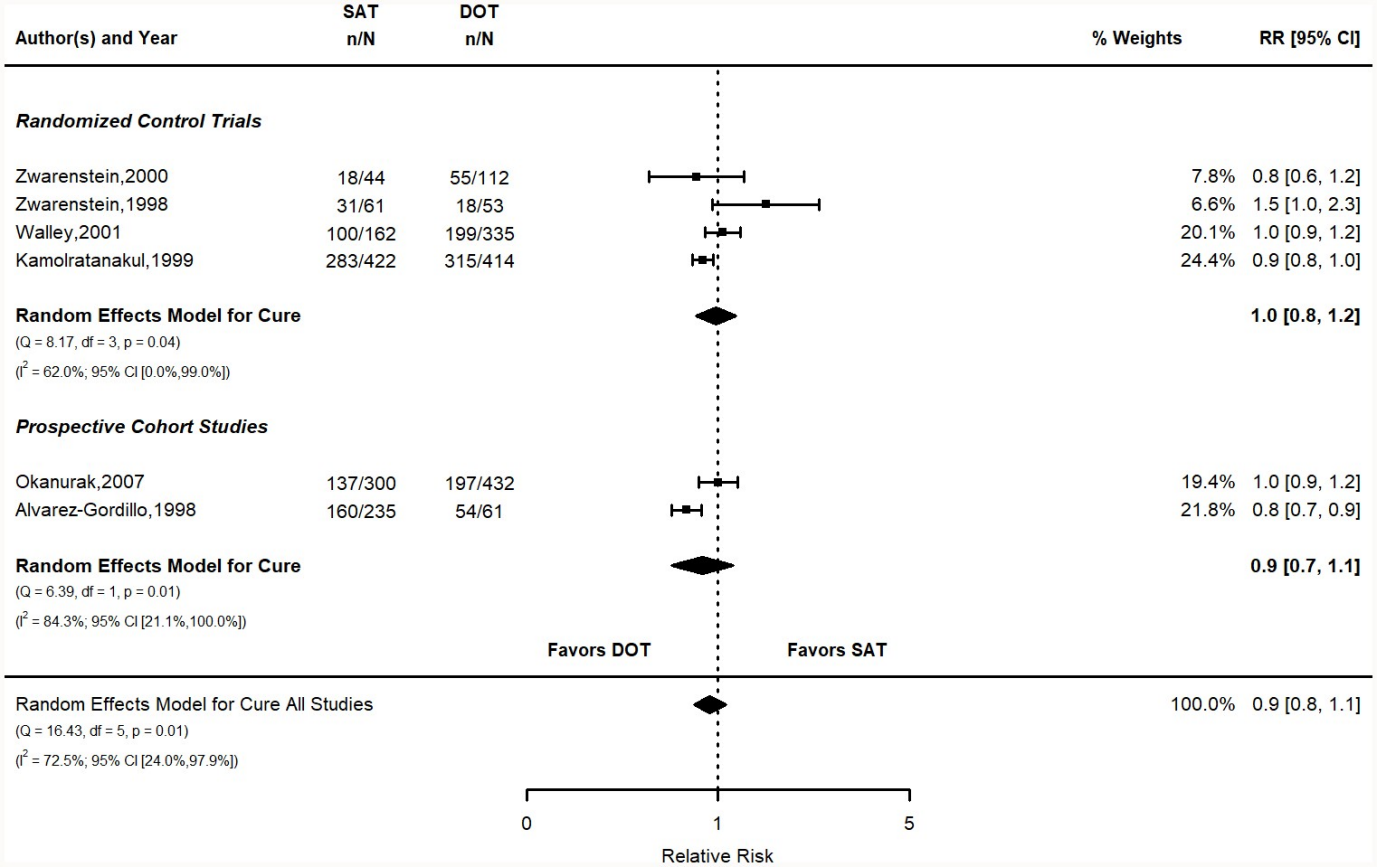
Cure relative risk stratified by study design. Q = Cochrane’s Q; df = Degrees of Freedom; p = P-value associated with Q; I^2^ = Proportion of variation due to heterogeneity and corresponding 95% confidence interval.

The pooled risk of cure in the SAT group was 56.7% (95% CI 47.6, 65.8) vs. 59.1% (95% CI 43, 75.1) for the DOT group (Fig 6). The calculated risks for individual studies are shown in the supplementary materials (Table E in S1 Text). The pooled RR was 0.9 (95% CI 0.8, 1.1). The pooled RD was -4% (95% CI -13.1, 5.1). Figure for the RD is in the SM (Figure E in S1 Text). Both measures indicate that DOT is slightly favored over SAT, but the results are not statistically significant.

#### Sensitivity and influence

Lost to follow-up was the only outcome that was sensitive to the absence of a single study [46]. When the study is removed, the point estimated risk difference becomes statistically significant, and the estimated heterogeneity goes to zero. Detailed results of the sensitivity and influence analysis for each outcome are included in the SM (Table F-Y in S1 Text).

#### Publication bias

None of the statistical tests or visual inspection of the funnel plots indicated the presence of publication bias for any of the included outcomes. It is important to note that the power to detect any publication bias is small with so few studies. Detailed results are shown in the SM (Figure F-O in S1 Text).

## Discussion

History has demonstrated that TB control is required to protect public health [50], but the DOT component of TB control has been more controversial. There are a number of meta-analyses that have been published, and the methodology used for the systematic search of the literature appears to impact the results. Specifically, the types of study designs included seems to determine if the findings are statistically significant or not. Studies that include retrospective studies favor DOT and show a statistically significant difference when comparing DOT and SAT [10,51,52].

Additionally, the definition for DOT and SAT groups can vary significantly within and between meta-analyses. This leads to differences in the studies included as well as how study groups are divided. These issues confirm the need for transparent and complete reporting of the methods used to generate the results for a meta-analysis. Recent Cochrane meta-analyses addressed the effectiveness of DOT, but only included RCT and concluded that there is little evidence in favor of DOT [2,3]. Due to the small number of RCT and the high likelihood that there will not be any new ones addressing this issue, it is useful to include data from PCS to provide a more comprehensive assessment of treatment outcomes for DOT and SAT. While the inclusion of PCS is controversial, it provides additional information and is useful when only a few RCT are available.

A previous meta-analysis by PG included both RCT and PCS. However, as described here, their analysis had methodologic issues rendering their results unreliable [13–15]. Our new meta-analysis corrects problems found in PG and expands the scope of the Cochrane review by including not only RCT but also PCS. We have attempted to make our study easy to reproduce. To that end, all the data and code required to reproduce all our results, as well as, documentation of the entire systematic review process, are provided in the SM. In contrast to PG, we did not find a statistically significant difference between DOT and SAT as regards to LTFU. Similar to past Cochrane reviews[2,3] and PG, we found no statistically significant difference between DOT and SAT for treatment failure. Our findings are in agreement with meta-analyses including only RCT [2,3]. Despite the inclusion of both RCT and PCS, the amount of data available for analysis is still limited, which constrains the power of the results and leads to at times wide confidence intervals. Attempts to obtain additional data from published studies were unsuccessful. The inclusion of these data could change the results of our analysis.

We did not address the effects of DOT on the development of secondary drug resistance because of the paucity of the information. The emergence of multi-drug resistant TB and extensively drug-resistant TB threatens TB control now and in the future. In theory, direct observation of effective medications may reduce the likelihood of acquired drug resistance. Evaluating acquired drug resistance as the primary outcome in a meta-analysis may require greater dependence on observational data, since extensive clinical trials may not be feasible.

The results of our study suggest that in adult patients with smear or culture-positive tuberculosis, there is little evidence that DOT is better than SAT. While there often seems to be some indication that DOT is better than SAT, the observed difference is small. Our analysis found no statistically significant differences between DOT and SAT for the primary indicators of treatment success when considering both RCT and PCS. Only in the stratified analysis of RCT studies is there any evidence of a significant difference between DOT and SAT for the RD of LTFU. We believe our findings further support the current lack of evidence that DOT is superior to SAT for improving cure rates, adherence [2,3], or any other measure of treatment success [4,5]. It is important to note that despite the importance of TB we agree there is a lack of high-quality data available in the literature [8], and while we believe traditional RCTs are unlikely to occur in the future there is a need for more high-quality evidence in the form of prospective cohort and cluster randomized trials [8]. Such studies could provide a clear understanding of the effectiveness of DOT. Additionally, the ability to compare novel means of treatment monitoring should be conducted based on the needs and consideration of the populations being served[53].

In summary, we could not reproduce a previous meta-analysis, due to inconsistencies regarding study inclusion and insufficient information about the analysis methods. Further improvements toward robust reproducibility of meta-analyses, especially those directly related to interventions are needed [54–57]. Our new analysis found that for adult patients 15 years or older with drug-susceptible pulmonary TB, there is no statistically significant difference between DOT and SAT and the magnitude of the difference between DOT and SAT for all reported outcomes is small. The role of DOT in the DOTS strategy remains controversial and we agree with McLaren et al. that further efforts are needed to evaluate its role [8].

## Data sharing statement

Documentation and data for reproducibility are freely available in the supplementary materials.

## Supporting information

S1 Text Supplementary Documentation. This document contains information about all the supplementary materials, explains how to reproduce the results of our analysis, and has additional results not shown in the main text.
(DOCX)

S1 Folder Zip folder. This folder contains all of the files described in the Supplementary Documentation.
(ZIP)
