# Supplementary material for: An attempt to reproduce a previous meta-analysis and a new analysis regarding the impact of directly observed therapy on tuberculosis treatment outcomes
Source: PLoS One. 2019 May 23;14(5):e0217219. doi: 10.1371/journal.pone.0217219 (PMC6532908; doi:10.1371/journal.pone.0217219)
Supplement: S1 Text — This document contains information about all the supplementary materials, explains how to reproduce the results of our analysis, and has additional results not shown in the main text. (DOCX) [file pone.0217219.s001.docx]

**Supplementary Documentation: An attempt to reproduce a previous meta-analysis and a new analysis regarding the impact of directly observed therapy on tuberculosis treatment outcomes**

Brian McKay^1*^, Maria Castellanos^1^, Mark Ebell^1^, Christopher C. Whalen^1^, Andreas Handel^1*^

^1^Department of Epidemiology and Biostatistics, The University of Georgia, Athens, GA, United States of America

**Table of Contents**

[Overview of Supplementary Materials 2](#_Toc5297922)

[Getting all of the files 2](#_Toc5297923)

[Description of all files included in the supplement 2](#_Toc5297924)

[Instructions for reproducing results 3](#_Toc5297925)

[Documentation for the systematic literature review 3](#_Toc5297926)

[Supplementary Results 4](#_Toc5297927)

[Risk difference (RD) forest plots and tables for each outcome 4](#_Toc5297928)

[Lost to follow-up (LTFU) 4](#_Toc5297929)

[Treatment failure 5](#_Toc5297930)

[Completion of treatment 7](#_Toc5297931)

[Death 8](#_Toc5297932)

[Cure 9](#_Toc5297933)

[Sensitivity analysis for each outcome 10](#_Toc5297934)

[Method used for the sensitivity analysis 10](#_Toc5297935)

[Lost to follow-up (LTFU) 11](#_Toc5297936)

[Treatment failure 12](#_Toc5297937)

[Completing treatment 14](#_Toc5297938)

[Death 15](#_Toc5297939)

[Cure 16](#_Toc5297940)

[Publication bias for each outcome 18](#_Toc5297941)

[Lost to follow-up (LTFU) 18](#_Toc5297942)

[Treatment failure 19](#_Toc5297943)

[Completing treatment 20](#_Toc5297944)

[Death 21](#_Toc5297945)

[Cure 22](#_Toc5297946)

[Reproducing the results presented in PG 23](#_Toc5297947)

[Comparision of included studies 23](#_Toc5297948)

[Comparison of data re-abstraction 24](#_Toc5297949)

[Reproducing figures and tables from PG 25](#_Toc5297950)

[Figure 2 from PG 25](#_Toc5297951)

[Table 2 from PG 26](#_Toc5297952)

[Figure 3 from PG 26](#_Toc5297953)

[Table 3 from PG 27](#_Toc5297954)

[Reproducibility of meta-analyses 28](#_Toc5297955)

[References 28](#_Toc5297956)

# Overview of Supplementary Materials

The supplementary materials consist of two parts. One part are the individual files related to the systematic review and analysis. The second part is the supplementary documentation. The contents of the supplementary documentation are presented in the table of contents above.

## Getting all of the files

Files described below are in the supporting information “**S1 Folder**”. This zip file can be downloaded from the PLOS ONE website.

## Description of all files included in the supplement

- The “**Figures**” folder contains all of the figures created for the analysis
- “**1 Data and Anysis.R**” is an R script that installs all of the required packages and completes all the data analysis for the results presented in the manuscript as well as those in the supplementary material
- “**2 PLoSOne Figures.Rmd**” is a Rmarkdown file that generates all of the figures as well as an HTML that shows all of the figures. The figures are saved individually in the “Figures” folder.
- “**3 PLoSOne Supplementary Material.Rmd**” is a Rmarkdown file that generates the Supplementary Documentation word document (the document you are looking at now with some formatting changes).
- “**4 PLoSOne Manuscript.Rmd**” is a Rmarkdown file that generates the published manuscript (the formatting will not be identical).
- “**OriginalLTFU.csv**” is a csv file with the data for lost to follow up as reported in PG (1).
- “**OriginalTXFAIL.csv**” is a csv file with the data for treatment failure as reported in PG (1).
- “**packages.bib**” is a BibTeX file used to create the citations for the R packages used in the analysis.
- “**PRISMA-Checklist**” is a word document detailing where in the manuscript the information required by the PRISMA guidelines are presented.
- “**Systematic Literature Review DOT vs SAT.ods**” is an Open Document Spreadsheet that can be opened with Microsoft excel or Open Office. It provides every detail of the systematic review from start to finish.
- “**TBref1.bib**” is a BibTeX file used to create the article citations for the manuscript and supplementary documentation. (“TBref1.bib.bak” is a file used by JabRef to detect changes)
- “**vancouver.csl**” is a style file that formats the citations using the vancouver style.

## Instructions for reproducing results

To reproduce all results presented in the manuscript and the SM sections below, follow these steps in order:

1. install R, Rstudio, and Pandoc (Pandoc should be installed automatically when Rstudio is installed). Microsoft Word or Open Office Word is also required.
2. Run the R script “1 Data and Analysis.R”. This installs all the required packages and completes the data analysis.
3. Run/knit the R Markdown file “2 Figures.RMD”. This creates all the figures used and generates an HTML that shows all of the figures.
4. Run/knit the R Markdown “3 Supplementary Materials.RMD” generates the supplementary material as Word document.
5. Run/knit the R Markdown “4 Manuscript.RMD” generates the main text and results as a Word document.

**Important note:** All of files included in the “S1 Folder” are required and all of them must be in the same folder. Otherwise new file paths will have to be specified to run the code.

## Documentation for the systematic literature review

An Open Office spread sheet labeled “Systematic Literature Review DOT vs SAT” provides details of the systematic review. Each tab of the spread sheet is a different step of the systematic review, study quality, and data abstraction process completed in parallel by two researchers. The following tabs are included in the spreadsheet:

1. “Search and General Info”: Contains details on the search strategy and general information such as progress and study criteria.
2. “Review of Title”: There are two of these sheets one for each researcher and shows which studies each wanted to include for further review
3. “Results of Title Review”: Shows agreement, disagreement and resolution.
4. “Full Text Review”: There are two of these sheets one for each researcher and shows which studies each wanted to include in the review
5. “Results Studies to Include”: Shows agreement, disagreement, and resolution.
6. “Included Studies From Search”: Studies found during search that have prospective data comparing DOT vs SAT
7. “Included Studies Found in References”: Studies found in citations that have prospective data comparing DOT vs SAT
8. “Final Studies Included”: Studies that meet the desired inclusion criteria
9. “Data Abstraction”: There are two of these sheets one for each researcher and shows data as abstracted by the researchers independently
10. “Results Data Abstraction”: Check to make sure everything lines up.
11. “Observational Study Quality”: There are two of these sheets one for each researcher and shows the assigned quality score for each study
12. “Randomized Trial Study Quality”: There are two of these sheets one for each researcher and shows the assigned quality score for each study
13. “Meta Data for Included Studies”: Study characteristics for the included studies
14. “Comparison of Jadad Scores and CRBCS Scores”: Shows the impact of using a more appropriate quality assessment
15. “Comparison of Repeated Data Abstraction of PG”: Re-abstracted the data from studies included by PG and highlight the areas of disagreement and possible reasons

# Supplementary Results

The following section shows additional results not shown in the main text.

## Risk difference (RD) forest plots and tables for each outcome

### Lost to follow-up (LTFU)

Figure A and Table A shows results for lost to follow-up RD. The pooled RD is 3.73 (95% CI -1.2, 8.66).


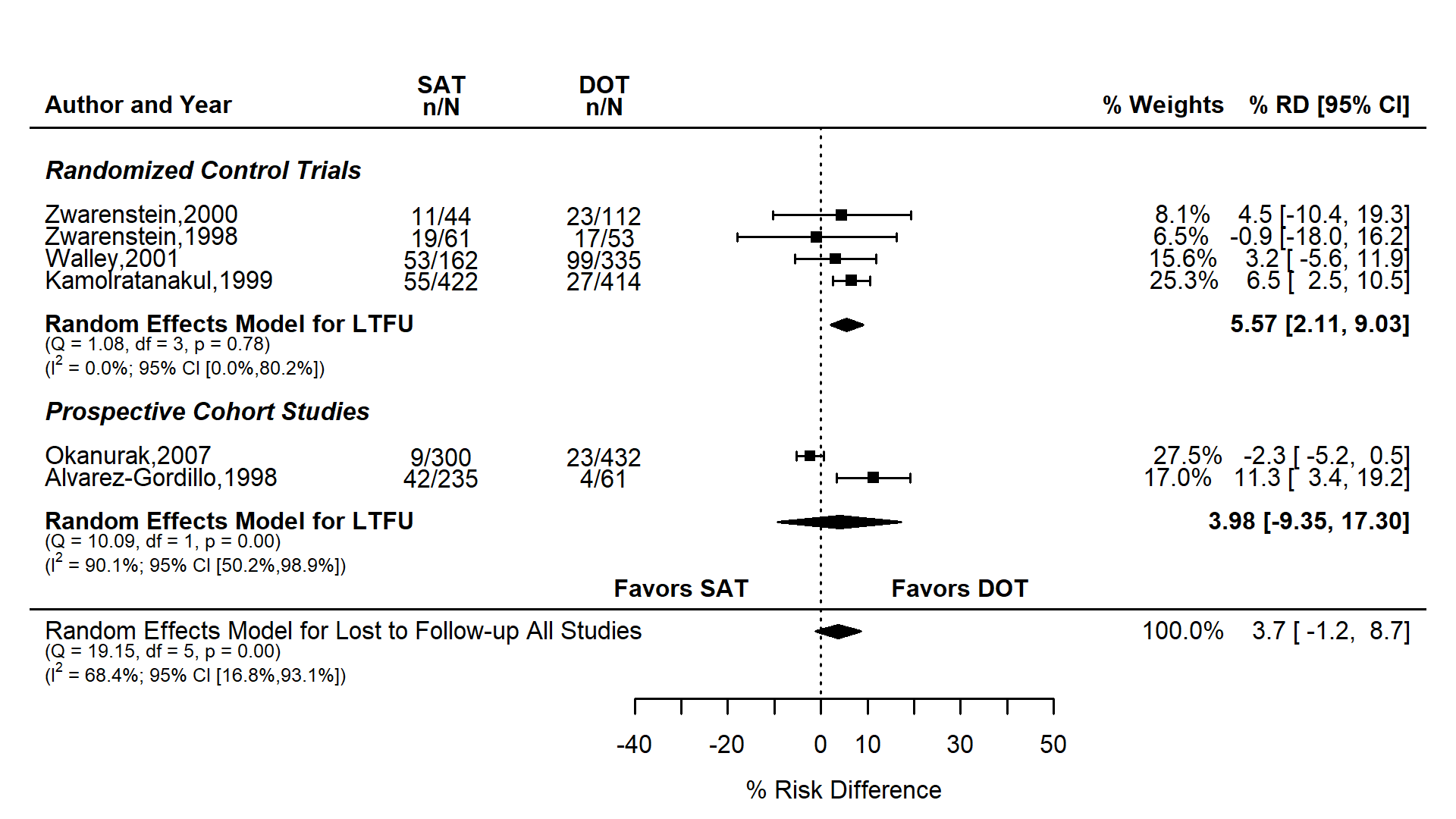


Figure A Lost to Follow-up risk difference between DOT and SAT stratified by study design. Q = Cochrane’s Q; df = Degrees of Freedom; p= P-value associated with Q; I^2= Proportion of variation due to heterogeneity and corresponding 95% confidence interval

Table A **Risk of Loss to Follow-up SAT vs DOT**

| Study | DOT Risk (%) | 95%CI Lower | 95%CI Upper | DOT Weights (%) | SAT Risk (%) | 95%CI Lower | 95%CI Upper | SAT Weights (%) |
| --- | --- | --- | --- | --- | --- | --- | --- | --- |
| **Randomized Controlled Trials** |  |  |  |  |  |  |  |  |
| Zwarenstein 2000 | 20.54 | 13.05 | 28.02 | 89.59 | 25 | 12.21 | 37.79 | 51.19 |
| Zwarenstein 1998 | 32.08 | 19.51 | 44.64 | 10.41 | 31.15 | 19.53 | 42.77 | 48.81 |
| Walley | 29.55 | 24.67 | 34.44 | 24.96 | 32.72 | 25.49 | 39.94 | 20.69 |
| Kamolratanakul | 6.52 | 4.14 | 8.9 | 26.52 | 13.03 | 9.82 | 16.25 | 26.93 |
| **Pooled Risk Estimate RCT** | 21.5 | 9.83 | 33.17 |  | 24.8 | 14.82 | 34.77 |  |
| **Heterogeneity I^2 RCT** | 94.51 |  |  |  | 85 |  |  |  |
| **Prospective Cohort Study** |  |  |  |  |  |  |  |  |
| Okanurak | 5.32 | 3.21 | 7.44 | 21.03 | 3 | 1.07 | 4.93 | 22 |
| Alvarez-Gordillo | 6.56 | 0.35 | 12.77 | 27.49 | 17.87 | 12.97 | 22.77 | 30.38 |
| **Pooled Risk Estimate PCS** | 5.45 | 3.45 | 7.46 |  | 10.26 | -4.31 | 24.83 |  |
| **Heterogeneity I^2 PCS** | 0 |  |  |  | 96.74 |  |  |  |
| **Overall Effect** | 16.11 | 6.49 | 25.72 |  | 19.66 | 10.21 | 29.1 |  |

###

### Treatment failure

Figure B and Table B shows the results of the analysis considering the RD of treatment failure as an outcome. The pooled RD is 0.46 (95% CI -1.02, 1.93).


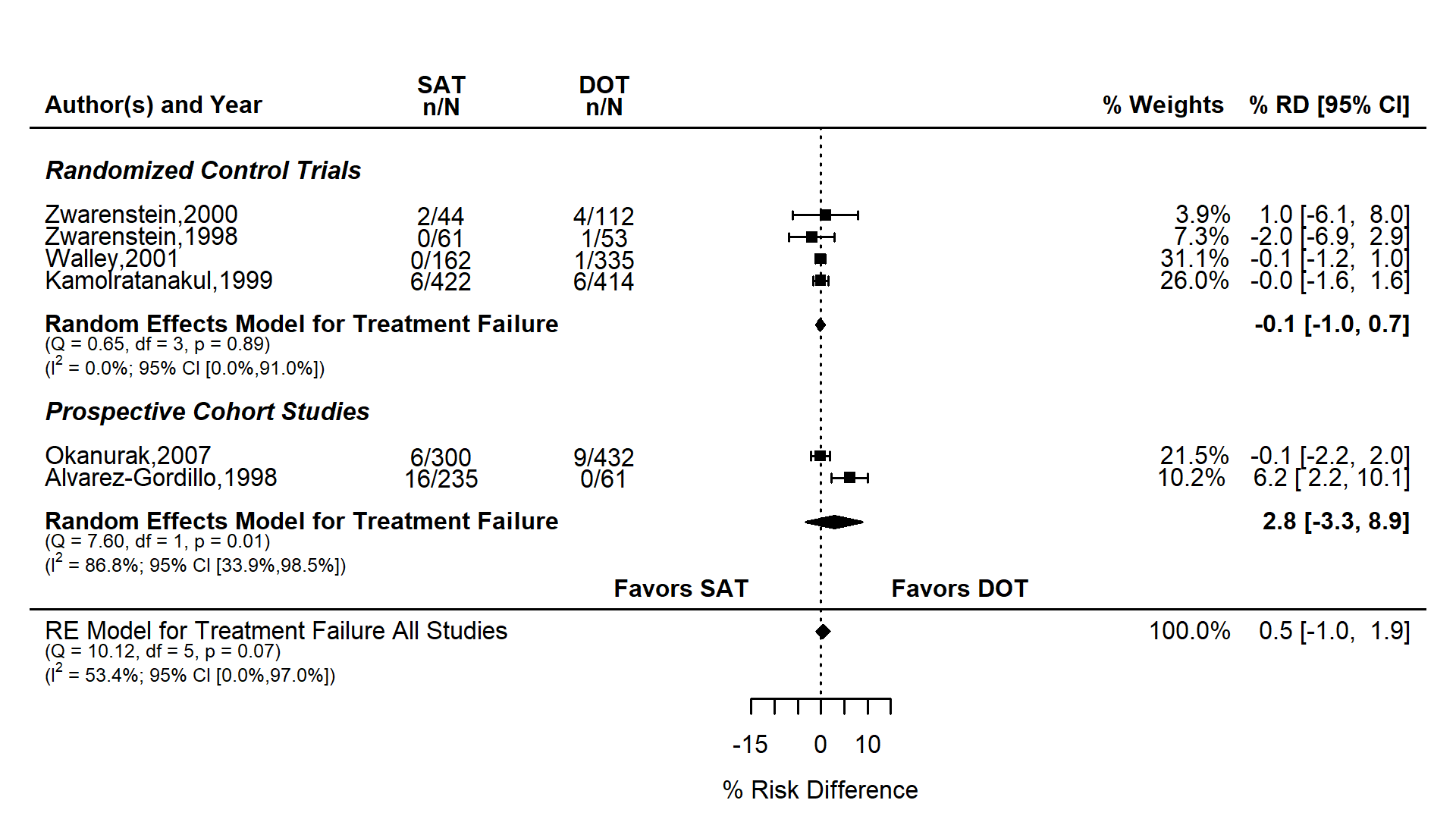


Figure B Treatment Failure risk difference between DOT and SAT stratified by study design. Q = Cochrane’s Q; df = Degrees of Freedom; p= P-value associated with Q; I^2= Proportion of variation due to heterogeneity and corresponding 95% confidence interval

Table B **Risk of Treatment Failure SAT vs DOT**

| Study | DOT Risk (%) | 95%CI Lower | 95%CI Upper | DOT Weights (%) | SAT Risk (%) | 95%CI Lower | 95%CI Upper | SAT Weights (%) |
| --- | --- | --- | --- | --- | --- | --- | --- | --- |
| **Randomized Controlled Trials** |  |  |  |  |  |  |  |  |
| Zwarenstein 2000 | 3.57 | 0.13 | 7.01 | 73.21 | 4.55 | -1.61 | 10.7 | 54.43 |
| Zwarenstein 1998 | 1.89 | -1.78 | 5.55 | 26.79 | 0.81 | -1.42 | 3.03 | 45.57 |
| Walley | 0.3 | -0.29 | 0.88 | 9.46 | 0.31 | -0.54 | 1.16 | 1.93 |
| Kamolratanakul | 1.45 | 0.3 | 2.6 | 46.97 | 1.42 | 0.29 | 2.55 | 49.1 |
| **Pooled Risk Estimate RCT** | 1.15 | -0.02 | 2.31 |  | 0.85 | -0.01 | 1.72 |  |
| **Heterogeneity I^2 RCT** | 55.39 |  |  |  | 26.14 |  |  |  |
| **Prospective Cohort Study** |  |  |  |  |  |  |  |  |
| Okanurak | 2.08 | 0.74 | 3.43 | 8.51 | 2 | 0.42 | 3.58 | 12.98 |
| Alvarez-Gordillo | 0.81 | -1.42 | 3.03 | 35.05 | 6.81 | 3.59 | 10.03 | 35.98 |
| **Pooled Risk Estimate PCS** | 1.74 | 0.59 | 2.89 |  | 4.19 | -0.5 | 8.89 |  |
| **Heterogeneity I^2 PCS** | 0 |  |  |  | 85.5 |  |  |  |
| **Overall Effect** | 1.26 | 0.38 | 2.15 |  | 2.06 | 0.35 | 3.78 |  |

###

### Completion of treatment

Results from the analysis are shown in Figure C and Table C. The pooled RD is -2.08 (95% CI -8.03, 3.87).


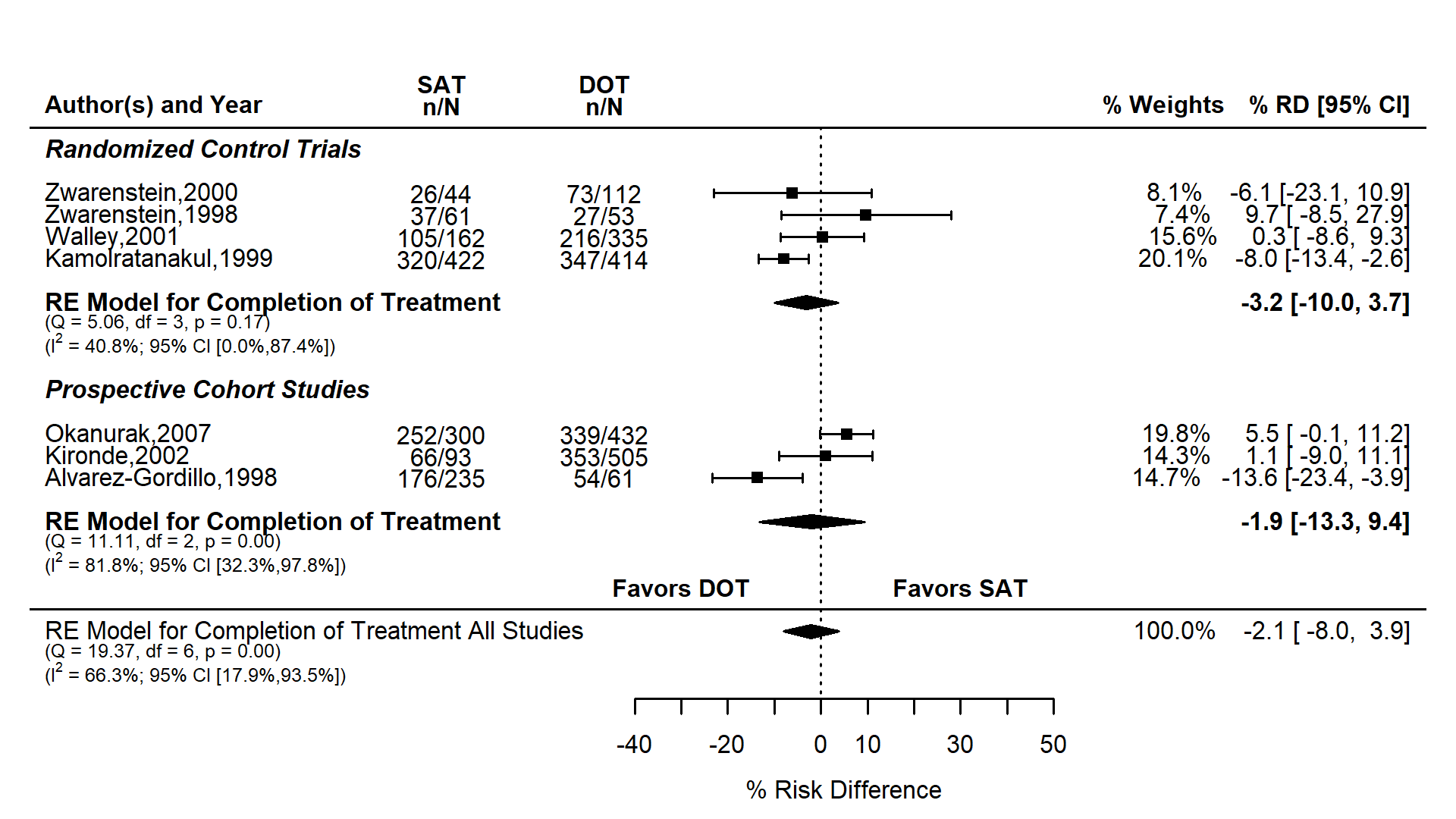


Figure C Completion of Treatment risk difference between DOT and SAT stratified by study design. Q = Cochrane’s Q; df = Degrees of Freedom; p= P-value associated with Q; I^2= Proportion of variation due to heterogeneity and corresponding 95% confidence interval

Table C **Risk of Completion of Treatment SAT vs DOT**

| Study | DOT Risk (%) | 95%CI Lower | 95%CI Upper | DOT Weights (%) | SAT Risk (%) | 95%CI Lower | 95%CI Upper | SAT Weights (%) |
| --- | --- | --- | --- | --- | --- | --- | --- | --- |
| **Randomized Controlled Trials** |  |  |  |  |  |  |  |  |
| Kamolratanakul | 65.18 | 56.36 | 74 | 35.02 | 59.09 | 44.56 | 73.62 | 38.27 |
| Zwarenstein 1998 | 50.94 | 37.48 | 64.4 | 34.9 | 60.66 | 48.4 | 72.91 | 26.55 |
| Walley | 64.48 | 59.35 | 69.6 | 30.08 | 64.81 | 57.46 | 72.17 | 35.18 |
| Zwarenstein 2000 | 83.82 | 80.27 | 87.36 | 26.64 | 75.83 | 71.74 | 79.91 | 28.55 |
| **Pooled Risk Estimate RCT** | 67.01 | 53.89 | 80.12 |  | 66.71 | 58.43 | 74.98 |  |
| **Heterogeneity I^2 RCT** | 94.11 |  |  |  | 73.7 |  |  |  |
| **Prospective Cohort Study** |  |  |  |  |  |  |  |  |
| Okanurak | 78.47 | 74.6 | 82.35 | 24.67 | 84 | 79.85 | 88.15 | 17.25 |
| Kironde | 69.9 | 65.9 | 73.9 | 21.48 | 70.97 | 61.74 | 80.19 | 20.37 |
| Alvarez-Gordillo | 88.52 | 80.53 | 96.52 | 27.21 | 74.89 | 69.35 | 80.44 | 33.82 |
| **Pooled Risk Estimate PCS** | 78.5 | 68.28 | 88.73 |  | 77.34 | 69.66 | 85.01 |  |
| **Heterogeneity I^2 PCS** | 92.37 |  |  |  | 79.77 |  |  |  |
| **Overall Effect** | 72.3 | 63.38 | 81.21 |  | 71.47 | 64.96 | 77.98 |  |

###

### Death

Figure D and Table D shows the results. The pooled RD is -0.1 (95% CI -2.08, 1.87).


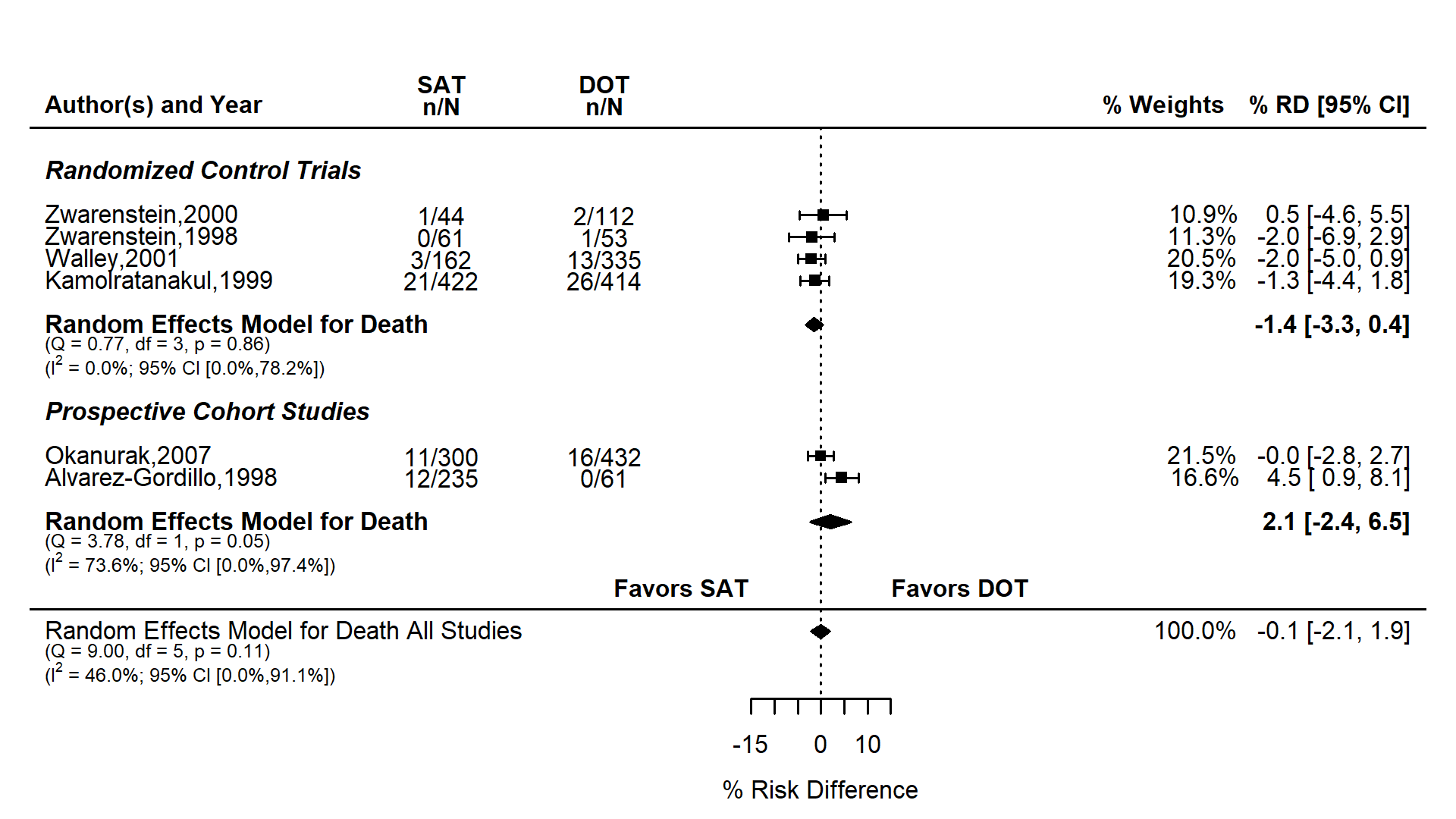


Figure D Death risk difference between DOT and SAT stratified by study design. Q = Cochrane’s Q; df = Degrees of Freedom; p= P-value associated with Q; I^2= Proportion of variation due to heterogeneity and corresponding 95% confidence interval

Table D **Risk of Death SAT vs DOT**

| Study | DOT Risk (%) | 95%CI Lower | 95%CI Upper | DOT Weights (%) | SAT Risk (%) | 95%CI Lower | 95%CI Upper | SAT Weights (%) |
| --- | --- | --- | --- | --- | --- | --- | --- | --- |
| **Randomized Controlled Trials** |  |  |  |  |  |  |  |  |
| Zwarenstein 2000 | 1.79 | -0.67 | 4.24 | 52.77 | 2.27 | -2.13 | 6.68 | 63.65 |
| Zwarenstein 1998 | 1.89 | -1.78 | 5.55 | 47.23 | 0.81 | -1.42 | 3.03 | 36.35 |
| Walley | 3.88 | 1.81 | 5.95 | 26.02 | 1.85 | -0.22 | 3.93 | 14 |
| Kamolratanakul | 6.28 | 3.94 | 8.62 | 29.03 | 4.98 | 2.9 | 7.05 | 29.09 |
| **Pooled Risk Estimate RCT** | 3.62 | 1.53 | 5.71 |  | 2.53 | 0.51 | 4.55 |  |
| **Heterogeneity I^2 RCT** | 62.89 |  |  |  | 62.04 |  |  |  |
| **Prospective Cohort Study** |  |  |  |  |  |  |  |  |
| Okanurak | 3.7 | 1.92 | 5.48 | 18.05 | 3.67 | 1.54 | 5.79 | 27.81 |
| Alvarez-Gordillo | 0.81 | -1.42 | 3.03 | 26.91 | 5.11 | 2.29 | 7.92 | 29.1 |
| **Pooled Risk Estimate PCS** | 2.34 | -0.5 | 5.17 |  | 4.19 | 2.49 | 5.89 |  |
| **Heterogeneity I^2 PCS** | 74.79 |  |  |  | 0 |  |  |  |
| **Overall Effect** | 3.15 | 1.56 | 4.74 |  | 3.14 | 1.64 | 4.63 |  |

###

### Cure

Figure E and Table E shows the result of our analysis. The pooled RD is -3.99 (95% CI -13.11, 5.13).


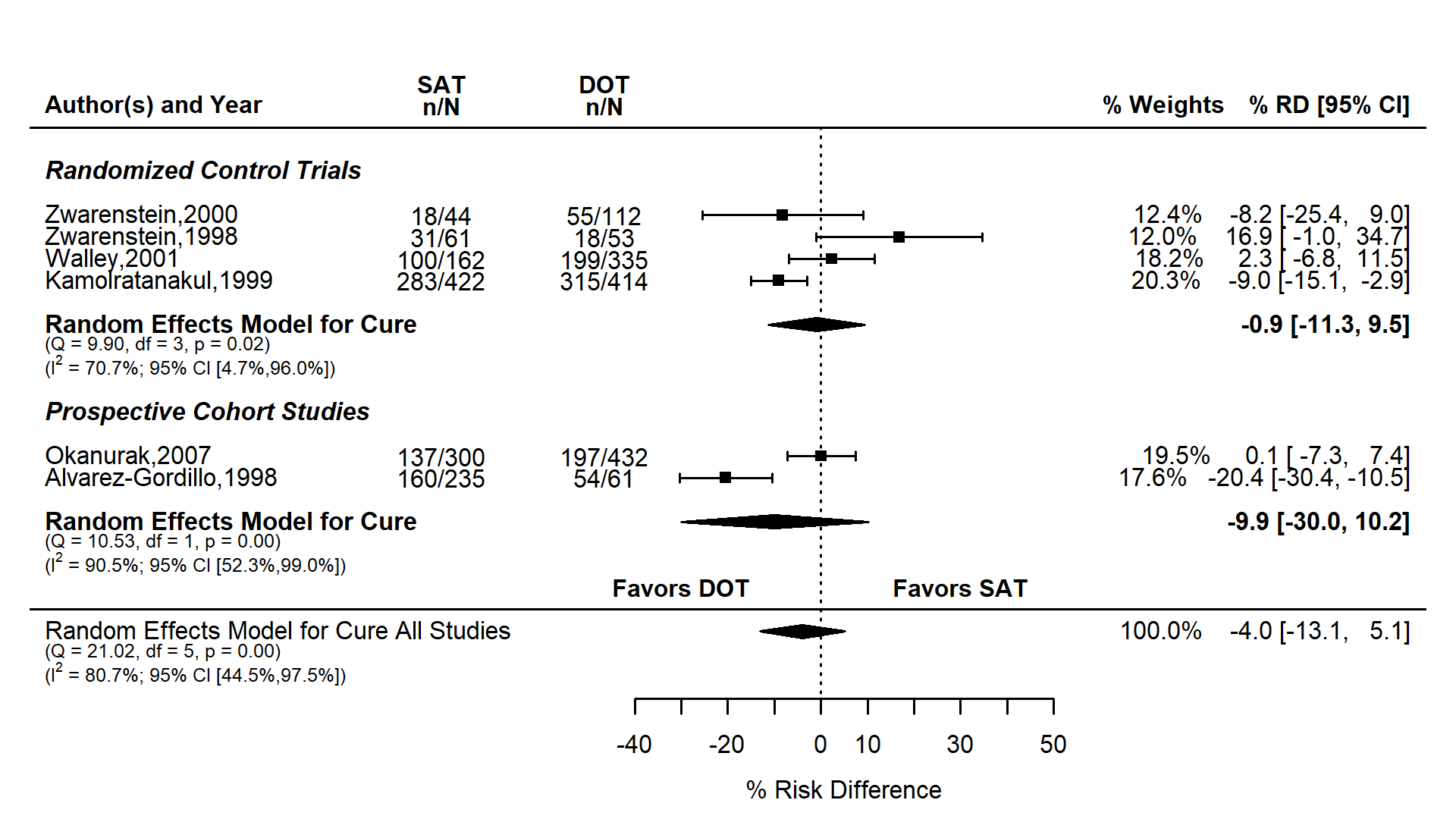


Figure E Cure risk difference between DOT and SAT stratified by study design. Q = Cochrane’s Q; df = Degrees of Freedom; p= P-value associated with Q; I^2= Proportion of variation due to heterogeneity and corresponding 95% confidence interval

Table E **Risk of Cure SAT vs DOT**

| Study | DOT Risk (%) | 95%CI Lower | 95%CI Upper | DOT Weights (%) | SAT Risk (%) | 95%CI Lower | 95%CI Upper | SAT Weights (%) |
| --- | --- | --- | --- | --- | --- | --- | --- | --- |
| **Randomized Controlled Trials** |  |  |  |  |  |  |  |  |
| Zwarenstein 2000 | 49.11 | 39.85 | 58.37 | 50.3 | 40.91 | 26.38 | 55.44 | 50.1 |
| Zwarenstein 1998 | 33.96 | 21.21 | 46.71 | 49.7 | 50.82 | 38.27 | 63.37 | 49.9 |
| Walley | 59.4 | 54.14 | 64.66 | 24.68 | 61.73 | 54.24 | 69.21 | 20.34 |
| Kamolratanakul | 76.09 | 71.98 | 80.2 | 25.94 | 67.06 | 62.58 | 71.55 | 27.45 |
| **Pooled Risk Estimate RCT** | 55.33 | 38.15 | 72.51 |  | 56.65 | 45.62 | 67.68 |  |
| **Heterogeneity I^2 RCT** | 96.24 |  |  |  | 84.54 |  |  |  |
| **Prospective Cohort Study** |  |  |  |  |  |  |  |  |
| Okanurak | 45.6 | 40.91 | 50.3 | 23.19 | 45.67 | 40.03 | 51.3 | 22.34 |
| Alvarez-Gordillo | 88.52 | 80.53 | 96.52 | 26.19 | 68.09 | 62.13 | 74.04 | 29.87 |
| **Pooled Risk Estimate PCS** | 66.94 | 24.87 | 109 |  | 56.85 | 34.88 | 78.82 |  |
| **Heterogeneity I^2 PCS** | 98.78 |  |  |  | 96.51 |  |  |  |
| **Overall Effect** | 59.08 | 43.02 | 75.15 |  | 56.66 | 47.56 | 65.75 |  |

##

## Sensitivity analysis for each outcome

### Method used for the sensitivity analysis

Sensitivity analysis used the **leave1out()** function in the metafor package. The function removes a single study from analysis and recalculates all of the relevant statistics. A table is produced showing the study that has been removed and the corresponding changes in the results. Outlier and influential studies were identified using the **influence()** function in the metafor package. We used externally studentized residual to identify studies that may be an outlier. We then used the difference in fits (DFFITS), Cook’s distance, covariance ratio, and the standard deviation of the estimated coefficient (DFBETAS) to identify studies that were influential. Studies may be considered influential if any of the following are true.

- DFFITS: values larger than $3\sqrt{p/k-p}$ where $p$ is the number of model coefficients and $k$ the number of studies
- Cook’s: if the lower tail area of a chi-square distribution with $p$ degrees of freedom is larger then $50\%$
- Covariance Ratio: hat values larger than $3(p/k)$
- DFBETAS: values larger than 1

If the overall results for an outcome changes when a single study that is considered to be both an outlier and influential is removed the robustness of the results are called into question. More information on the implementation of these measures can be found in the following paper ^(2)^.

### Lost to follow-up (LTFU)

The study by Kamolratanakul ^(3)^ may be an influential outlier when measuring the RR of lost to follow-up due to the large residuals and Cook’s distance (Table F). The estimated RR is not sensitive to the exclusion of any single study (Table H).

The study by Okanurak ^(4)^ may be an influential outlier when measuring the RD of lost to follow-up due to the large residuals and Cook’s distance (Table G). The overall estimated RD is not significant when all of the studies are included but when one study ^(4)^ is removed the overall estimate of the RD is significant (Table I). The RD results for lost to follow-up may be not robust.

Table F **RR Lost to Follow-up Outlier and Influence Analysis**

|  | Studentized Res | DFFITS | Cook’s Distance | Covariance Ratio | DFBETAS | Influence |
| --- | --- | --- | --- | --- | --- | --- |
| Alvarez-Gordillo,1998 | 1.31 | 0.41 | 0.16 | 1.03 | 0.41 | No |
| Kamolratanakul,1999 | 2.43 | 1.24 | 0.51 | 0.38 | 1.28 | Yes |
| Okanurak,2007 | -1.78 | -0.60 | 0.29 | 0.83 | -0.63 | No |
| Walley,2001 | -0.27 | -0.14 | 0.03 | 1.84 | -0.15 | No |
| Zwarenstein,1998 | -0.57 | -0.26 | 0.09 | 1.50 | -0.26 | No |
| Zwarenstein,2000 | 0.00 | 0.00 | 0.00 | 1.59 | 0.00 | No |

Table G **RD Lost to Follow-up Outlier and Influence Analysis**

|  | Studentized Res | DFFITS | Cook’s Distance | Covariance Ratio | DFBETAS | Influence |
| --- | --- | --- | --- | --- | --- | --- |
| Alvarez-Gordillo,1998 | 1.49 | 0.69 | 0.41 | 0.98 | 0.71 | No |
| Kamolratanakul,1999 | 0.58 | 0.31 | 0.11 | 1.50 | 0.31 | No |
| Okanurak,2007 | -4.05 | -3.61 | 1.21 | 0.41 | -2.55 | Yes |
| Walley,2001 | -0.10 | -0.07 | 0.01 | 1.42 | -0.07 | No |
| Zwarenstein,1998 | -0.49 | -0.15 | 0.02 | 1.15 | -0.14 | No |
| Zwarenstein,2000 | 0.08 | 0.01 | 0.00 | 1.20 | 0.01 | No |

Table H **RR Lost to Follow-up Sensitivity Analysis:** **pval** = P-value associated with estimate; **ci.lb** = Confidence interval lower bound; **ci.up** = Confidence interval upper bound; **I2** = Estimated heterogeneity; **Qp** = P-value associated with heterogeneity

| Excluded Study | Estimated RR | pval | ci.lb | ci.ub | I2 | Qp |
| --- | --- | --- | --- | --- | --- | --- |
| Alvarez-Gordillo,1998 | 1.15 | 0.43 | 0.81 | 1.62 | 60.73 | 0.05 |
| Kamolratanakul,1999 | 1.09 | 0.44 | 0.88 | 1.34 | 0.00 | 0.19 |
| Okanurak,2007 | 1.35 | 0.06 | 0.99 | 1.84 | 48.68 | 0.10 |
| Walley,2001 | 1.27 | 0.31 | 0.80 | 2.01 | 64.13 | 0.03 |
| Zwarenstein,1998 | 1.29 | 0.23 | 0.85 | 1.96 | 67.11 | 0.02 |
| Zwarenstein,2000 | 1.23 | 0.34 | 0.80 | 1.89 | 70.95 | 0.02 |

Table I **RD Lost to Follow-up Sensitivity Analysis:** **pval** = P-value associated with estimate; **ci.lb** = Confidence interval lower bound; **ci.up** = Confidence interval upper bound; **I2** = Estimated heterogeneity; **Qp** = P-value associated with heterogeneity

| Excluded Study | Estimated RD | pval | ci.lb | ci.ub | I2 | Qp |
| --- | --- | --- | --- | --- | --- | --- |
| Alvarez-Gordillo,1998 | 2.12 | 0.39 | -2.76 | 7.00 | 62.56 | 0.01 |
| Kamolratanakul,1999 | 2.89 | 0.35 | -3.16 | 8.94 | 60.20 | 0.02 |
| Okanurak,2007 | 6.49 | 0.00 | 3.32 | 9.66 | 0.00 | 0.60 |
| Walley,2001 | 3.91 | 0.19 | -1.97 | 9.79 | 75.20 | 0.00 |
| Zwarenstein,1998 | 4.10 | 0.13 | -1.19 | 9.39 | 74.29 | 0.00 |
| Zwarenstein,2000 | 3.70 | 0.18 | -1.70 | 9.10 | 74.72 | 0.00 |

###

### Treatment failure

When measuring RR, none of the studies are indicated as possibly influential outliers (Table J). The overall estimated RR is not significant when all of the studies are included, and this did not change when any single study was removed (Table L).

The study by Alvarez-Gordillo ^(5)^ was shown to be an outlier and possibly influential when measuring the RD of treatment failure based on the residuals and Cook’s distance (Table K). The overall estimated RD is not significant when all of the studies are included, and this did not change when any single study was removed (Table M).

Interestingly when the study by Alvarez-Gordillo ^(5)^ was removed, the direction of effect changed from favoring DOT to favoring SAT for both RR and RD.

Table J **RR Treatment Failure Outlier and Influence Analysis**

|  | Studentized Res | DFFITS | Cook’s Distance | Covariance Ratio | DFBETAS | Influence |
| --- | --- | --- | --- | --- | --- | --- |
| Alvarez-Gordillo,1998 | 1.49 | 0.33 | 0.11 | 1.05 | 0.33 | No |
| Kamolratanakul,1999 | -0.23 | -0.16 | 0.03 | 1.48 | -0.16 | No |
| Okanurak,2007 | -0.26 | -0.21 | 0.04 | 1.63 | -0.21 | No |
| Walley,2001 | -0.29 | -0.06 | 0.00 | 1.04 | -0.06 | No |
| Zwarenstein,1998 | -0.83 | -0.16 | 0.03 | 1.04 | -0.16 | No |
| Zwarenstein,2000 | 0.35 | 0.16 | 0.02 | 1.20 | 0.16 | No |

Table K **RD Treatment Failure Outlier and Influence Analysis**

|  | Studentized Res | DFFITS | Cook’s Distance | Covariance Ratio | DFBETAS | Influence |
| --- | --- | --- | --- | --- | --- | --- |
| Alvarez-Gordillo,1998 | 3.08 | 0.93 | 0.63 | 0.31 | 1.45 | Yes |
| Kamolratanakul,1999 | -0.33 | -0.33 | 0.30 | 2.95 | -0.36 | No |
| Okanurak,2007 | -0.34 | -0.35 | 0.28 | 2.81 | -0.36 | No |
| Walley,2001 | -0.40 | -0.36 | 0.37 | 2.98 | -0.41 | No |
| Zwarenstein,1998 | -0.88 | -0.47 | 0.26 | 1.70 | -0.41 | No |
| Zwarenstein,2000 | 0.11 | -0.10 | 0.01 | 1.52 | -0.09 | No |

Table L **RR Treatment Failure Sensitivity Analysis:** **pval** = P-value associated with estimate; **ci.lb** = Confidence interval lower bound; **ci.up** = Confidence interval upper bound; **I2** = Estimated heterogeneity; **Qp** = P-value associated with heterogeneity

| Excluded Study | Estimated RR | pval | ci.lb | ci.ub | I2 | Qp |
| --- | --- | --- | --- | --- | --- | --- |
| Alvarez-Gordillo,1998 | 0.98 | 0.96 | 0.52 | 1.84 | 0 | 0.94 |
| Kamolratanakul,1999 | 1.15 | 0.72 | 0.54 | 2.42 | 0 | 0.56 |
| Okanurak,2007 | 1.16 | 0.70 | 0.53 | 2.55 | 0 | 0.56 |
| Walley,2001 | 1.11 | 0.74 | 0.59 | 2.08 | 0 | 0.57 |
| Zwarenstein,1998 | 1.15 | 0.67 | 0.61 | 2.15 | 0 | 0.67 |
| Zwarenstein,2000 | 1.04 | 0.91 | 0.53 | 2.03 | 0 | 0.57 |

Table M **RD Treatment Failure Sensitivity Analysis:** **pval** = P-value associated with estimate; **ci.lb** = Confidence interval lower bound; **ci.up** = Confidence interval upper bound; **I2** = Estimated heterogeneity; **Qp** = P-value associated with heterogeneity

| Excluded Study | Estimated RD | pval | ci.lb | ci.ub | I2 | Qp |
| --- | --- | --- | --- | --- | --- | --- |
| Alvarez-Gordillo,1998 | -0.14 | 0.74 | -0.96 | 0.68 | 0.00 | 0.96 |
| Kamolratanakul,1999 | 0.87 | 0.50 | -1.66 | 3.40 | 72.95 | 0.04 |
| Okanurak,2007 | 0.86 | 0.50 | -1.61 | 3.33 | 76.86 | 0.04 |
| Walley,2001 | 0.92 | 0.48 | -1.63 | 3.46 | 68.99 | 0.05 |
| Zwarenstein,1998 | 0.84 | 0.39 | -1.08 | 2.76 | 72.43 | 0.05 |
| Zwarenstein,2000 | 0.54 | 0.56 | -1.28 | 2.36 | 71.17 | 0.04 |

###

### Completing treatment

The study by Okanurak ^(4)^ was shown to be a possible influential outlier when measuring the RR and RD of treatment completion based on residuals and Cook’s distance (Table N and O). The overall estimated RR and RD are not significant when all of the studies are included, and this did not change when ^(4)^ was removed (Table P and Q).

Table N **RR Treatment Completed Outlier and Influence Analysis**

|  | Studentized Res | DFFITS | Cook’s Distance | Covariance Ratio | DFBETAS | Influence |
| --- | --- | --- | --- | --- | --- | --- |
| Alvarez-Gordillo,1998 | -1.60 | -0.69 | 0.39 | 0.98 | -0.69 | No |
| Kamolratanakul,1999 | -0.92 | -0.49 | 0.24 | 1.31 | -0.49 | No |
| Kironde,2002 | 0.40 | 0.15 | 0.03 | 1.34 | 0.15 | No |
| Okanurak,2007 | 1.89 | 1.25 | 0.77 | 0.79 | 1.15 | Yes |
| Walley,2001 | 0.32 | 0.12 | 0.02 | 1.36 | 0.12 | No |
| Zwarenstein,1998 | 1.11 | 0.25 | 0.06 | 1.04 | 0.25 | No |
| Zwarenstein,2000 | -0.44 | -0.12 | 0.02 | 1.14 | -0.12 | No |

Table O **RD Treatment Completed Outlier and Influence Analysis**

|  | Studentized Res | DFFITS | Cook’s Distance | Covariance Ratio | DFBETAS | Influence |
| --- | --- | --- | --- | --- | --- | --- |
| Alvarez-Gordillo,1998 | -1.76 | -0.69 | 0.38 | 0.91 | -0.70 | No |
| Kamolratanakul,1999 | -0.98 | -0.49 | 0.24 | 1.25 | -0.49 | No |
| Kironde,2002 | 0.39 | 0.15 | 0.03 | 1.35 | 0.15 | No |
| Okanurak,2007 | 1.55 | 0.87 | 0.50 | 0.93 | 0.83 | Yes |
| Walley,2001 | 0.31 | 0.12 | 0.02 | 1.40 | 0.12 | No |
| Zwarenstein,1998 | 1.10 | 0.31 | 0.10 | 1.07 | 0.31 | No |
| Zwarenstein,2000 | -0.39 | -0.13 | 0.02 | 1.20 | -0.12 | No |

Table P **RR Treatment Completed Sensitivity Analysis:** **pval** = P-value associated with estimate; **ci.lb** = Confidence interval lower bound; **ci.up** = Confidence interval upper bound; **I2** = Estimated heterogeneity; **Qp** = P-value associated with heterogeneity

| Excluded Study | Estimated RR | pval | ci.lb | ci.ub | I2 | Qp |
| --- | --- | --- | --- | --- | --- | --- |
| Alvarez-Gordillo,1998 | 1.00 | 0.91 | 0.92 | 1.08 | 55.55 | 0.02 |
| Kamolratanakul,1999 | 0.99 | 0.83 | 0.90 | 1.08 | 56.73 | 0.03 |
| Kironde,2002 | 0.96 | 0.44 | 0.88 | 1.06 | 69.35 | 0.00 |
| Okanurak,2007 | 0.94 | 0.07 | 0.87 | 1.00 | 33.18 | 0.19 |
| Walley,2001 | 0.97 | 0.46 | 0.88 | 1.06 | 69.62 | 0.00 |
| Zwarenstein,1998 | 0.96 | 0.34 | 0.89 | 1.04 | 67.21 | 0.00 |
| Zwarenstein,2000 | 0.98 | 0.57 | 0.90 | 1.06 | 69.44 | 0.00 |

Table Q **RD Treatment Completed Sensitivity Analysis:** **pval** = P-value associated with estimate; **ci.lb** = Confidence interval lower bound; **ci.up** = Confidence interval upper bound; **I2** = Estimated heterogeneity; **Qp** = P-value associated with heterogeneity

| Excluded Study | Estimated RD | pval | ci.lb | ci.ub | I2 | Qp |
| --- | --- | --- | --- | --- | --- | --- |
| Alvarez-Gordillo,1998 | -0.20 | 0.95 | -5.87 | 5.47 | 57.24 | 0.02 |
| Kamolratanakul,1999 | -0.59 | 0.86 | -7.24 | 6.05 | 59.92 | 0.03 |
| Kironde,2002 | -2.58 | 0.46 | -9.50 | 4.33 | 71.51 | 0.00 |
| Okanurak,2007 | -4.23 | 0.15 | -9.97 | 1.50 | 47.82 | 0.09 |
| Walley,2001 | -2.49 | 0.49 | -9.53 | 4.54 | 71.37 | 0.00 |
| Zwarenstein,1998 | -3.02 | 0.34 | -9.19 | 3.14 | 69.06 | 0.00 |
| Zwarenstein,2000 | -1.69 | 0.61 | -8.21 | 4.83 | 72.08 | 0.00 |

###

### Death

The study by Kamolratanakul ^(3)^ was indicated as possibly an influential outlier when measuring the RR of death based on the covariance ratio (Table R). The overall estimated RR is not significant when all of the studies are included this did not change when any single study was removed (Table T).

The study by Alvarez-Gordillo ^(5)^ was indicated as possibly an influential outlier when measuring the RD of death based on the residuals and cooks distance (Table S). The overall estimated RD is not significant when all of the studies are included this did not change when any single study was removed (Table U).

Table R **RR Death Outlier and Influence Analysis**

|  | Studentized Res | DFFITS | Cook’s Distance | Covariance Ratio | DFBETAS | Influence |
| --- | --- | --- | --- | --- | --- | --- |
| Alvarez-Gordillo,1998 | 1.43 | 0.20 | 0.04 | 1.02 | 0.20 | No |
| Kamolratanakul,1999 | -0.37 | -0.38 | 0.14 | 2.07 | -0.38 | Yes |
| Okanurak,2007 | 0.50 | 0.32 | 0.10 | 1.41 | 0.32 | No |
| Walley,2001 | -0.85 | -0.31 | 0.09 | 1.13 | -0.31 | No |
| Zwarenstein,1998 | -0.67 | -0.08 | 0.01 | 1.02 | -0.08 | No |
| Zwarenstein,2000 | 0.57 | 0.12 | 0.01 | 1.04 | 0.12 | No |

Table S **RD Death Outlier and Influence Analysis**

|  | Studentized Res | DFFITS | Cook’s Distance | Covariance Ratio | DFBETAS | Influence |
| --- | --- | --- | --- | --- | --- | --- |
| Alvarez-Gordillo,1998 | 2.75 | 1.22 | 0.83 | 0.60 | 1.28 | Yes |
| Kamolratanakul,1999 | -0.53 | -0.27 | 0.09 | 1.49 | -0.27 | No |
| Okanurak,2007 | 0.02 | 0.00 | 0.00 | 1.66 | 0.00 | No |
| Walley,2001 | -0.96 | -0.49 | 0.24 | 1.28 | -0.49 | No |
| Zwarenstein,1998 | -0.64 | -0.25 | 0.07 | 1.27 | -0.24 | No |
| Zwarenstein,2000 | 0.19 | 0.06 | 0.00 | 1.33 | 0.06 | No |

Table T **RR Death Sensitivity Analysis:** **pval** = P-value associated with estimate; **ci.lb** = Confidence interval lower bound; **ci.up** = Confidence interval upper bound; **I2** = Estimated heterogeneity; **Qp** = P-value associated with heterogeneity

| Excluded Study | Estimated RR | pval | ci.lb | ci.ub | I2 | Qp |
| --- | --- | --- | --- | --- | --- | --- |
| Alvarez-Gordillo,1998 | 0.82 | 0.33 | 0.55 | 1.23 | 0 | 0.81 |
| Kamolratanakul,1999 | 0.92 | 0.79 | 0.52 | 1.64 | 0 | 0.48 |
| Okanurak,2007 | 0.80 | 0.36 | 0.50 | 1.28 | 0 | 0.49 |
| Walley,2001 | 0.91 | 0.66 | 0.60 | 1.39 | 0 | 0.57 |
| Zwarenstein,1998 | 0.87 | 0.50 | 0.58 | 1.30 | 0 | 0.53 |
| Zwarenstein,2000 | 0.84 | 0.39 | 0.56 | 1.25 | 0 | 0.51 |

Table U **RD Death Sensitivity Analysis:** **pval** = P-value associated with estimate; **ci.lb** = Confidence interval lower bound; **ci.up** = Confidence interval upper bound; **I2** = Estimated heterogeneity; **Qp** = P-value associated with heterogeneity

| Excluded Study | Estimated RD | pval | ci.lb | ci.ub | I2 | Qp |
| --- | --- | --- | --- | --- | --- | --- |
| Alvarez-Gordillo,1998 | -1.02 | 0.19 | -2.54 | 0.51 | 0.00 | 0.83 |
| Kamolratanakul,1999 | 0.20 | 0.87 | -2.21 | 2.61 | 53.45 | 0.08 |
| Okanurak,2007 | -0.10 | 0.94 | -2.64 | 2.45 | 55.91 | 0.06 |
| Walley,2001 | 0.40 | 0.73 | -1.84 | 2.63 | 44.64 | 0.13 |
| Zwarenstein,1998 | 0.16 | 0.89 | -2.07 | 2.38 | 54.18 | 0.08 |
| Zwarenstein,2000 | -0.17 | 0.89 | -2.44 | 2.11 | 56.48 | 0.06 |

###

### Cure

No studies were indicated as possibly influential outliers when measuring the RR or RD of cure (Table V and W). The effect of excluding each study on the estimated RR and RD are shown in . The estimated RR and RD are not significant when all of the studies are included and did not change when any single study was removed (Table X and Y).

Table V **RR Cure Outlier and Influence Analysis**

|  | Studentized Res | DFFITS | Cook’s Distance | Covariance Ratio | DFBETAS | Influence |
| --- | --- | --- | --- | --- | --- | --- |
| Alvarez-Gordillo,1998 | -1.96 | -0.83 | 0.36 | 0.73 | -0.80 | No |
| Kamolratanakul,1999 | -0.44 | -0.33 | 0.17 | 1.86 | -0.34 | No |
| Okanurak,2007 | 0.40 | 0.12 | 0.02 | 1.60 | 0.12 | No |
| Walley,2001 | 0.75 | 0.36 | 0.14 | 1.33 | 0.36 | No |
| Zwarenstein,1998 | 1.94 | 0.52 | 0.25 | 0.81 | 0.57 | No |
| Zwarenstein,2000 | -0.49 | -0.21 | 0.05 | 1.29 | -0.20 | No |

Table W **RD Cure Outlier and Influence Analysis**

|  | Studentized Res | DFFITS | Cook’s Distance | Covariance Ratio | DFBETAS | Influence |
| --- | --- | --- | --- | --- | --- | --- |
| Alvarez-Gordillo,1998 | -2.20 | -0.86 | 0.38 | 0.63 | -0.86 | No |
| Kamolratanakul,1999 | -0.49 | -0.27 | 0.09 | 1.58 | -0.27 | No |
| Okanurak,2007 | 0.36 | 0.14 | 0.03 | 1.57 | 0.14 | No |
| Walley,2001 | 0.58 | 0.25 | 0.07 | 1.44 | 0.25 | No |
| Zwarenstein,1998 | 1.86 | 0.66 | 0.35 | 0.79 | 0.70 | No |
| Zwarenstein,2000 | -0.33 | -0.16 | 0.03 | 1.41 | -0.15 | No |

Table X **RR Cure Sensitivity Analysis:** **pval** = P-value associated with estimate; **ci.lb** = Confidence interval lower bound; **ci.up** = Confidence interval upper bound; **I2** = Estimated heterogeneity; **Qp** = P-value associated with heterogeneity

| Excluded Study | Estimated RR | pval | ci.lb | ci.ub | I2 | Qp |
| --- | --- | --- | --- | --- | --- | --- |
| Alvarez-Gordillo,1998 | 0.97 | 0.64 | 0.87 | 1.09 | 47.13 | 0.06 |
| Kamolratanakul,1999 | 0.96 | 0.67 | 0.80 | 1.15 | 74.25 | 0.00 |
| Okanurak,2007 | 0.93 | 0.37 | 0.78 | 1.09 | 78.25 | 0.01 |
| Walley,2001 | 0.91 | 0.24 | 0.78 | 1.06 | 72.76 | 0.02 |
| Zwarenstein,1998 | 0.90 | 0.10 | 0.80 | 1.02 | 68.43 | 0.02 |
| Zwarenstein,2000 | 0.95 | 0.49 | 0.82 | 1.10 | 79.92 | 0.00 |

Table Y **RD Cure Sensitivity Analysis:** **pval** = P-value associated with estimate; **ci.lb** = Confidence interval lower bound; **ci.up** = Confidence interval upper bound; **I2** = Estimated heterogeneity; **Qp** = P-value associated with heterogeneity

| Excluded Study | Estimated RD | pval | ci.lb | ci.ub | I2 | Qp |
| --- | --- | --- | --- | --- | --- | --- |
| Alvarez-Gordillo,1998 | -1.10 | 0.76 | -8.31 | 6.11 | 62.00 | 0.03 |
| Kamolratanakul,1999 | -2.57 | 0.66 | -14.02 | 8.88 | 81.10 | 0.00 |
| Okanurak,2007 | -4.74 | 0.42 | -16.17 | 6.69 | 82.62 | 0.00 |
| Walley,2001 | -5.25 | 0.35 | -16.18 | 5.68 | 83.10 | 0.00 |
| Zwarenstein,1998 | -6.75 | 0.10 | -14.87 | 1.38 | 75.41 | 0.01 |
| Zwarenstein,2000 | -3.20 | 0.56 | -14.02 | 7.61 | 86.18 | 0.00 |

## Publication bias for each outcome

It is important to note that the power of any publication bias analysis is small with so few studies.

### Lost to follow-up (LTFU)


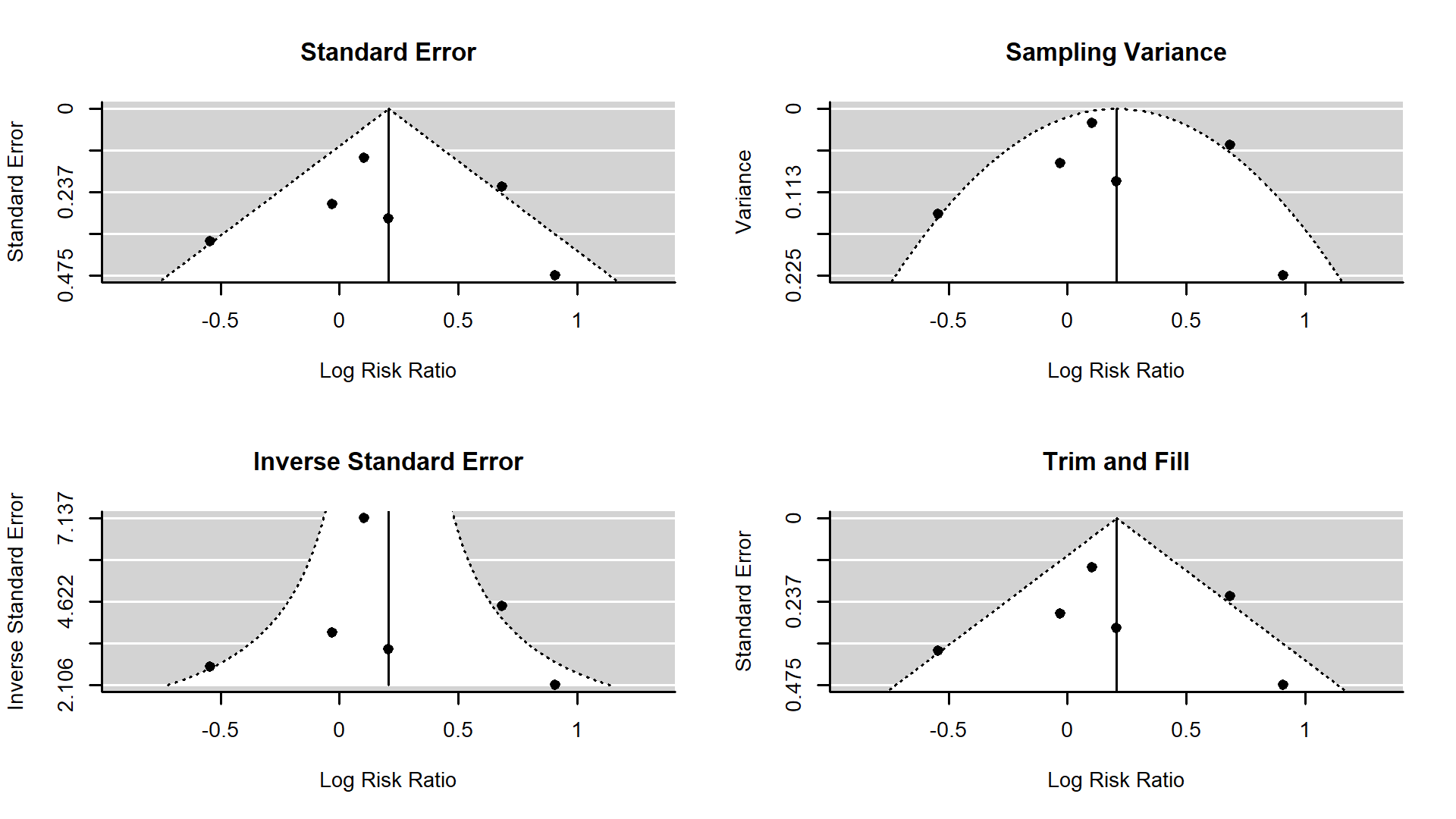
The different plots for assessing publication bias for the outcome of lost to follow-up are shown in Figure F and Figure G. None of the plots show any indication of publication bias. This is further supported by the trim and fill plot that does not show any need for additional studies to create symmetry.

Figure F RR Publication Bias
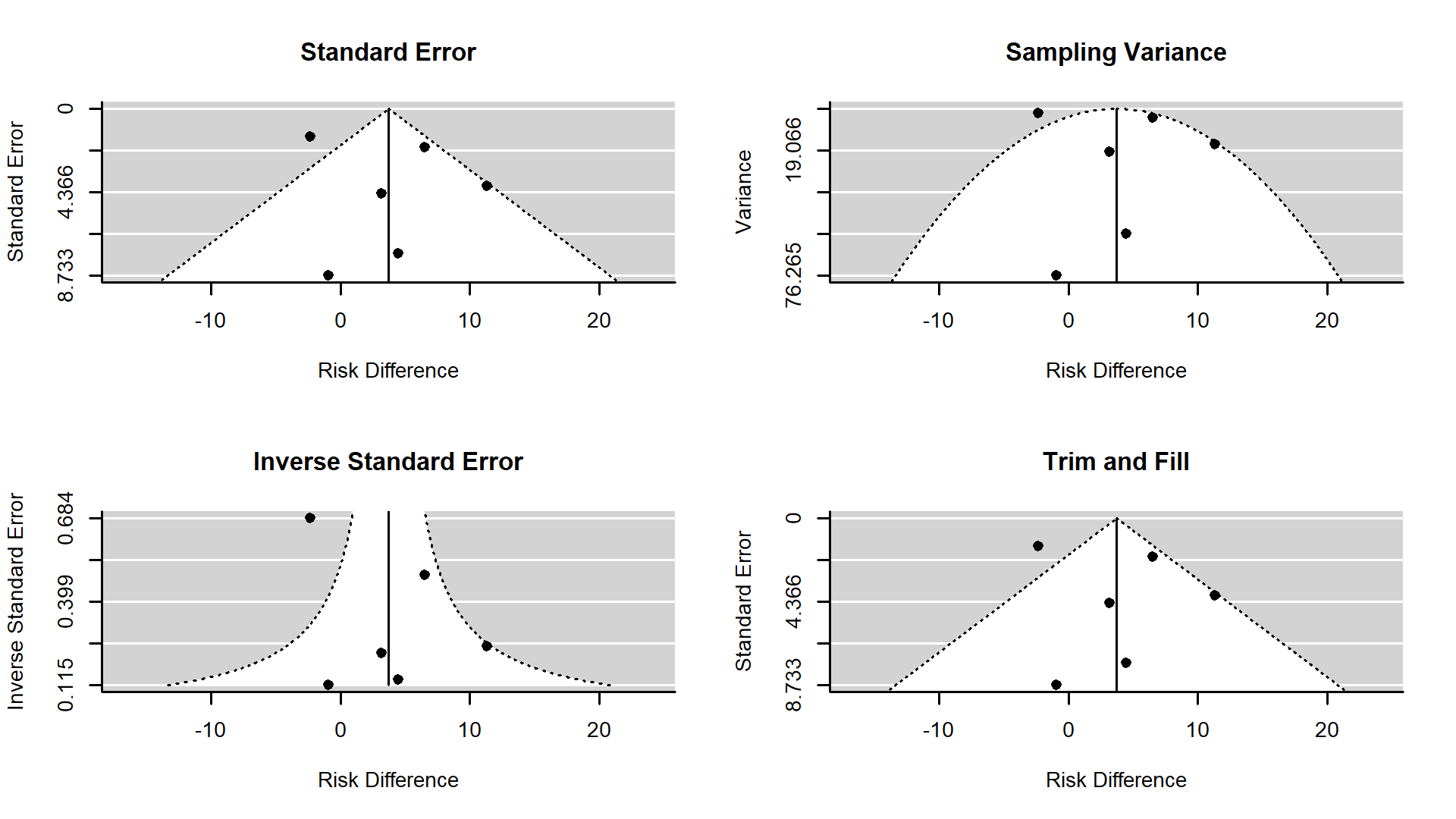


Figure G RD Publication Bias

### Treatment failure

The different plots for assessing publication bias for the outcome of treatment failure are shown in Figure H and Figure I. None of the plots show any indication of publication bias. This is further supported by the trim and fill plot that does not show any need for additional studies to create symmetry.


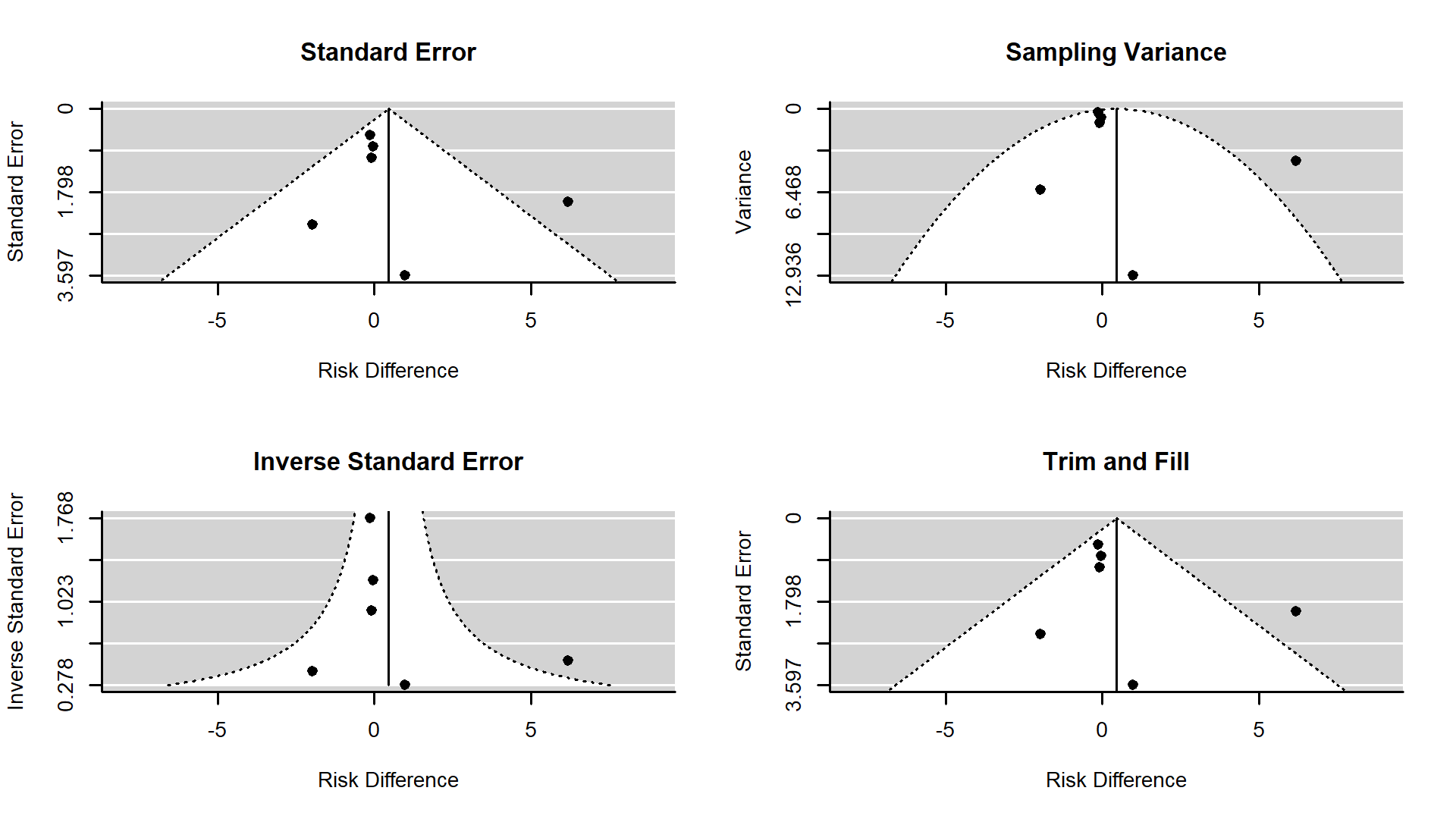


Figure H RR Publication Bias


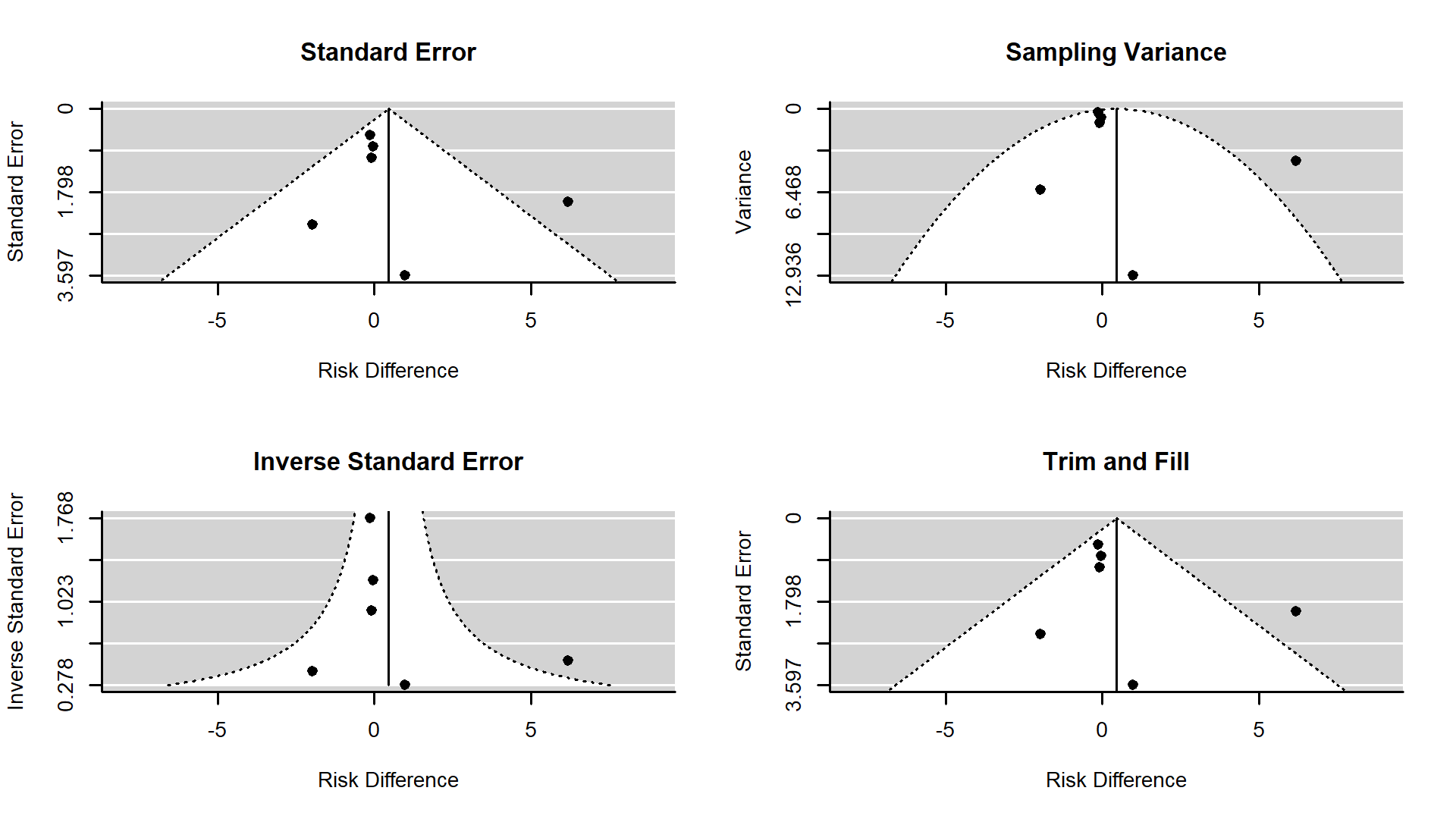


Figure I RD Publication Bias

### Completing treatment

The different plots for assessing publication bias for the outcome of completing treatment are shown in Figure J and Figure K. None of the plots show any indication of publication bias. This is further supported by the trim and fill plot that does not show any need for additional studies to create symmetry.


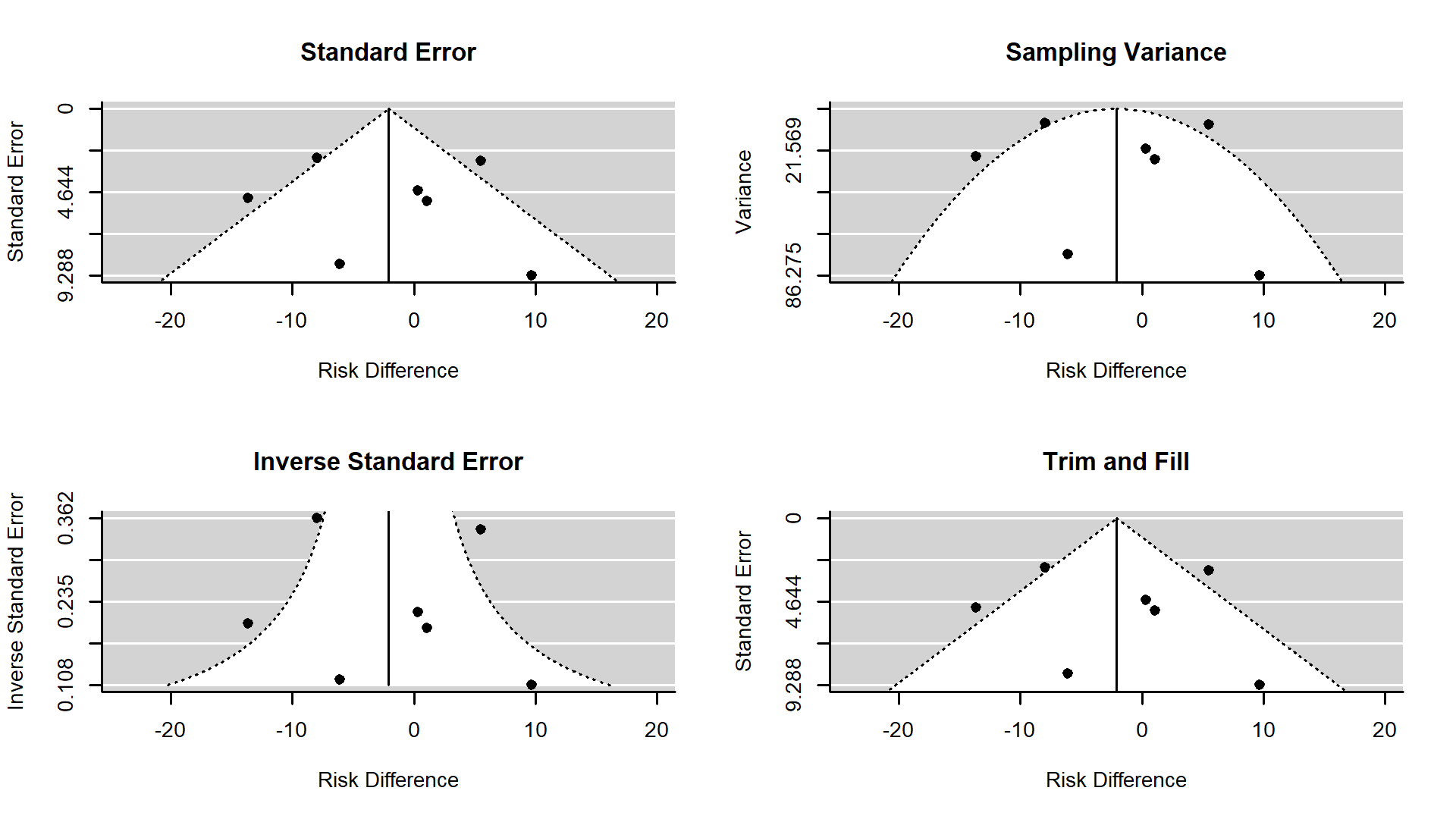


Figure J RR Publication Bias


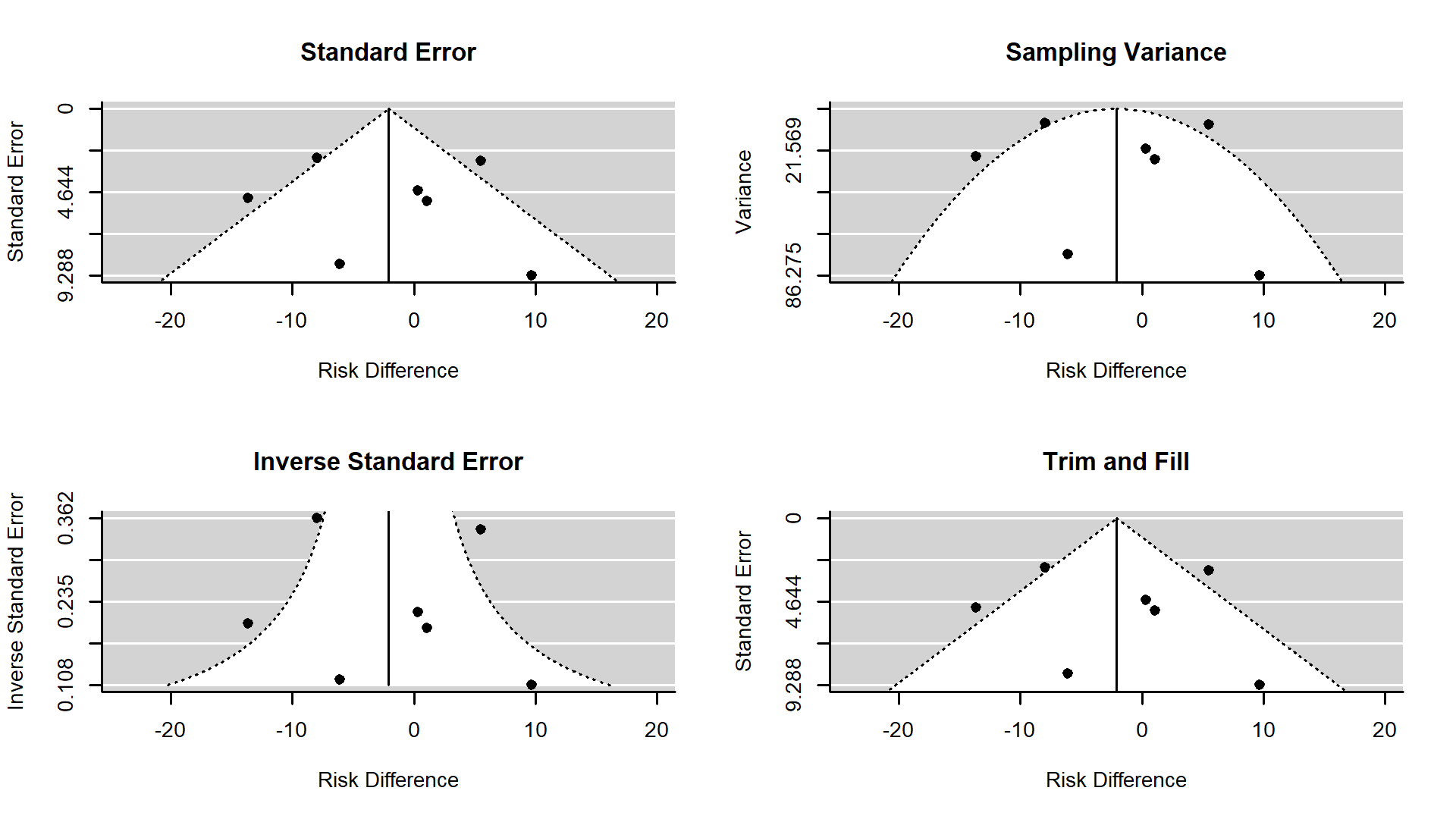


Figure K RD Publication Bias

### Death

The different plots for assessing publication bias for the outcome of death are shown in Figure L and Figure M. None of the plots show any indication of publication bias. This is further supported by the trim and fill plot that does not show any need for additional studies to create symmetry.


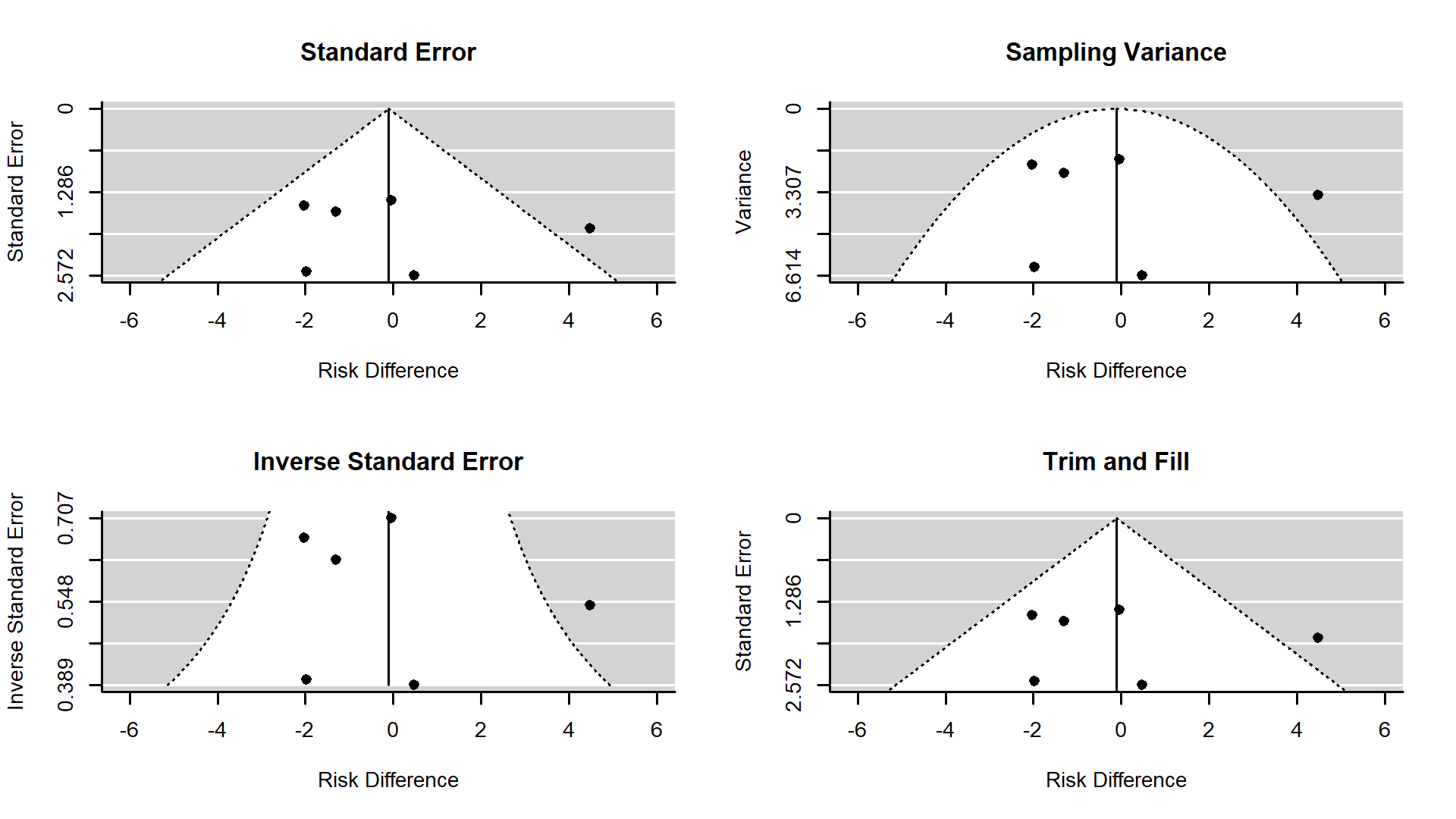


Figure L RR Publication Bias


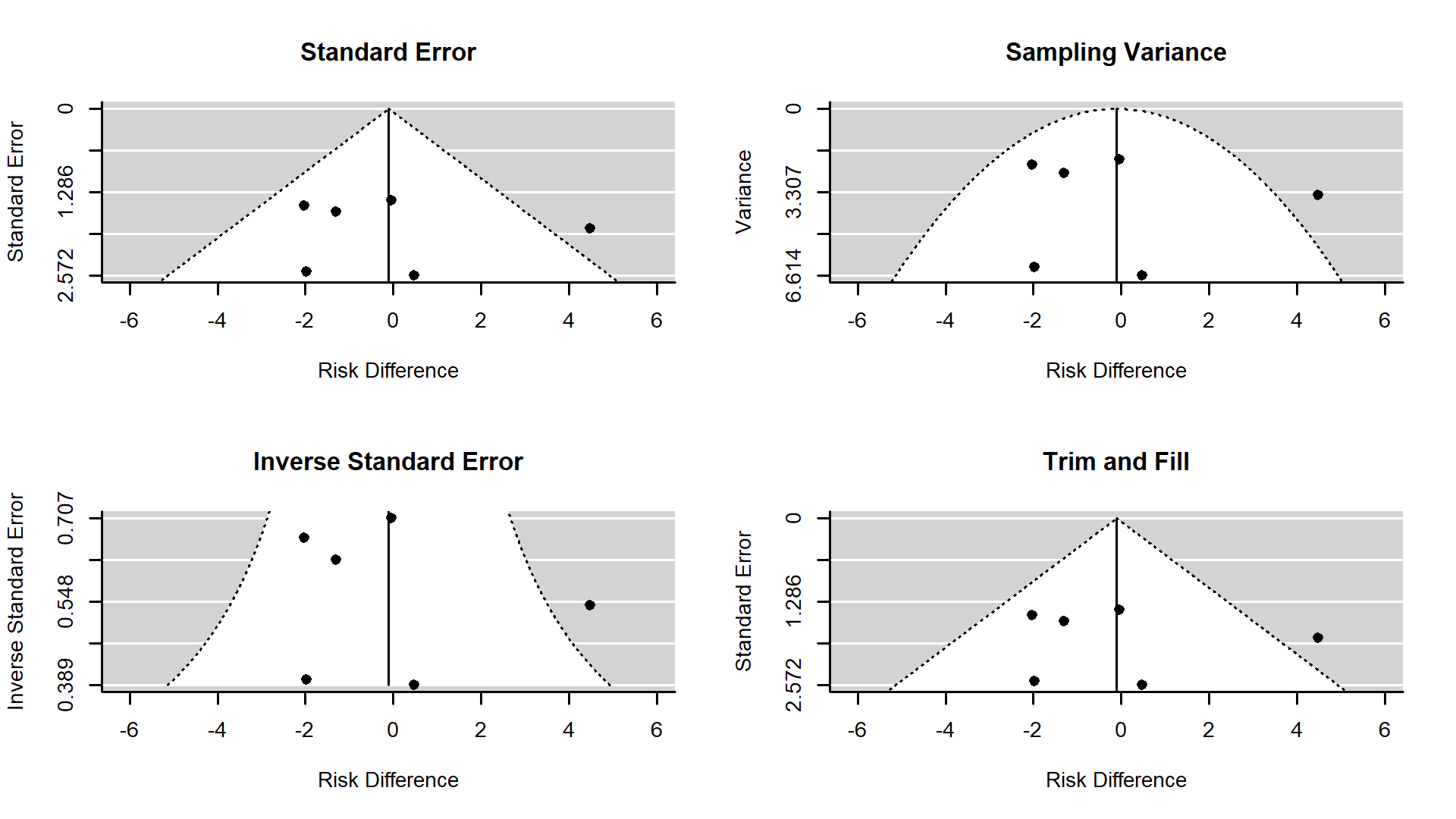


Figure M RD Publication Bias

### Cure

The different plots for assessing publication bias for the outcome of cure are shown in Figure N and Figure O. None of the plots show any indication of publication bias. This is further supported by the trim and fill plot that does not show any need for additional studies to create symmetry.


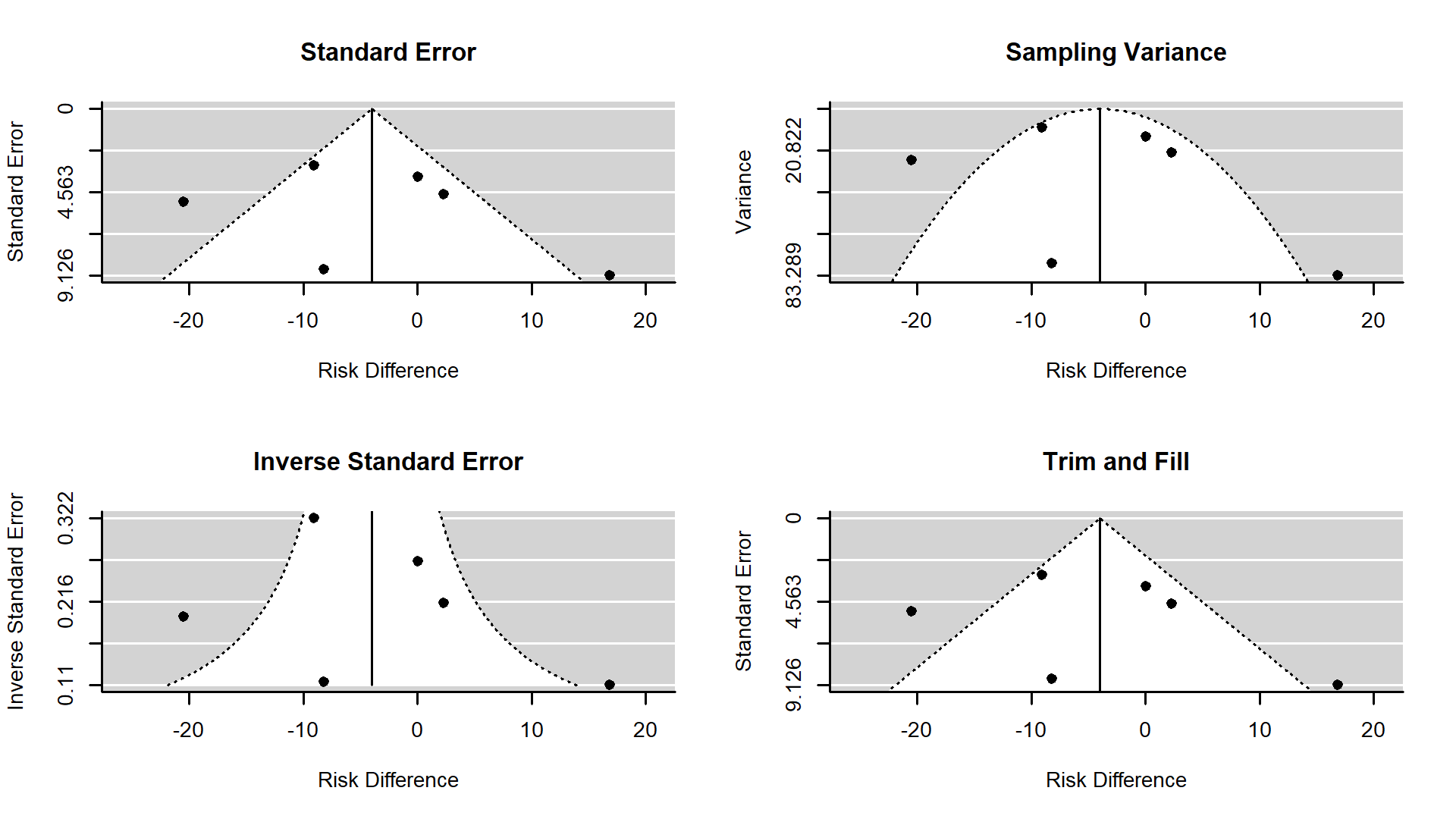


Figure N RR Publication Bias
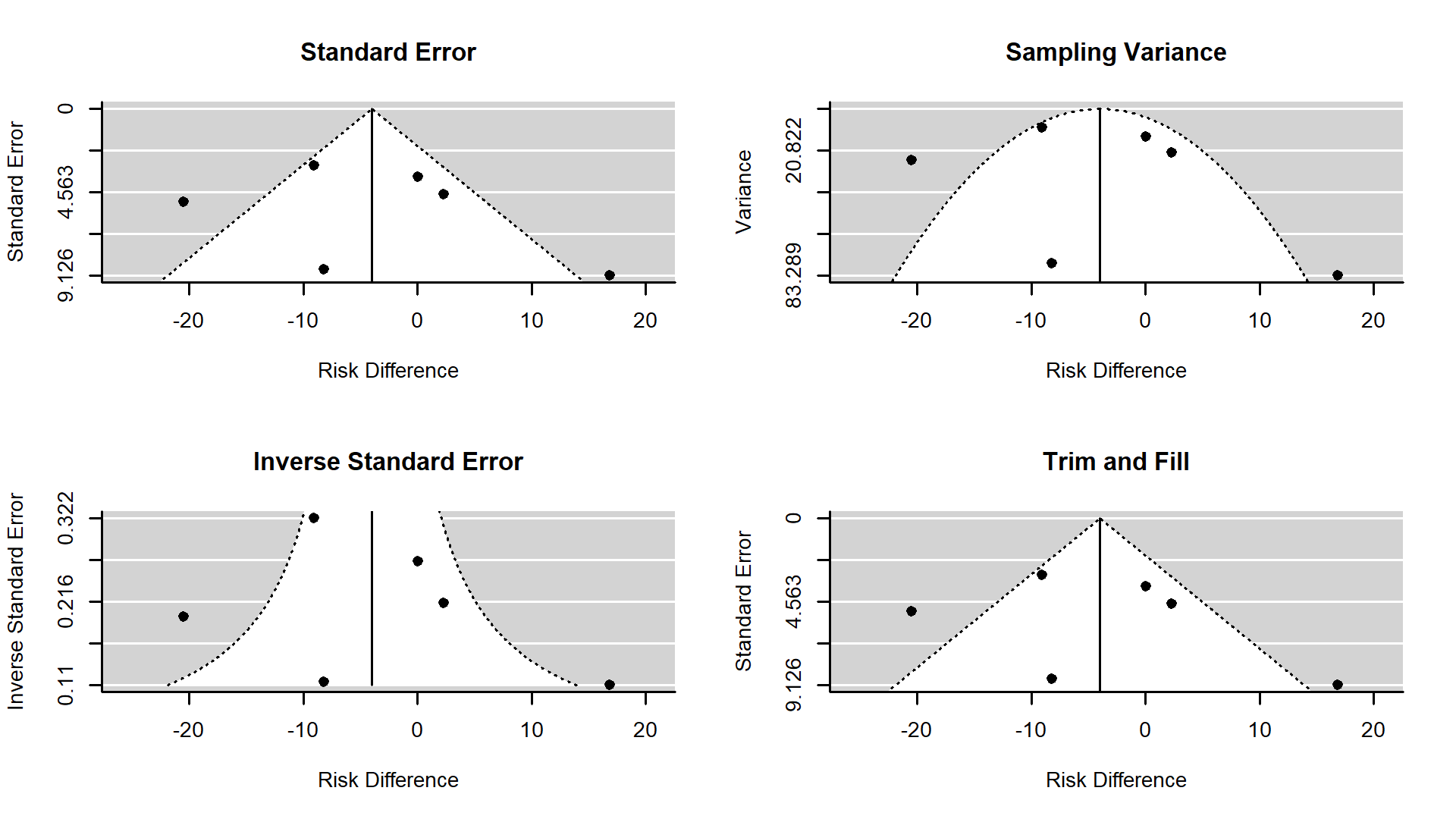


Figure O RD Publication Bias

# Reproducing the results presented in PG

## Comparision of included studies

The following paragraphs highlight the differences in study inclusion between PG and our systematic literature review.

PG states that intention to treat (ITT) data was used in their analysis. However, investigating their reported numbers suggests that they used the *as-treated* data for the one study which reported data both *as-treated* and ITT ^(6)^. We recorded data as both ITT and *as-treated* when studies differentiated between the two. When available, ITT data was used, as this is a better reflection of the efficiency of the program in real-world situations.

Two studies ^(7,8)^ included re-treatment patients, which violated PG’s inclusion criteria. The authors nevertheless included them in their analysis. Since in these studies, treatment was randomized without regard to their to re-treatment status, we included these studies in our analysis. Our inclusion criteria are thus slightly different than PG.

Several studies included in PG’s meta-analysis are not included in ours. According to PG’s inclusion and exclusion criteria, the studies described should have been excluded by PG. The following studies were excluded.

In (9), the SAT group does not match the definitions provided in PG. The purpose of this study is to compare long and short drug therapy combinations, not to distinguish differences between strategies for treatment administration ^(9)^. We excluded this study from our analysis. Others had previously criticized the inclusion of this study in PG ^(10)^.

In (11), some individuals had previously received treatment for TB. It also included people who had extra-pulmonary TB disease and had a biased assignment to DOT for patients with a high risk of being lost to follow-up. According to the exclusion criteria listed in PG, this study should not have been included in their analysis.

In (12) the data was not strictly collected prospectively; instead, that study contained a combination of retrospective data from 1988-1999 and prospective from 1999-2000. The study collected less than 1% of the data prospectively, and it included re-treatment cases with a biased assignment to DOT. Since this study included re-treatment cases and used retrospective data, it should not have been included in PG according to their inclusion criteria.

We did not include relapse and ADR as outcomes because only three studies provided data and none met the inclusion criteria. Two of the studies were not adequately designed ^(11,12)^ to assess the impact of DOT on relapse or ADR, and the third study ^(9)^ lacked sufficient SAT and DOT groups.

In (13) the cohort included children and a large proportion of HIV-positive individuals. Also, the data reported and used by PG are outcomes at two months not the end of treatment.

In ^(6)^ the cohort included children and a large proportion of HIV-positive individuals. The study also reported an extreme lack of allocation adherence. The study poorly defined how individuals were allocated. The Authors of the Cochrane review had previously rejected it based on allocation concerns ^(14)^. PG claims it used ITT analysis, but the data reported by PG is “as treated” for this study. We chose to exclude it after we were unable to obtain the data from the author.

There were two studies we found that did compare DOT and SAT but were excluded for the following reasons. In (15) the cohort included children and extra-pulmonary TB. We excluded this study from our analysis. The study by (16) was very poorly reported and could not be adequately assessed for quality. Due to quality concerns, we excluded this study from our analysis. As stated in the main text we tried to obtain additional data from a number of studies, but we were not successful.

## Comparison of data re-abstraction

We re-abstracted the data for all the studies included in PG and there are a number of inconsistencies. The results are detailed in “Systematic Literature Review DOT vs SAT” file sheet 15 named “Comparison of Repeated Data Abstraction of PG”. The data used to reproduce the results below are based on what is reported in the published study not our re-abstracted data.

## Reproducing figures and tables from PG

To reproduce the results reported by PG for lost to follow-up required a certain level of experimentation. In PG the term “default” is used instead of “lost to follow-up”.

### Figure 2 from PG

Figure 2 in PG shows a risk difference of -0.07 (95% CI,-0.08, -0.05) in PG’s text the risk difference is reported to be -0.05 (95% CI, -0.07,-0.04). We report percent risk difference but we will use the same reference group as PG.

To try and reproduce those results, the data used for this analysis were abstracted directly from the 2013 publication ^(1)^. We started with the statistical methods described in PG, namely a meta-analysis using a DerSimonian and Laird mixed effects model with modulators (Figure P).

Comparing our results there are a number of difference. The overall result is not significant, and while the point estimate is close to those from figure 2 in PG, they are not close to the estimate reported in the text of PG. Our confidence intervals are not close to those reported in PG. The overall effect measure in the RTC is not similar, but the one for the PCS subgroup is nearly identical. Additionally, the weights used are very different in some cases.

The statistical method that produced results the closest to those presented in figure 2 of PG was a fixed effect model. Using the fixed effects model generated nearly identical measures of effect and weighting as those reported in figure 2 of PG (Figure Q).


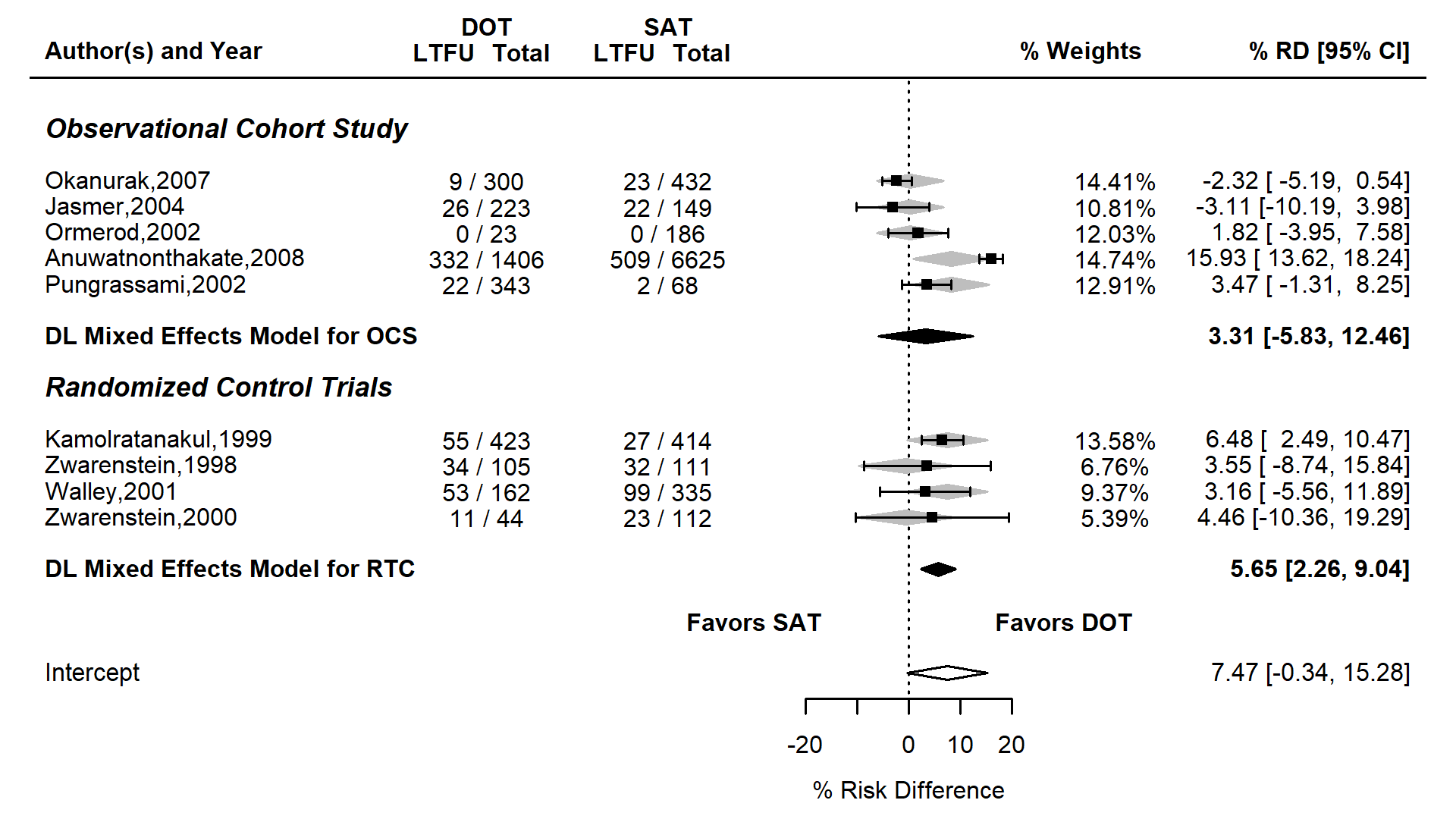


Figure P Lost to Follow-up Using Methods From PG


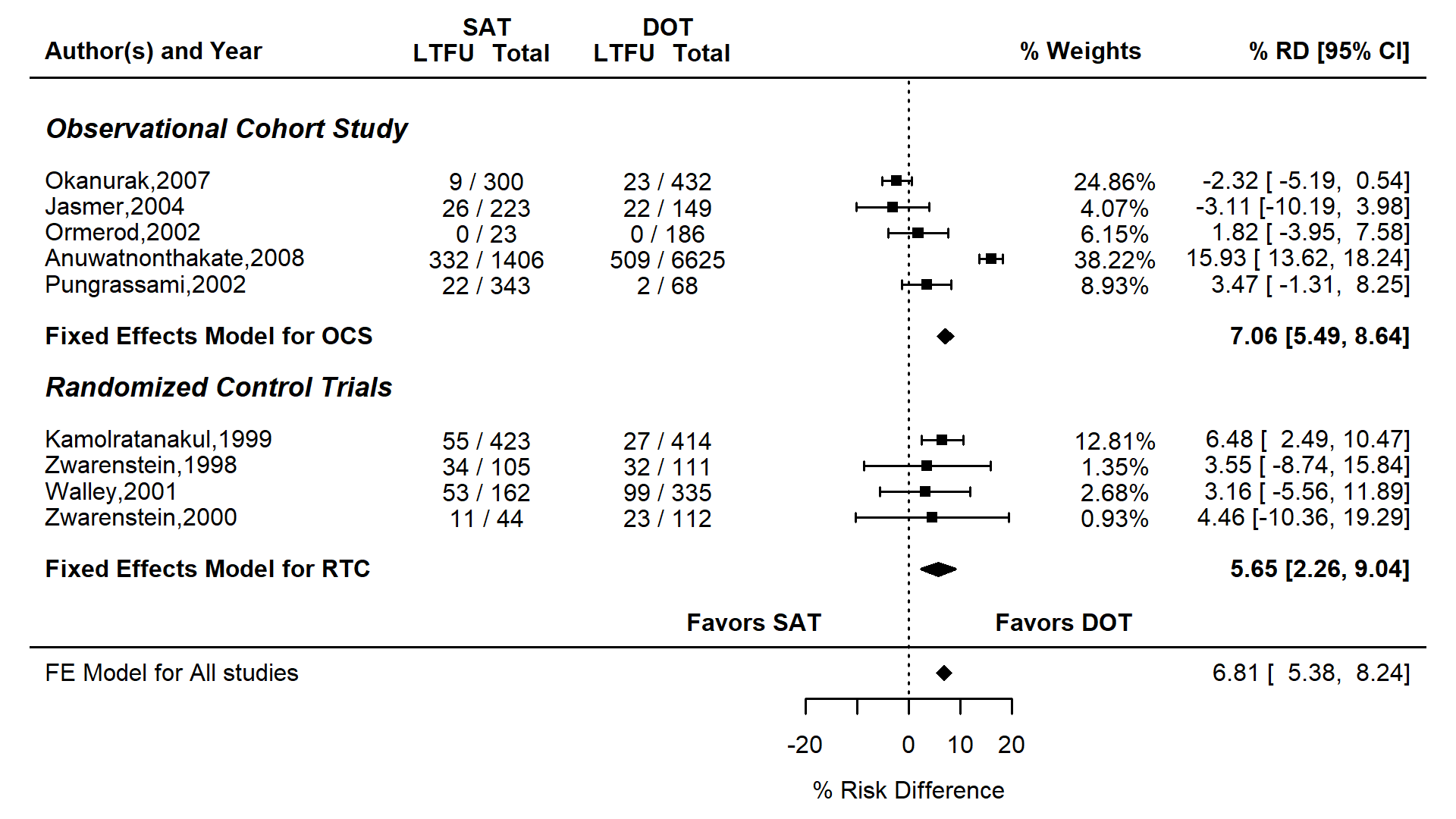


Figure Q Lost to Follow-up Using Fixed Effects

### Table 2 from PG

There was little information regarding the incidence rates in Table 2 of PG. It seems that they calculated the proportion or risk of being lost to follow up, not the incidence rate. We used the data as reported in PG to generate the simple table below. Most of the point estimates lineup but others do not. It is possible that different numbers were used in those cases, or that recording errors occurred. While we tried several approaches, we could not reproduce table 2. Our attempt to reproduce table 2 are shown in Table Z.

Table Z **Reproduced PG Table 2**

|  | Risk% DOT | 95%CI LB | 95%CI UB | Risk% SAT | 95%CI LB | 95%CI UB |
| --- | --- | --- | --- | --- | --- | --- |
| Kamolratanakul | 6.52 | 4.42 | 9.46 | 13.00 | 10.02 | 16.67 |
| Zwarenstein | 28.83 | 20.82 | 38.32 | 32.38 | 23.76 | 42.31 |
| Walley | 29.55 | 24.78 | 34.80 | 32.72 | 25.68 | 40.59 |
| Zwarenstein | 20.54 | 13.72 | 29.42 | 25.00 | 13.70 | 40.65 |
| Okanurak | 5.32 | 3.48 | 8.00 | 3.00 | 1.47 | 5.81 |
| Jasmer | 14.77 | 9.68 | 21.72 | 11.66 | 7.90 | 16.79 |
| Ormerod | 0.00 | 0.00 | 2.52 | 0.00 | 0.00 | 17.81 |
| Anuwatnonthakate | 7.68 | 7.06 | 8.36 | 23.61 | 21.43 | 25.94 |
| Pungrassami | 2.94 | 0.51 | 11.16 | 6.41 | 4.16 | 9.69 |

###

### Figure 3 from PG

Figure 3 in PG showed the estimated risk difference for treatment failure. The data used is directly from PG. We first tried to reproduce the figure and results using the methods described. These results are very close to the ones presented in the original only small differences in the weight of each study. Our reproduced results are shown in Figure R.


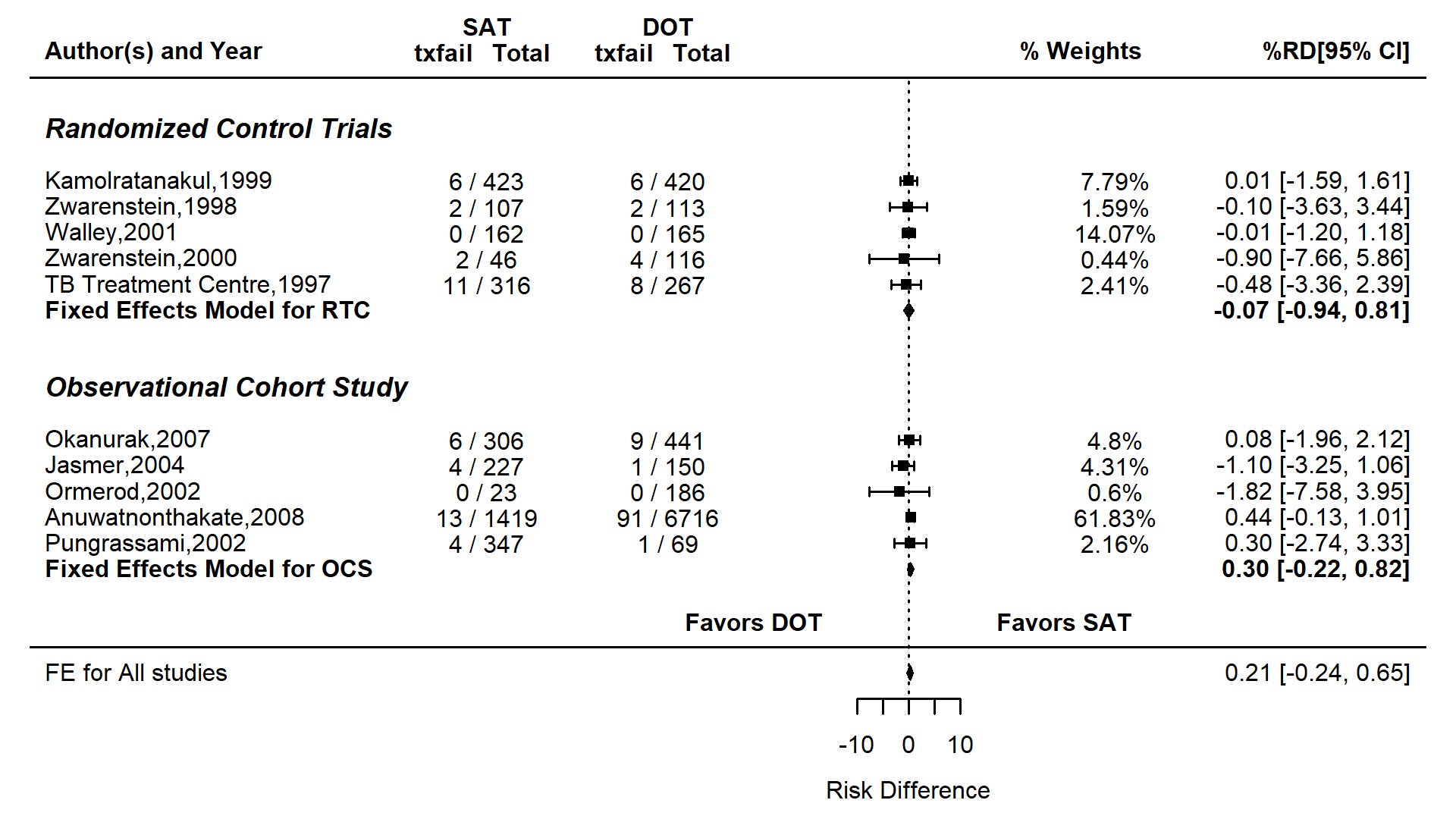


Figure R Fixed Effects Treatment Failure

### Table 3 from PG

We were able to reproduce part of table 3. We did not attempt to reproduce table 3 results regarding relapse or acquired drug resistance. The results are very similar to those presented in the original text any differences are likely due to rounding. Table AA shows our results for treatment failure using data from PG.

Table AA **Reproduced PG Table 3**

|  | Risk% DOT | 95%CI LB | 95%CI UB | Risk% SAT | 95%CI LB | 95%CI UB |
| --- | --- | --- | --- | --- | --- | --- |
| Kamolratanakul | 1.43 | 0.58 | 3.24 | 1.42 | 0.58 | 3.22 |
| Zwarenstein | 1.77 | 0.31 | 6.88 | 1.87 | 0.32 | 7.25 |
| Walley | 0.30 | 0.00 | 3.35 | 0.31 | 0.00 | 3.41 |
| Zwarenstein | 3.45 | 1.11 | 9.12 | 4.35 | 0.76 | 16.04 |
| TB Treatment Centre | 3.00 | 1.40 | 6.04 | 3.48 | 1.84 | 6.32 |
| Okanurak | 2.04 | 1.00 | 3.98 | 1.96 | 0.80 | 4.43 |
| Jasmer | 0.67 | 0.03 | 4.22 | 1.76 | 0.57 | 4.75 |
| Ormerod | 0.27 | 0.00 | 2.98 | 2.17 | 0.00 | 20.99 |
| Anuwatnonthakate | 1.35 | 1.10 | 1.67 | 0.92 | 0.51 | 1.61 |
| Pungrassami | 1.45 | 0.08 | 8.89 | 1.15 | 0.37 | 3.13 |

# Reproducibility of meta-analyses

Meta-analysis is a valuable tool and provides an authoritative summary of available data, but currently, they are not easily reproducible. While the PRISMA guideline does provide a systematic framework to create and assess the quality of meta-analyses, it does not ensure the reproducibility ^(17)^. The ability to reproduce results is a cornerstone of the scientific method. The need for reproducibility is increasingly realized by many journals and funding agencies which now require all the necessary scripts and data used to generate the results ^(18)^. Careful documentation that allows reproducibility is especially crucial for meta-analyses, which are meant to influence clinical and public health practice directly.

# References

1. Pasipanodya JG, Gumbo T. A meta-analysis of self-administered vs directly observed therapy effect on microbiologic failure, relapse, and acquired drug resistance in tuberculosis patients (vol 57, pg 21, 2013). Clinical Infectious Diseases. 2013;57(8):1223–3.

2. Viechtbauer W, Cheung MW-L. Outlier and influence diagnostics for meta-analysis. Research synthesis methods. 2010;1(2):112–25.

3. Kamolratanakul P, Sawert H, Lertmaharit S, Kasetjaroen Y, Akksilp S, Tulaporn C, et al. Randomized controlled trial of directly observed treatment (dot) for patients with pulmonary tuberculosis in thailand. Transactions of the Royal Society of Tropical Medicine and Hygiene. 1999;93(5):552–7.

4. Okanurak K, Kitayaporn D, Wanarangsikul W, Koompong C. Effectiveness of dot for tuberculosis treatment outcomes: A prospective cohort study in bangkok, thailand. The International Journal Of Tuberculosis And Lung Disease. 2007;11(7):762–8.

5. Del Carmen Alvarez-Gordillo G, Dorantes-Jimenez J. Shortened directly observed treatment applied to the tuberculosis control program. Salud Publica de Mexico. 1998;40(3):272–5.

6. Pungrassami P, Johnsen SP, Chongsuvivatwong V, Olsen J. Has directly observed treatment improved outcomes for patients with tuberculosis in southern thailand? Tropical Medicine & International Health. 2002;7(3):271–9.

7. Zwarenstein M, Schoeman JH, Vundule C, Lombard CJ, Tatley M. Randomised controlled trial of self-supervised and directly observed treatment of tuberculosis. The Lancet. 1998;352(9137):1340–3.

8. Zwarenstein M, Schoeman JH, Vundule C, Lombard CJ, Tatley M. A randomised controlled trial of lay health workers as direct observers for treatment of tuberculosis. The International Journal of Tuberculosis and Lung Disease. 2000;4(6):550–4.

9. Tuberculosis Research C. A controlled clinical trial of oral short-course regimens in the treatment of sputum-positive pulmonary tuberculosis. The International Journal of Tuberculosis and Lung Disease. 1997;1(6):509–17.

10. Nunn AJ, Phillips PPJ. An analysis with serious flaws. Clinical Infectious Diseases. 2013;57(7):1064–5.

11. Jasmer RM, Seaman CB, Gonzalez LC, Kawamura LM, Osmond DH, Daley CL. Tuberculosis treatment outcomes: Directly observed therapy compared with self-administered therapy. American journal of respiratory and critical care medicine. 2004;170(5):561–6.

12. Ormerod L, Horsfield N, Green R, others. Tuberculosis treatment outcome monitoring: Blackburn 1988–2000. The International Journal of Tuberculosis and Lung Disease. 2002;6(8):662–5.

13. Anuwatnonthakate A, Limsomboon P, Nateniyom S, Wattanaamornkiat W, Komsakorn S, Moolphate S, et al. Directly observed therapy and improved tuberculosis treatment outcomes in thailand. PLoS One. 2008;3(8):e3089.

14. Volmink J, Garner P. Directly observed therapy for treating tuberculosis. Cochrane Database Syst Rev. 2007;4.

15. Mathema B, Pande S, Jochem K, Houston R, Smith I, Bam D, et al. Tuberculosis treatment in nepal: A rapid assessment of government centers using different types of patient supervision. The International Journal of Tuberculosis and Lung Disease. 2001;5(10):912–9.

16. Tandon M, Gupta M, Tandon S, Gupta KB. DOTS versus self administered therapy (sat) for patients of pulmonary tuberculosis: A randomised trial at a tertiary care hospital. Indian journal of medical sciences. 2002;56(1):19–21.

17. Moher D, Liberati A, Tetzlaff J, Altman DG. Preferred reporting items for systematic reviews and meta-analyses: The prisma statement. Journal of Clinical Epidemiology. 2009;62(10):1006–12.

18. Collins FS, Tabak LA. NIH plans to enhance reproducibility. Nature. 2014;505(7485):612.
