# Supplementary figures and images for: An attempt to reproduce a previous meta-analysis and a new analysis regarding the impact of directly observed therapy on tuberculosis treatment outcomes
Source: PLoS One. 2019 May 23;14(5):e0217219. doi: 10.1371/journal.pone.0217219 (PMC6532908; doi:10.1371/journal.pone.0217219)

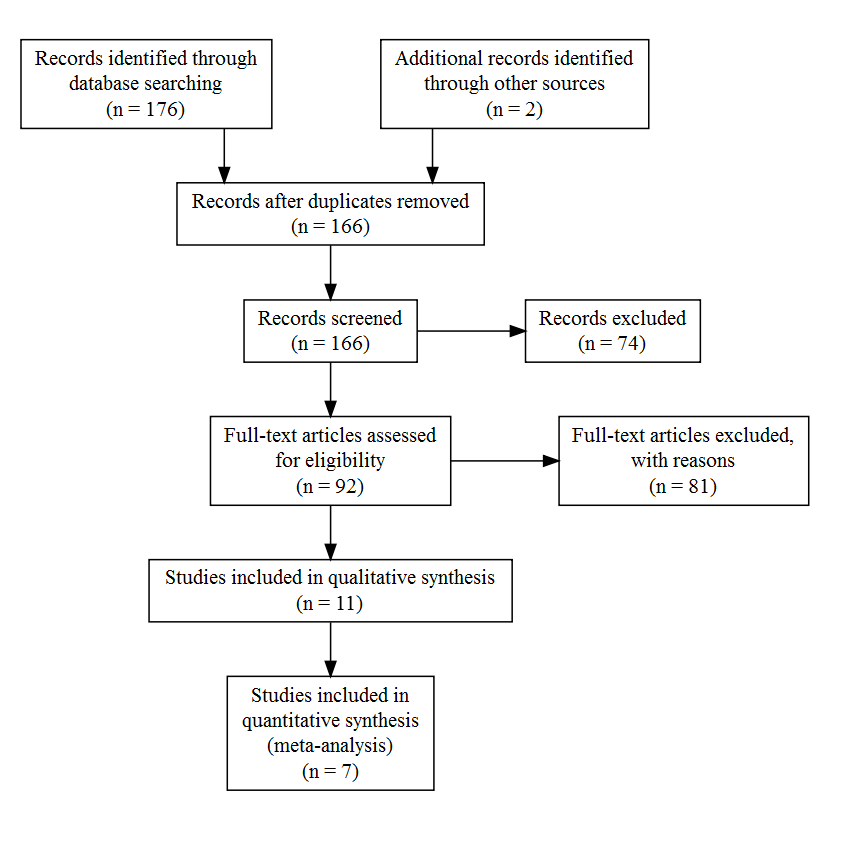

Supplement: S1 Folder — This folder contains all of the files described in the Supplementary Documentation. (ZIP) [file pone.0217219.s002.zip › Figures/Fig 1.tif]

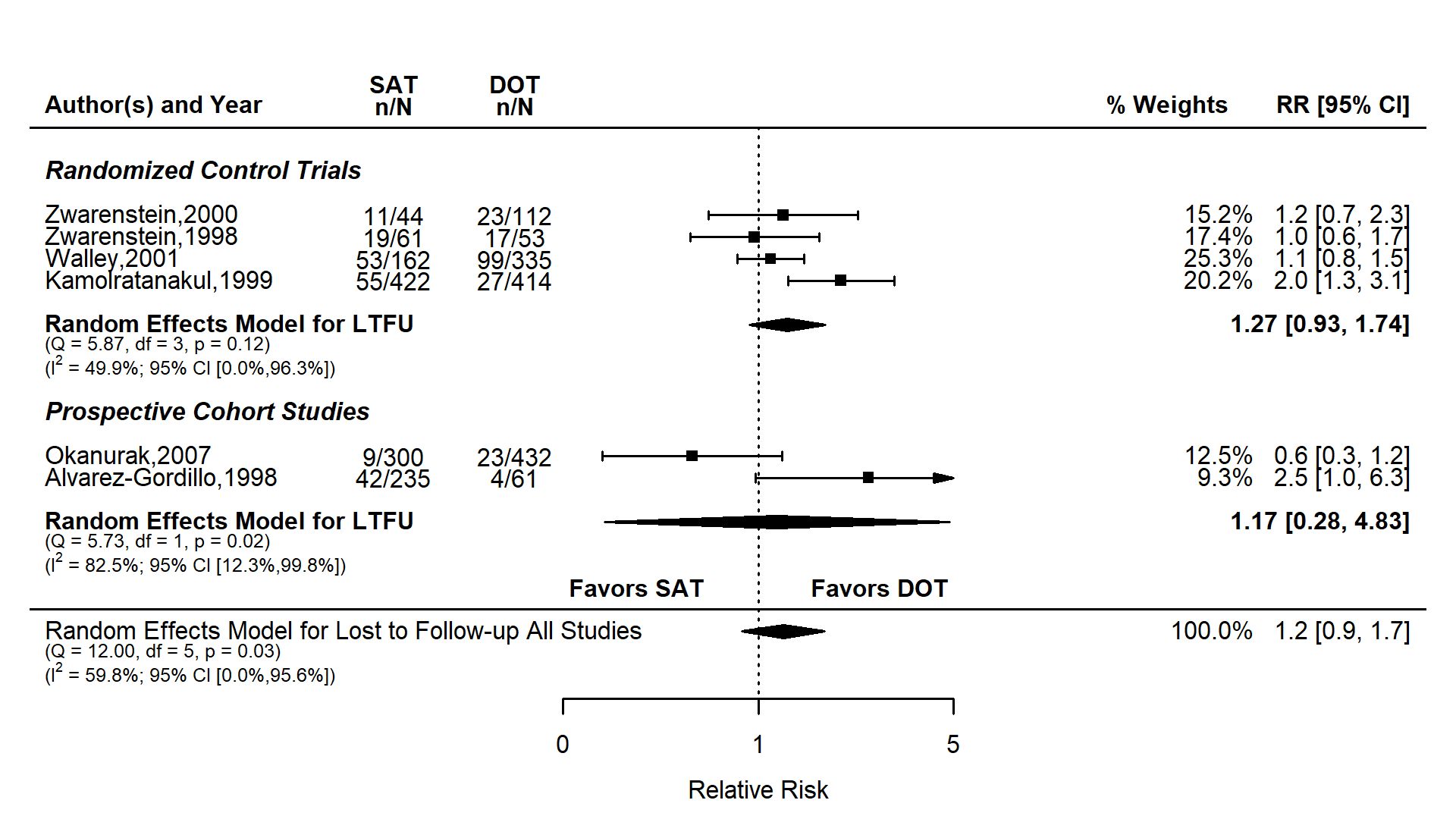

Supplement: S1 Folder — This folder contains all of the files described in the Supplementary Documentation. (ZIP) [file pone.0217219.s002.zip › Figures/Fig 2.tif]

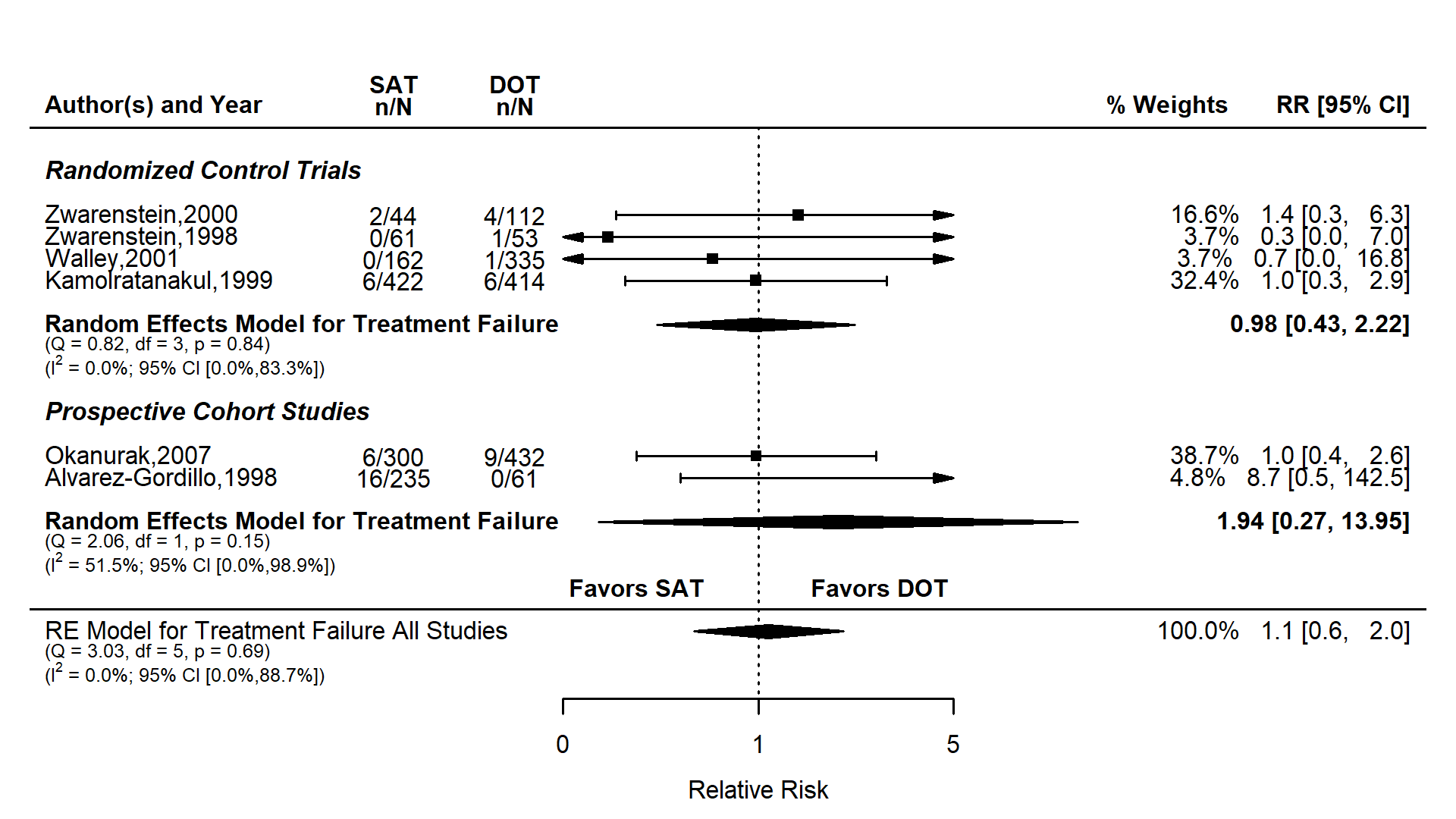

Supplement: S1 Folder — This folder contains all of the files described in the Supplementary Documentation. (ZIP) [file pone.0217219.s002.zip › Figures/Fig 3.tif]

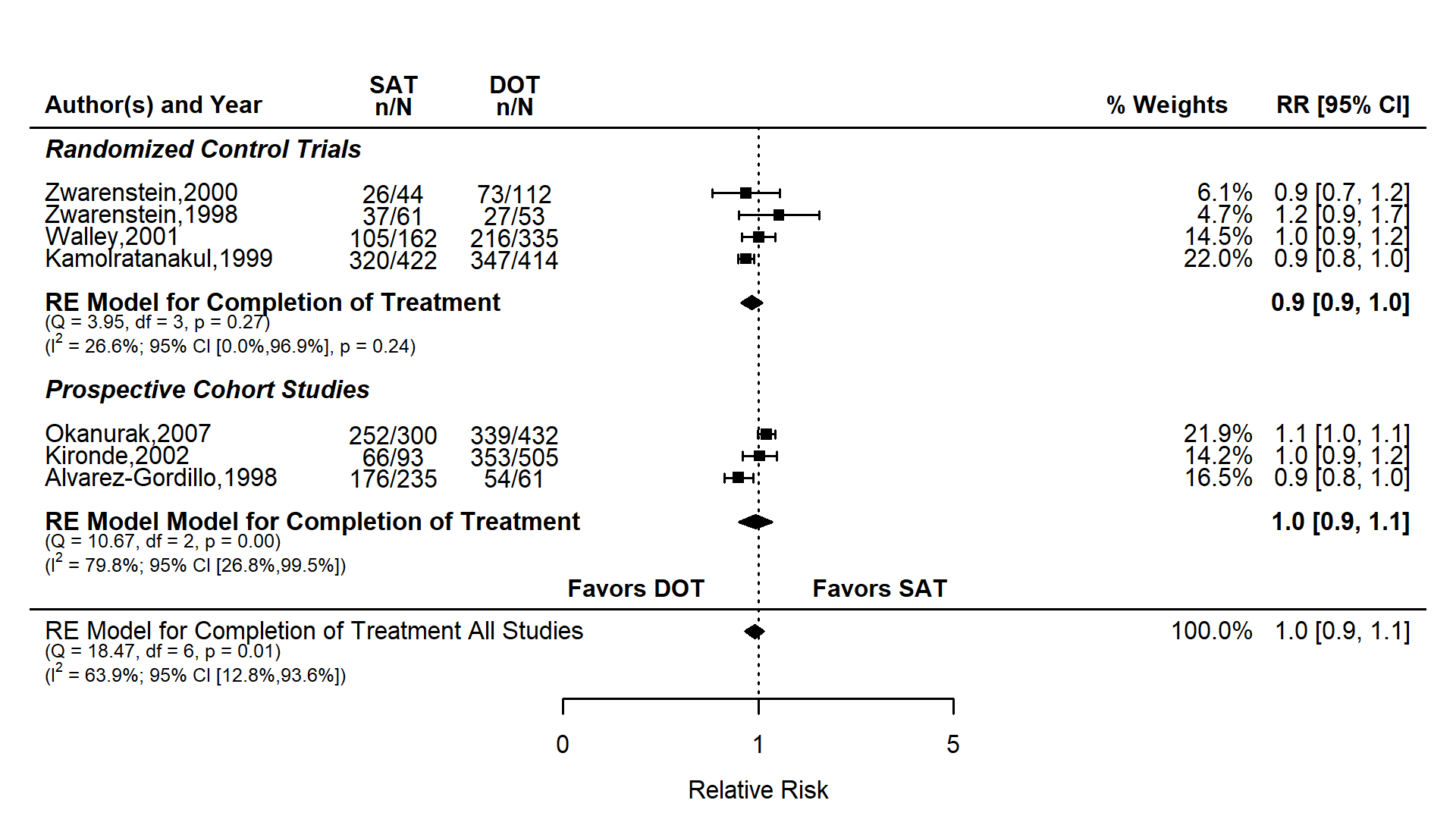

Supplement: S1 Folder — This folder contains all of the files described in the Supplementary Documentation. (ZIP) [file pone.0217219.s002.zip › Figures/Fig 4.tif]

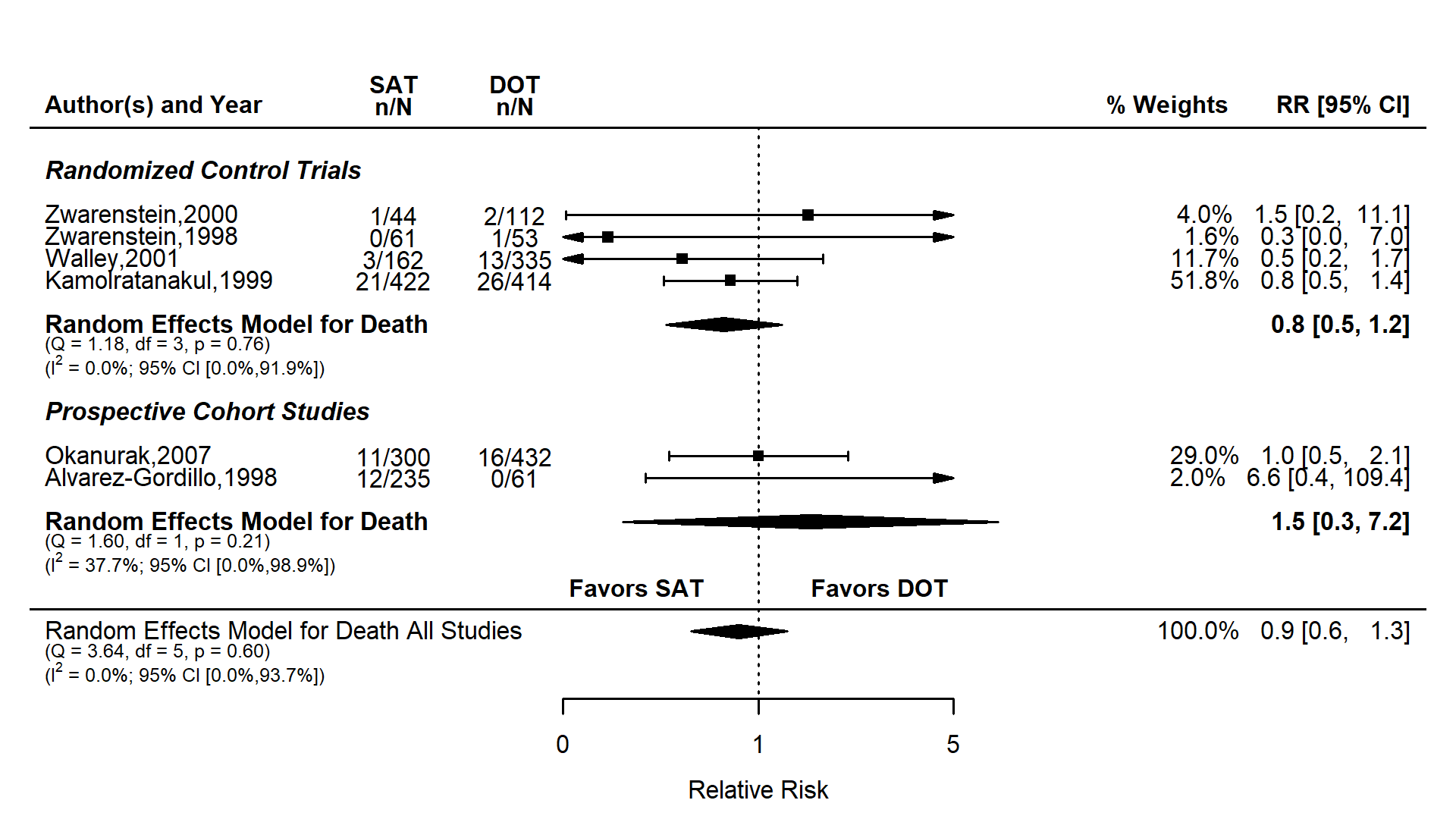

Supplement: S1 Folder — This folder contains all of the files described in the Supplementary Documentation. (ZIP) [file pone.0217219.s002.zip › Figures/Fig 5.tif]

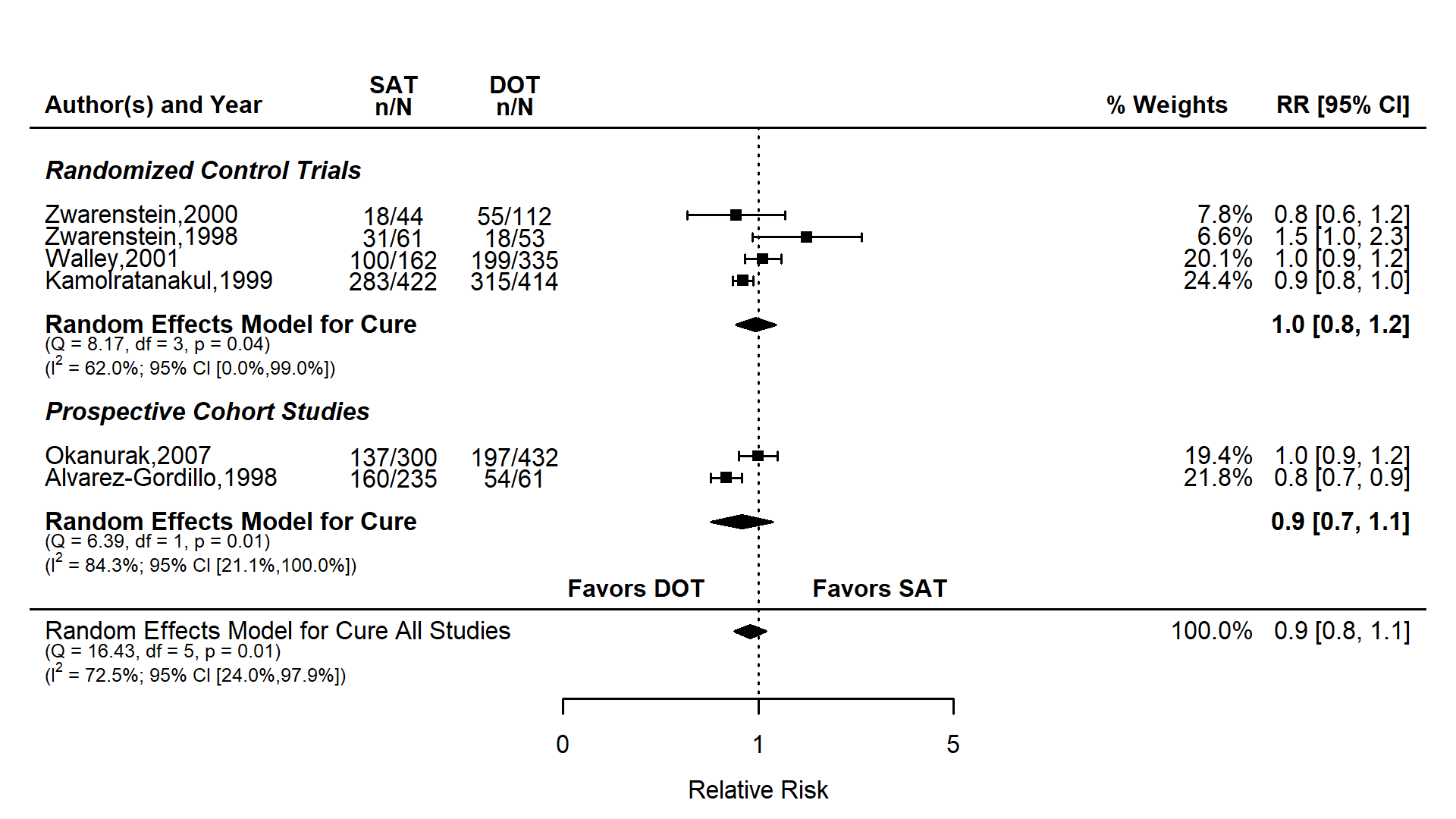

Supplement: S1 Folder — This folder contains all of the files described in the Supplementary Documentation. (ZIP) [file pone.0217219.s002.zip › Figures/Fig 6.tif]

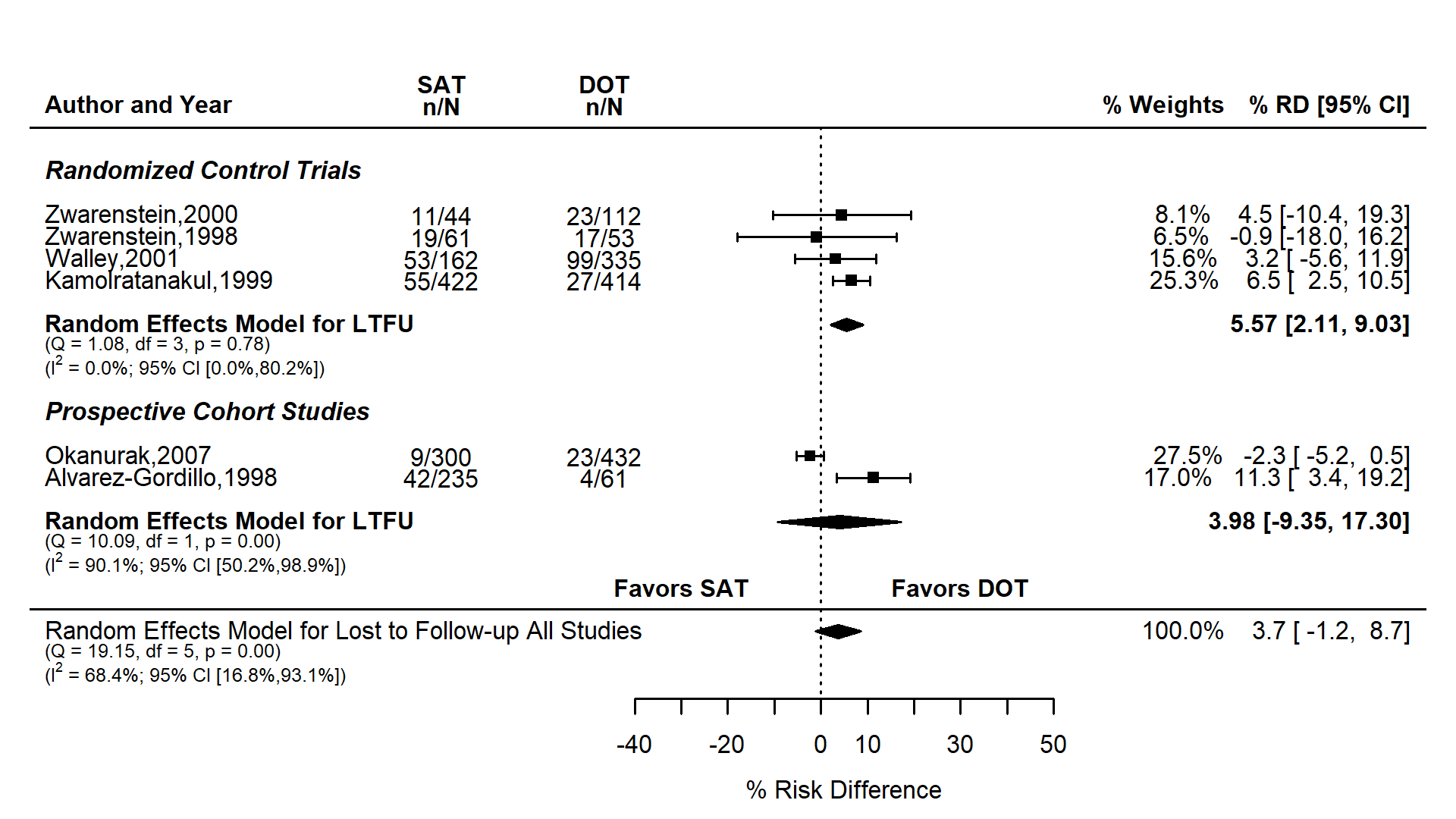

Supplement: S1 Folder — This folder contains all of the files described in the Supplementary Documentation. (ZIP) [file pone.0217219.s002.zip › Figures/Figure A.tif]

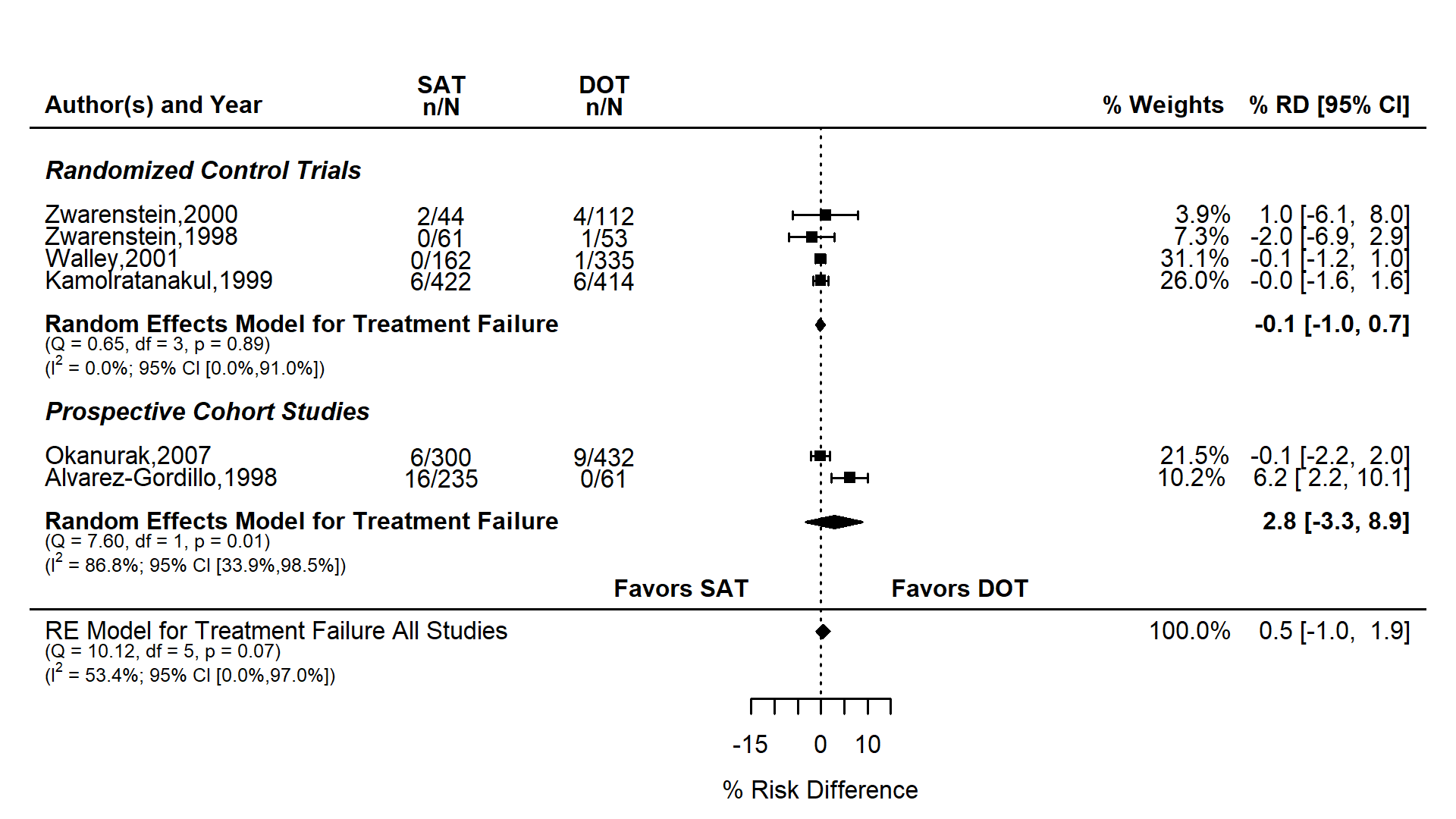

Supplement: S1 Folder — This folder contains all of the files described in the Supplementary Documentation. (ZIP) [file pone.0217219.s002.zip › Figures/Figure B.tif]

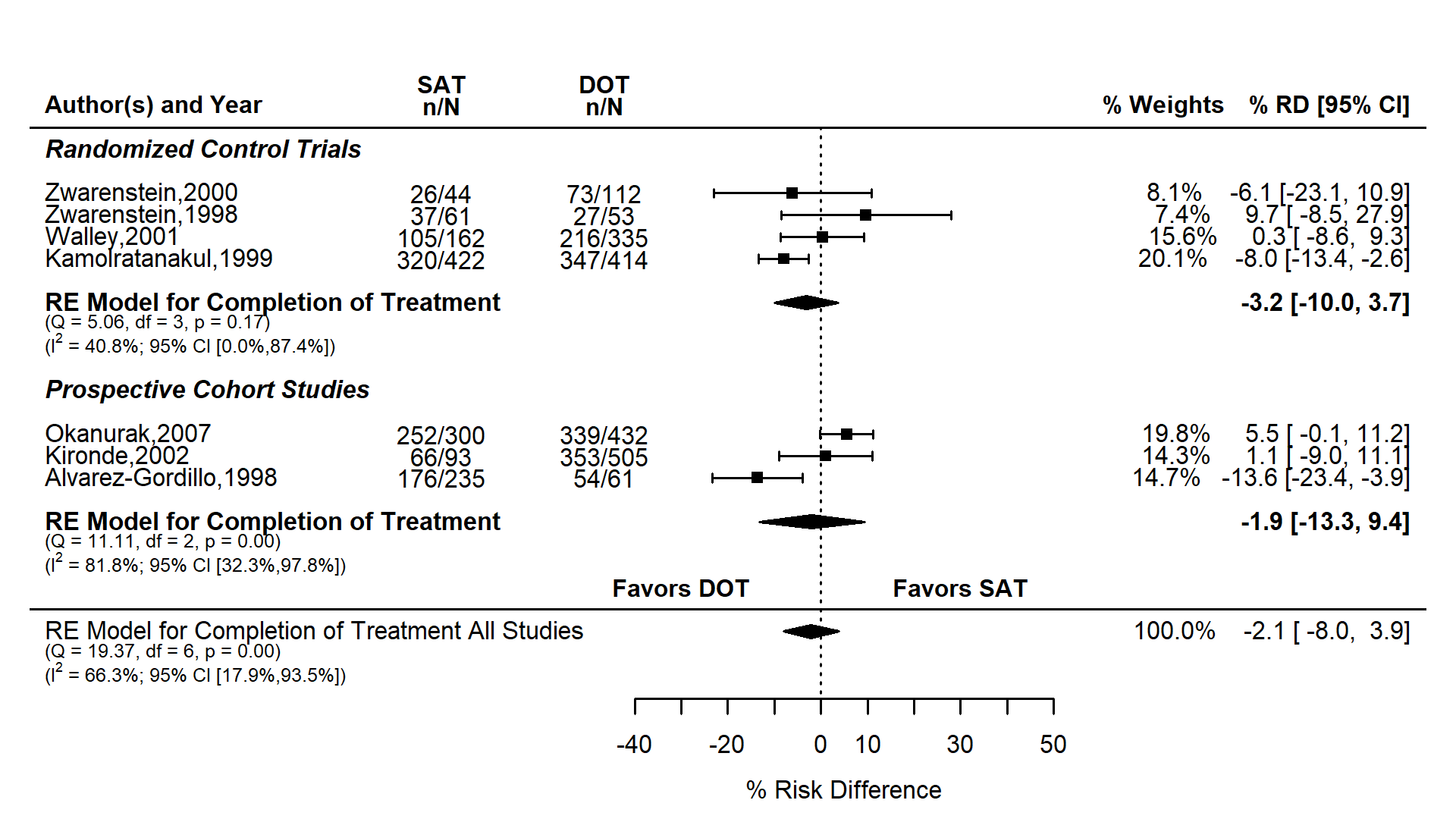

Supplement: S1 Folder — This folder contains all of the files described in the Supplementary Documentation. (ZIP) [file pone.0217219.s002.zip › Figures/Figure C.tif]

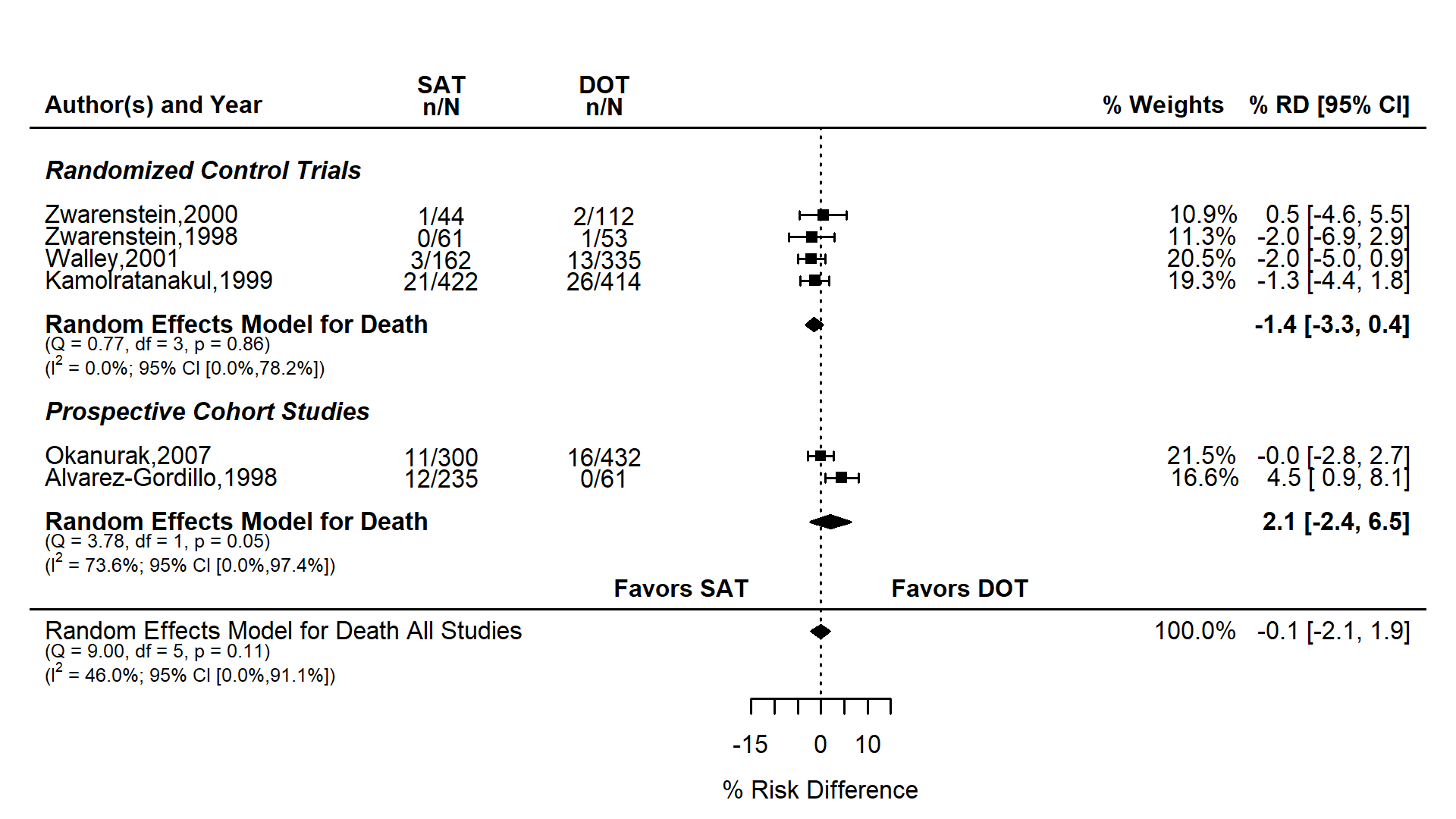

Supplement: S1 Folder — This folder contains all of the files described in the Supplementary Documentation. (ZIP) [file pone.0217219.s002.zip › Figures/Figure D.tif]

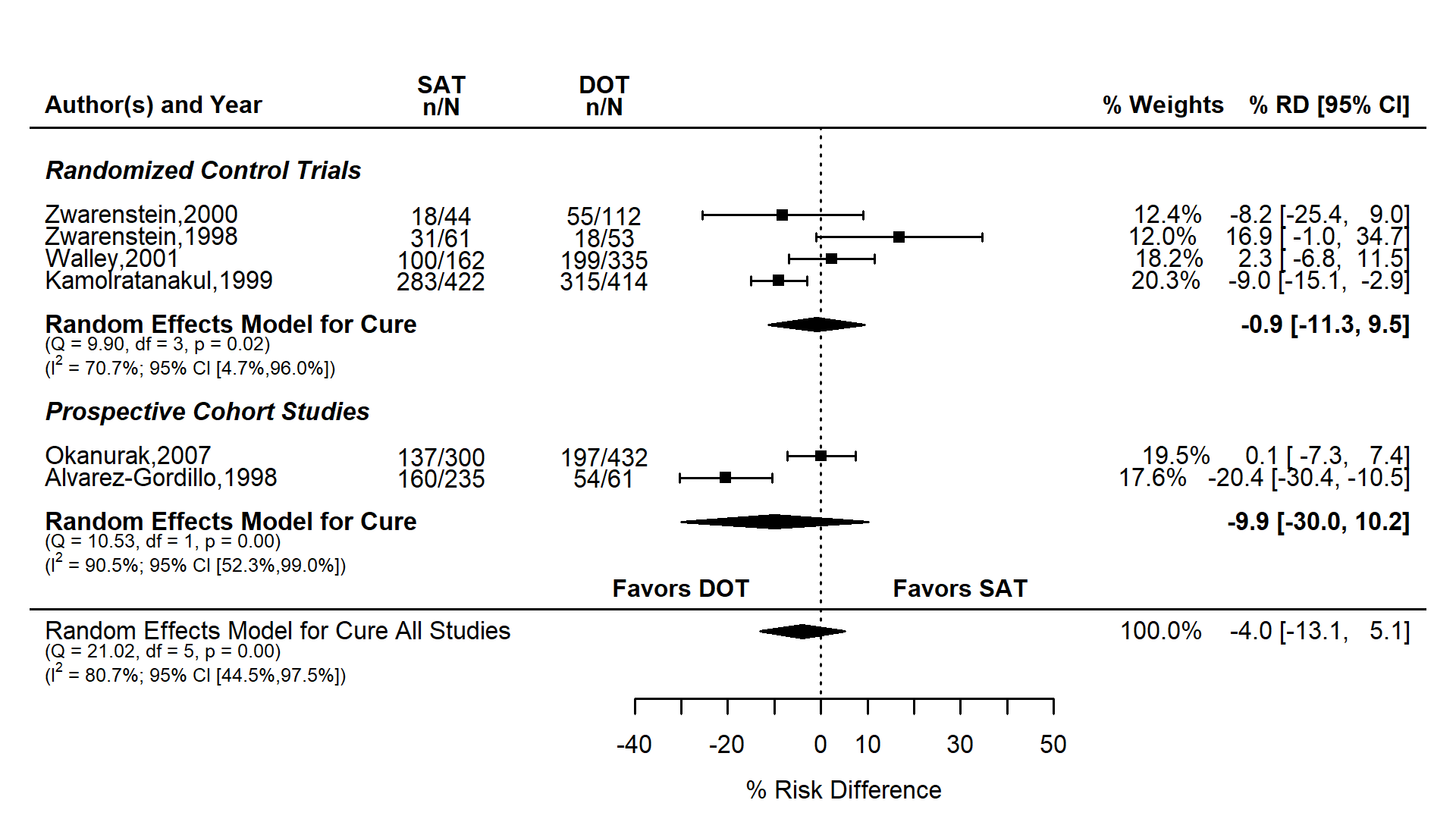

Supplement: S1 Folder — This folder contains all of the files described in the Supplementary Documentation. (ZIP) [file pone.0217219.s002.zip › Figures/Figure E.tif]

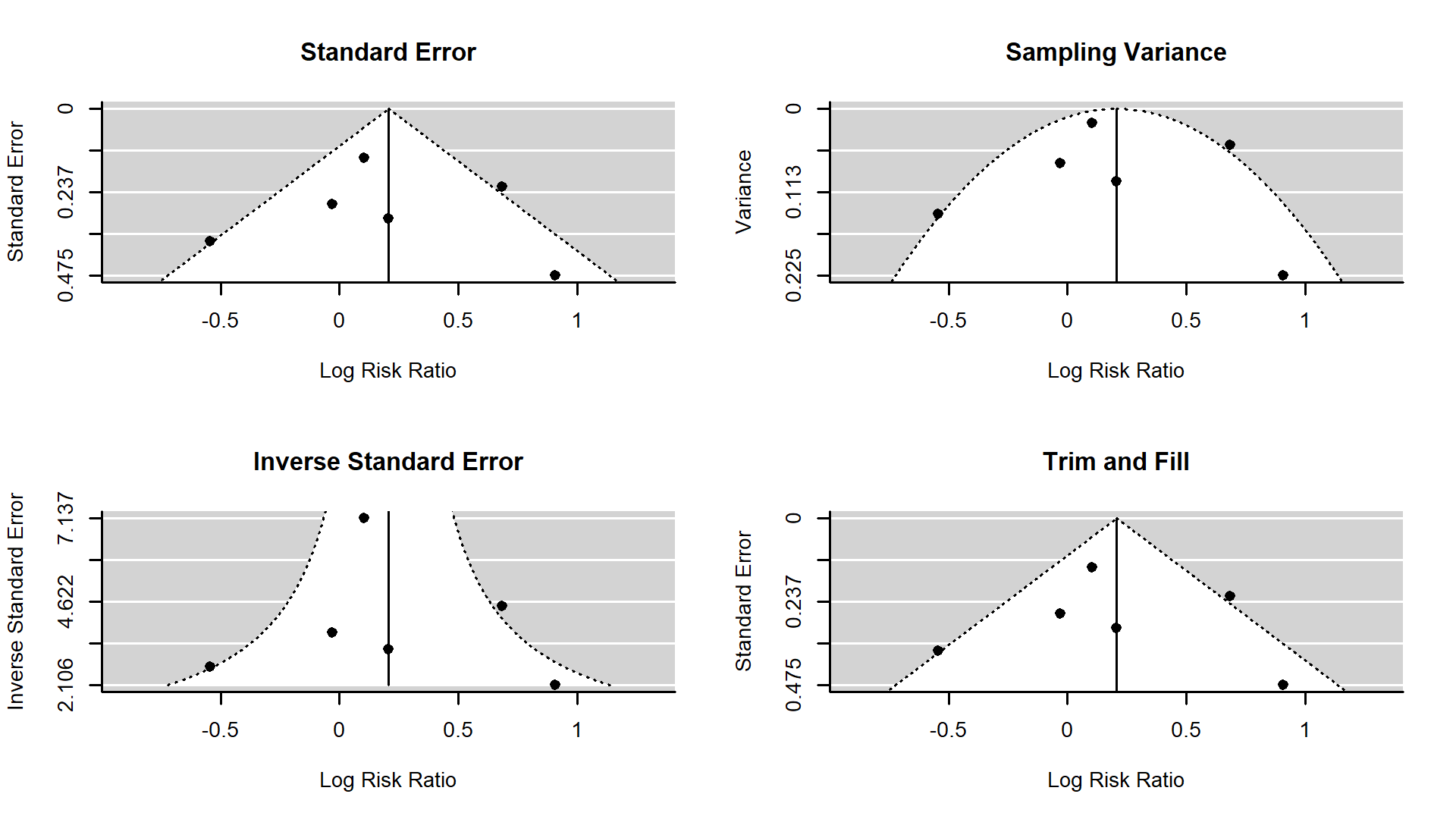

Supplement: S1 Folder — This folder contains all of the files described in the Supplementary Documentation. (ZIP) [file pone.0217219.s002.zip › Figures/Figure F.tif]

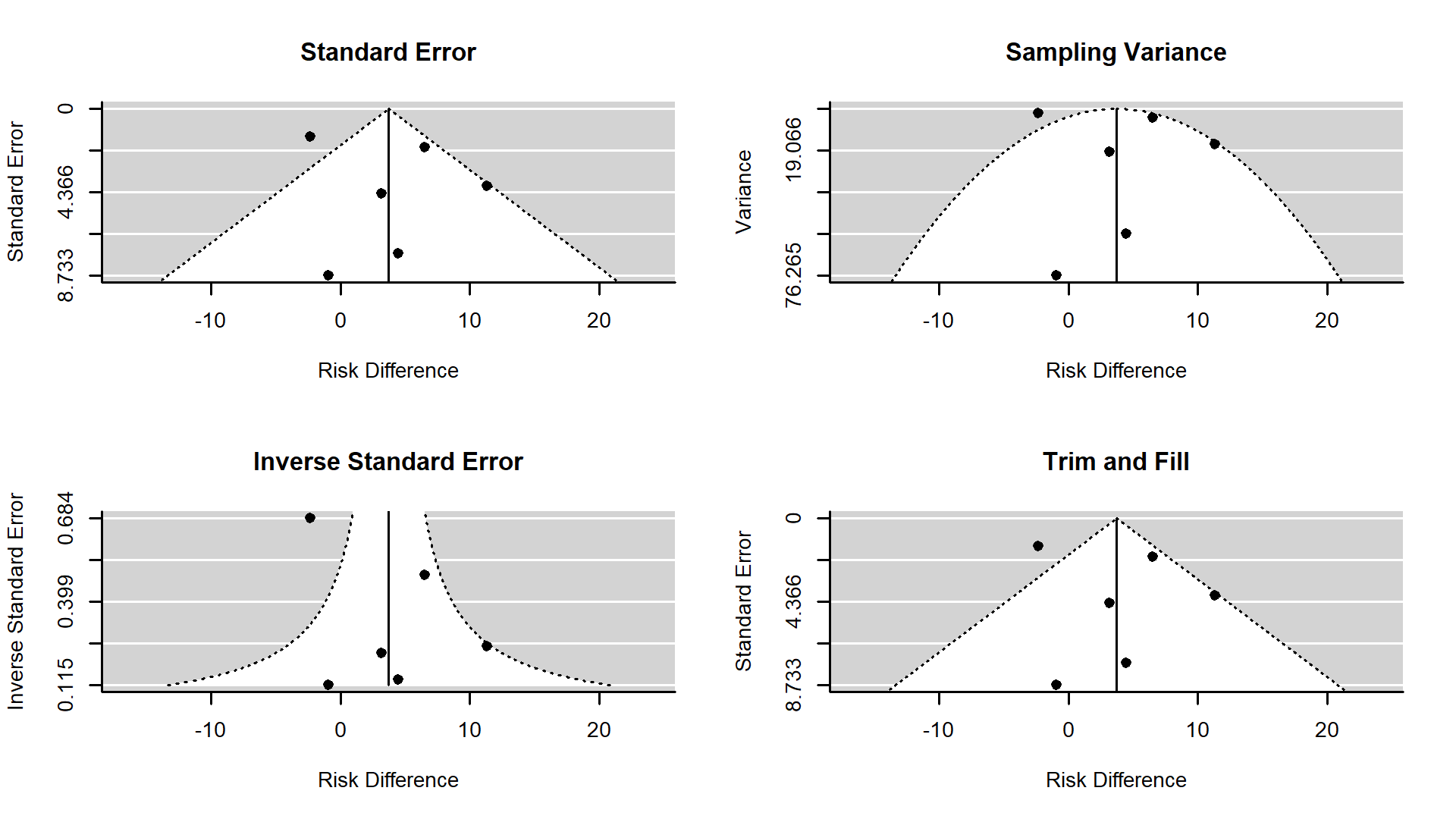

Supplement: S1 Folder — This folder contains all of the files described in the Supplementary Documentation. (ZIP) [file pone.0217219.s002.zip › Figures/Figure G.tif]

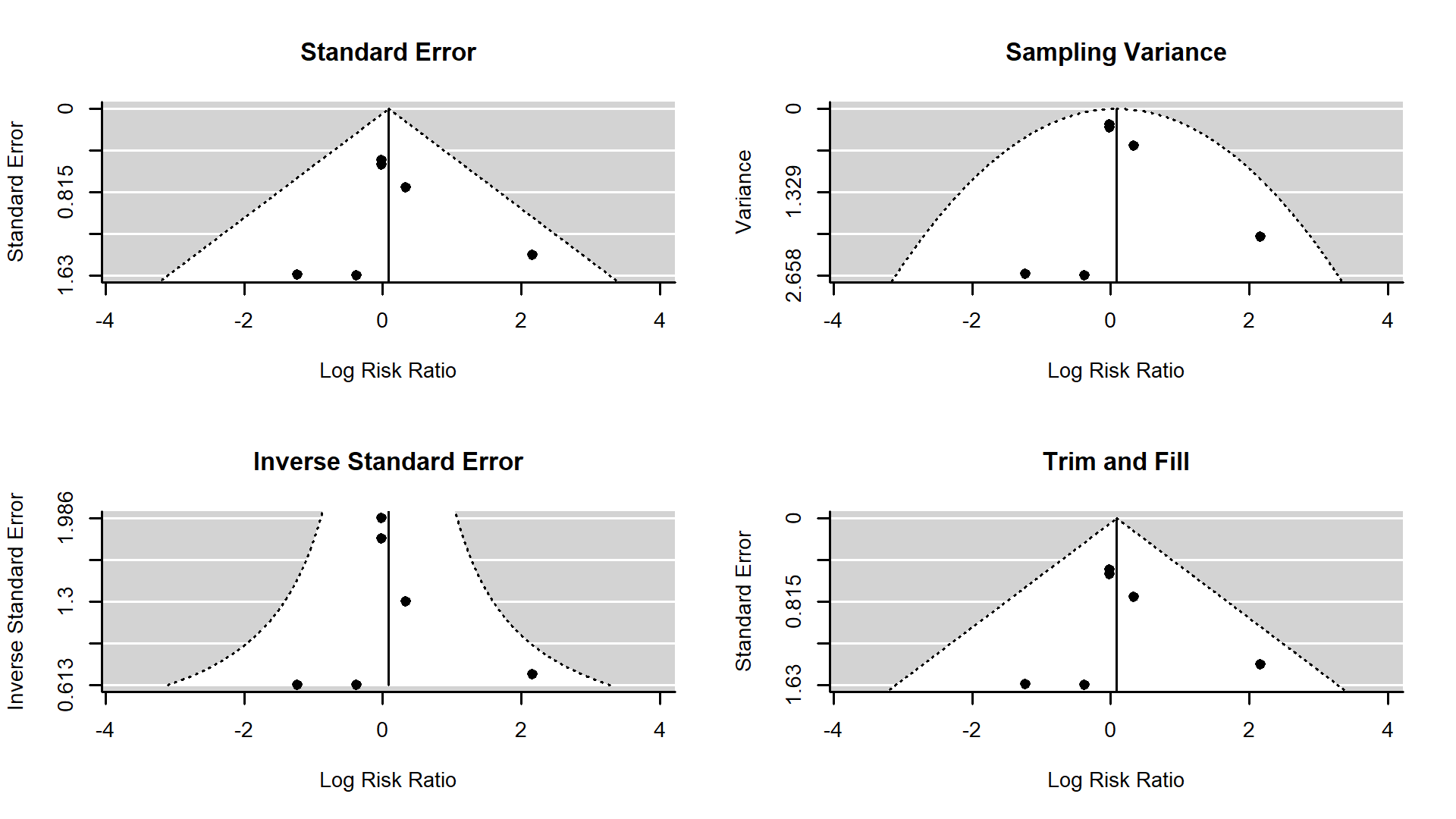

Supplement: S1 Folder — This folder contains all of the files described in the Supplementary Documentation. (ZIP) [file pone.0217219.s002.zip › Figures/Figure H.tif]

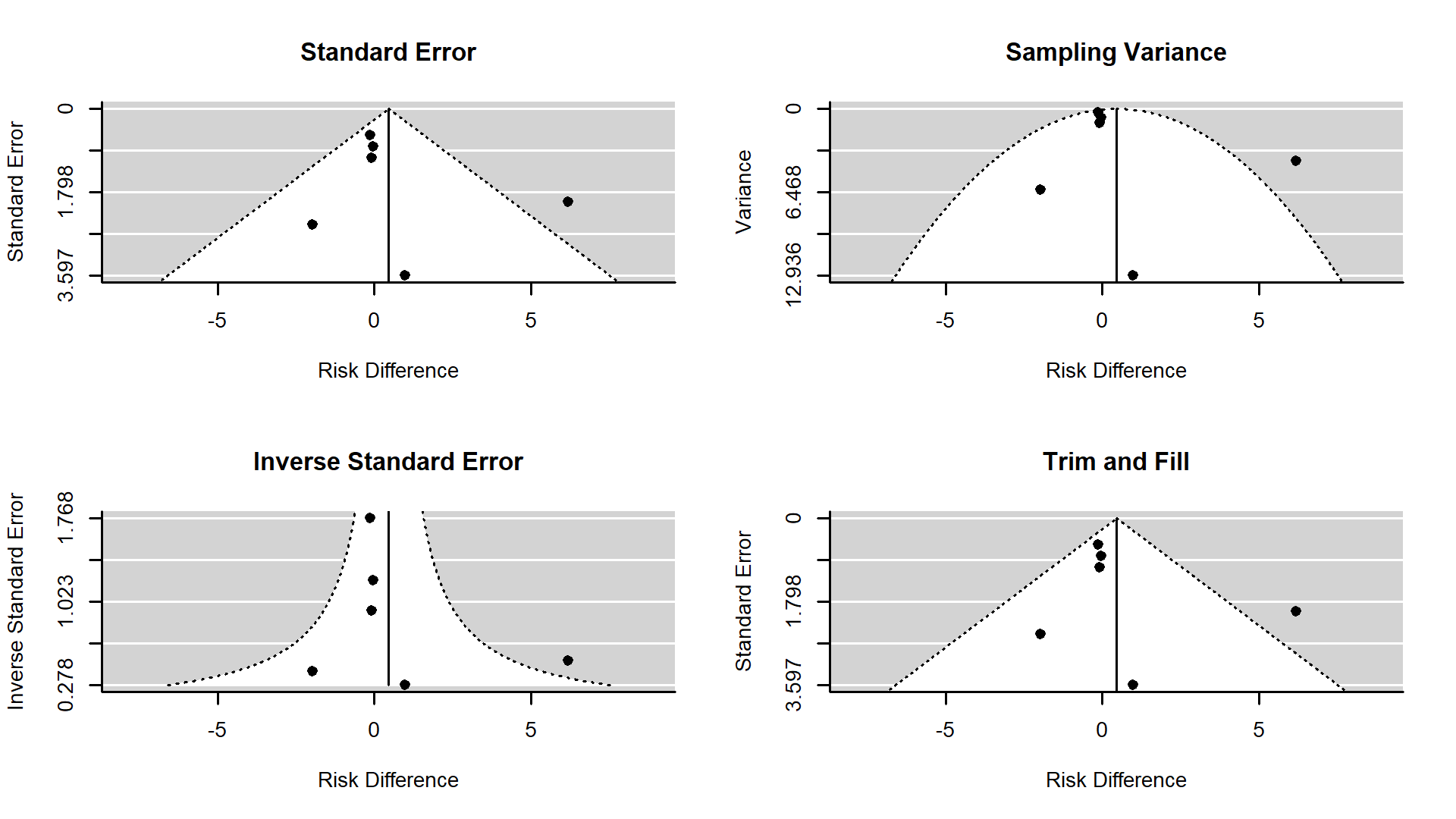

Supplement: S1 Folder — This folder contains all of the files described in the Supplementary Documentation. (ZIP) [file pone.0217219.s002.zip › Figures/Figure I.tif]

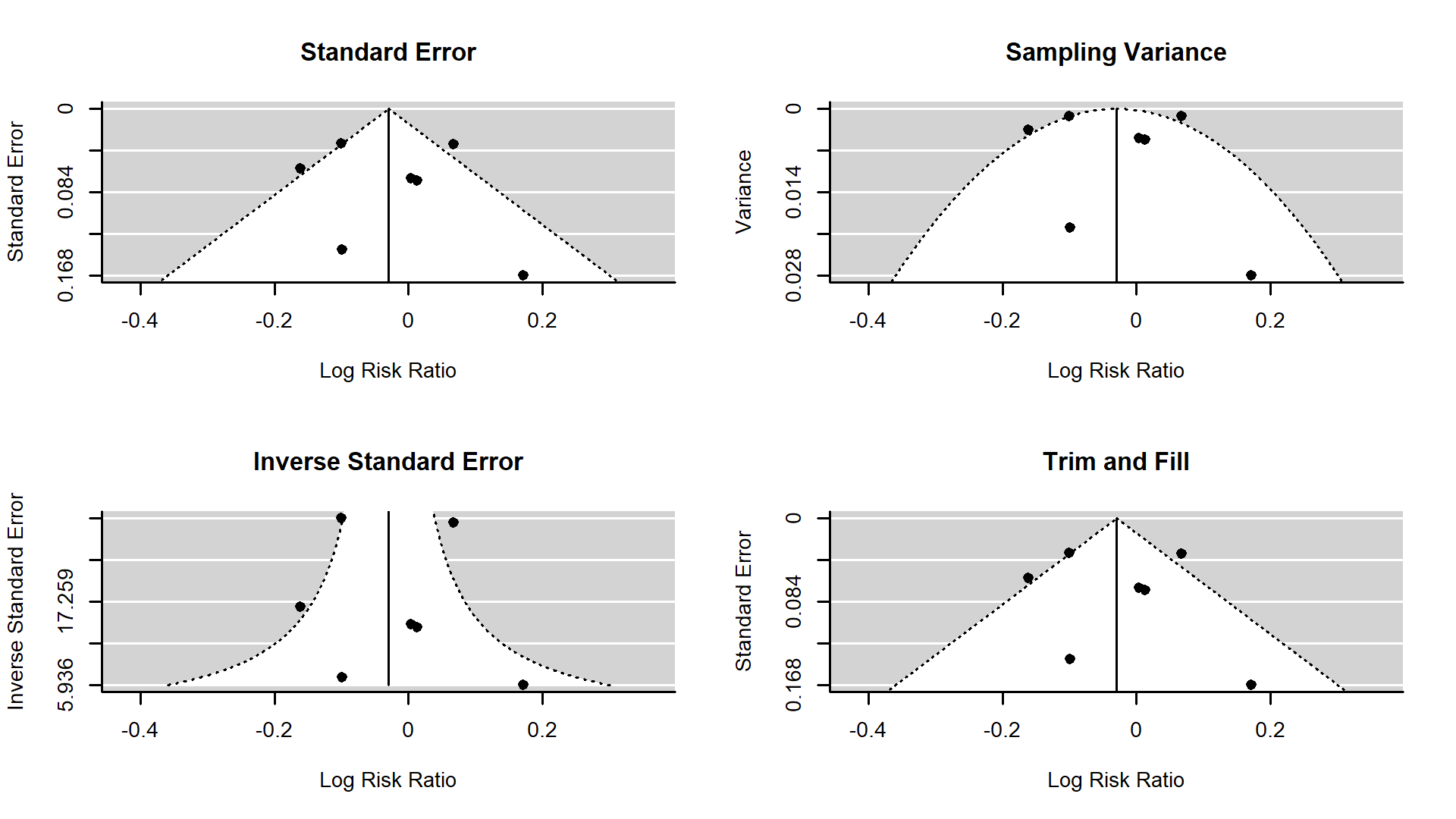

Supplement: S1 Folder — This folder contains all of the files described in the Supplementary Documentation. (ZIP) [file pone.0217219.s002.zip › Figures/Figure J.tif]

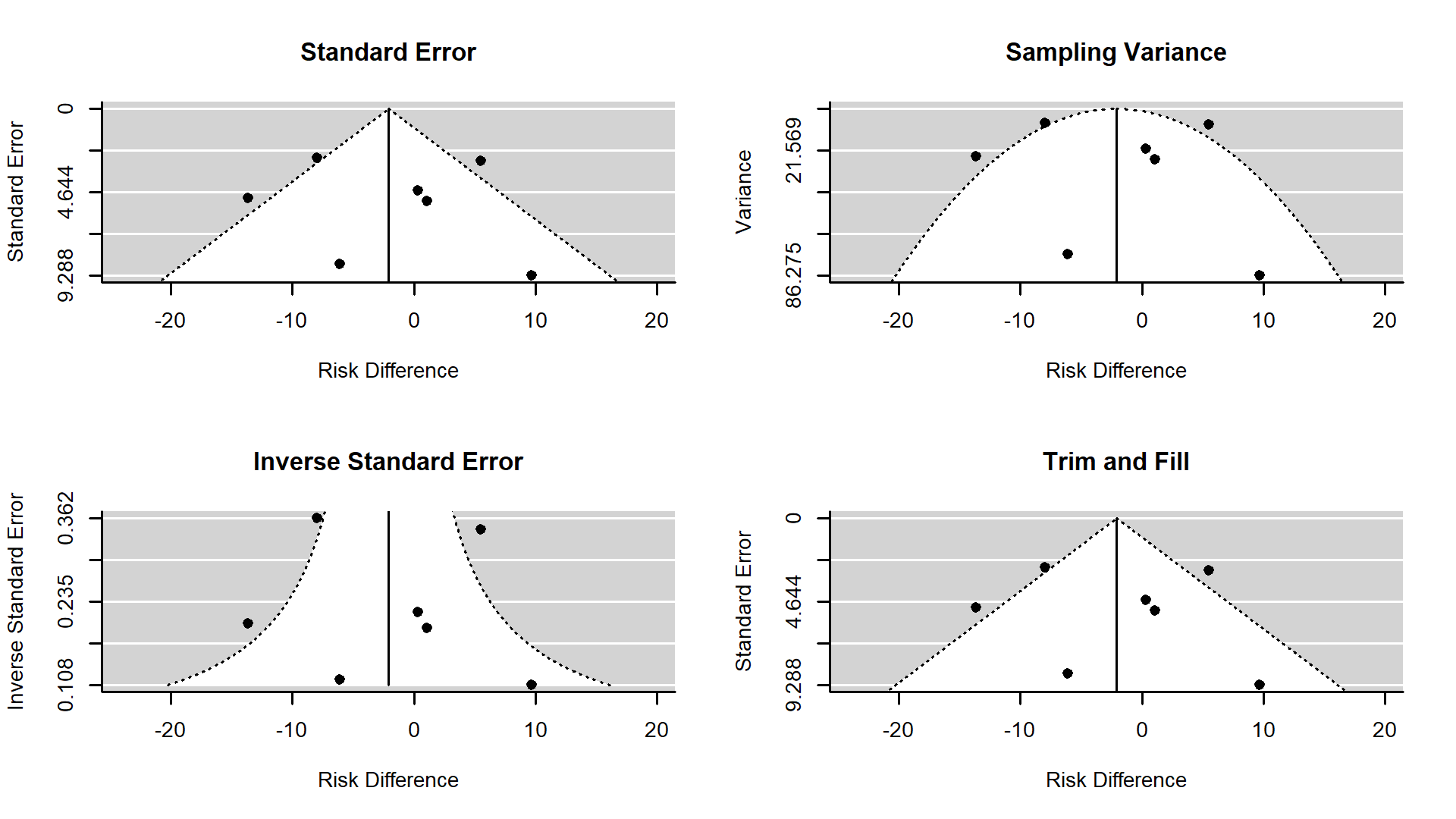

Supplement: S1 Folder — This folder contains all of the files described in the Supplementary Documentation. (ZIP) [file pone.0217219.s002.zip › Figures/Figure K.tif]

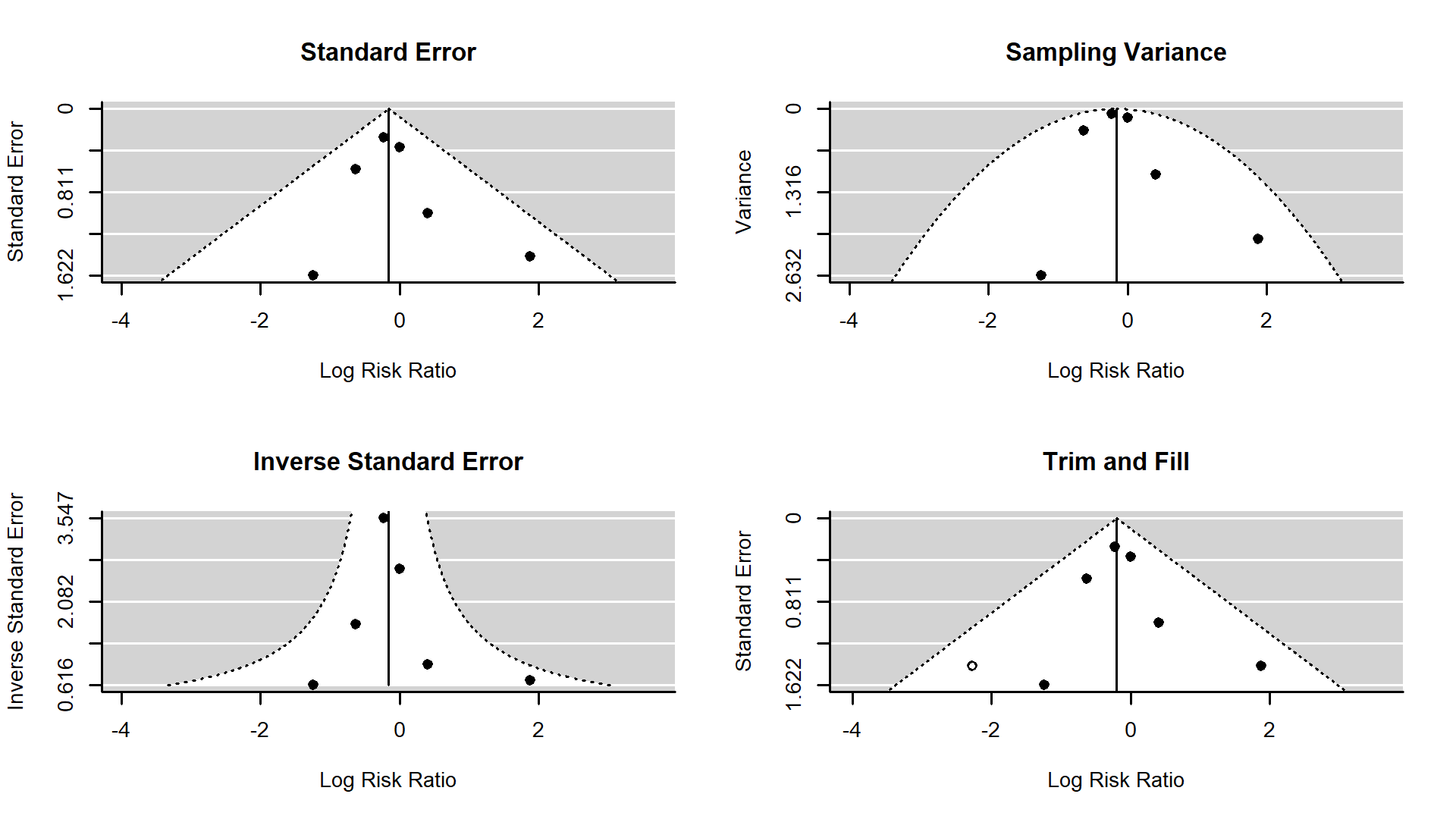

Supplement: S1 Folder — This folder contains all of the files described in the Supplementary Documentation. (ZIP) [file pone.0217219.s002.zip › Figures/Figure L.tif]

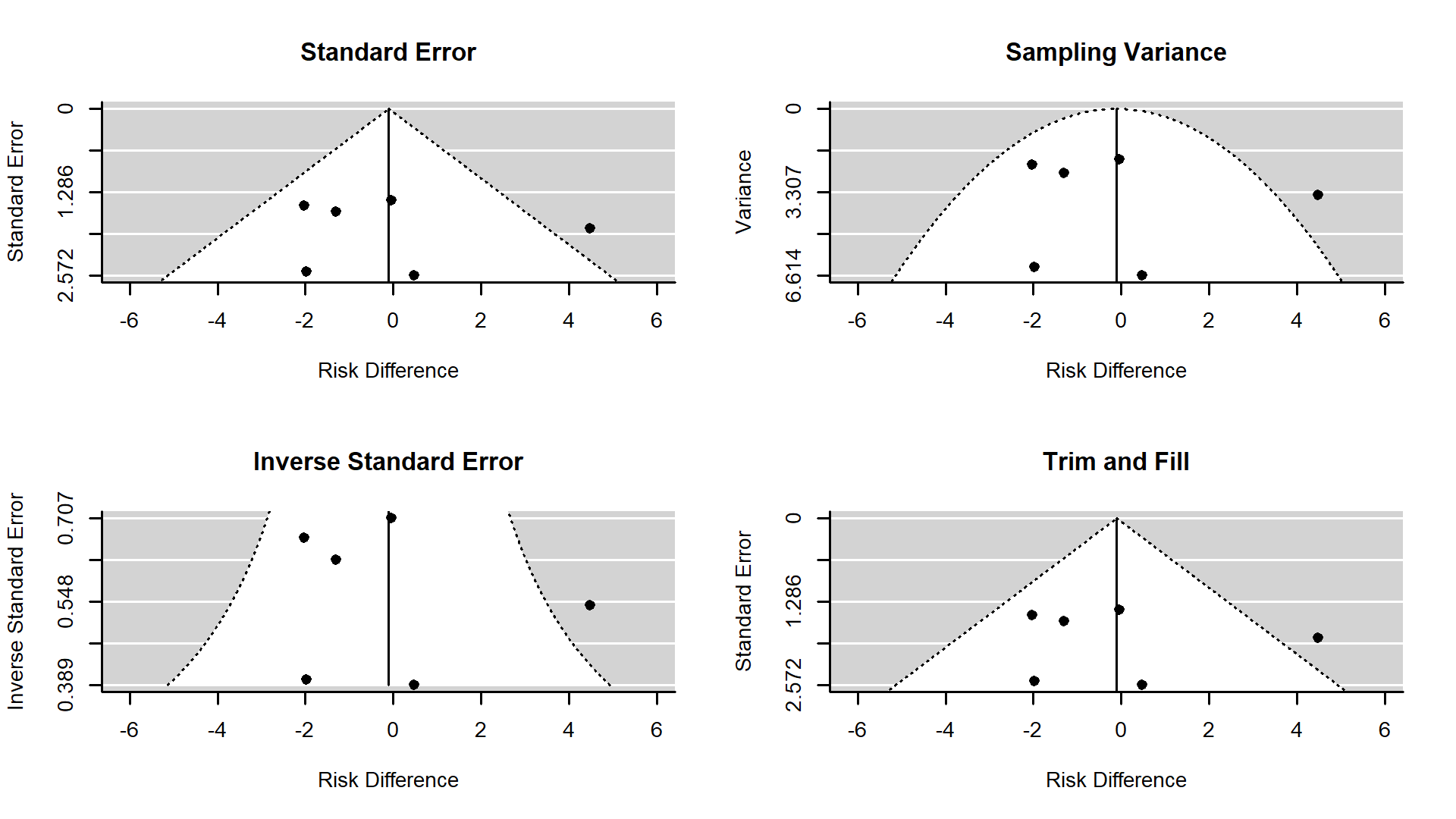

Supplement: S1 Folder — This folder contains all of the files described in the Supplementary Documentation. (ZIP) [file pone.0217219.s002.zip › Figures/Figure M.tif]

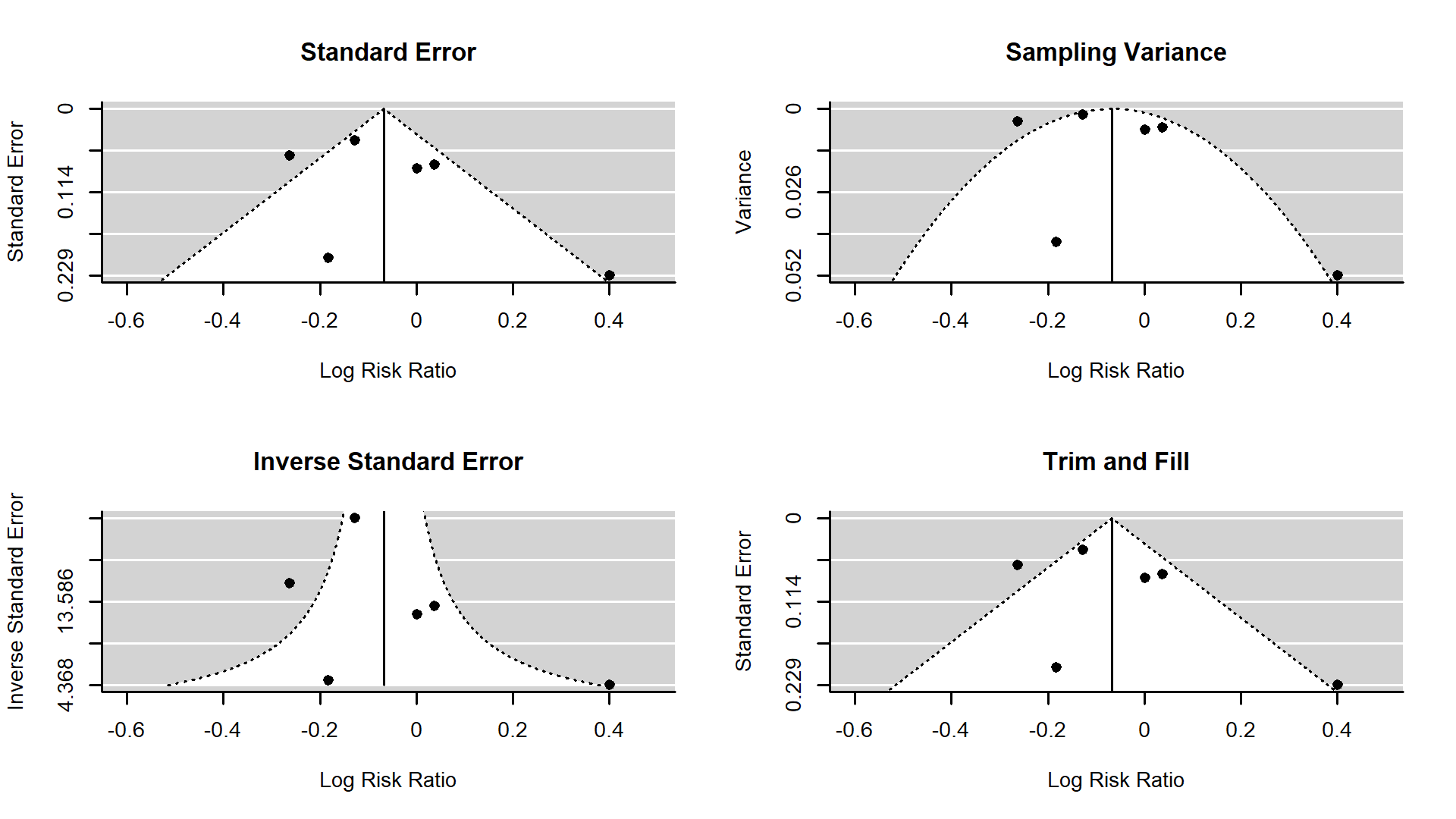

Supplement: S1 Folder — This folder contains all of the files described in the Supplementary Documentation. (ZIP) [file pone.0217219.s002.zip › Figures/Figure N.tif]

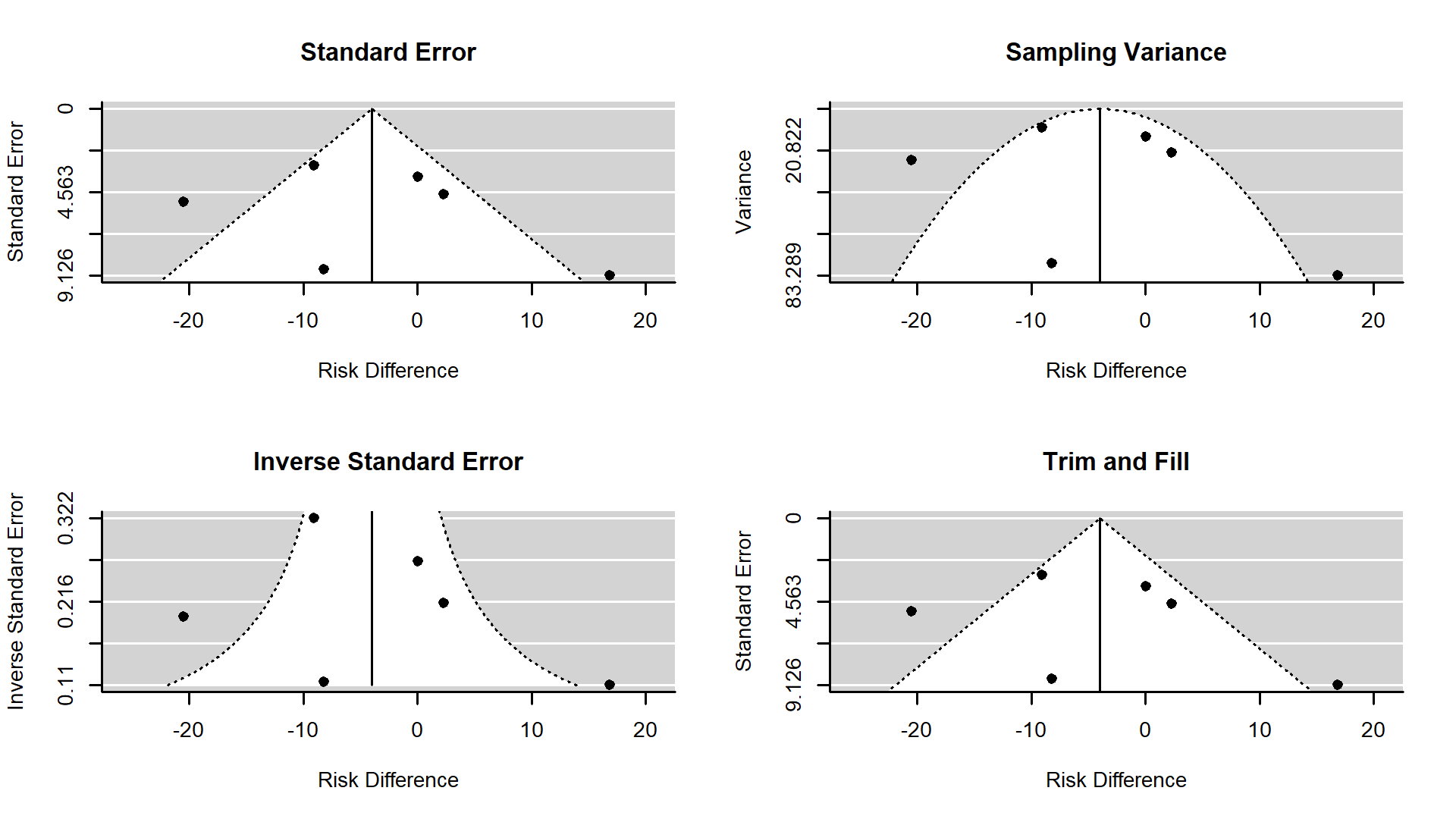

Supplement: S1 Folder — This folder contains all of the files described in the Supplementary Documentation. (ZIP) [file pone.0217219.s002.zip › Figures/Figure O.tif]

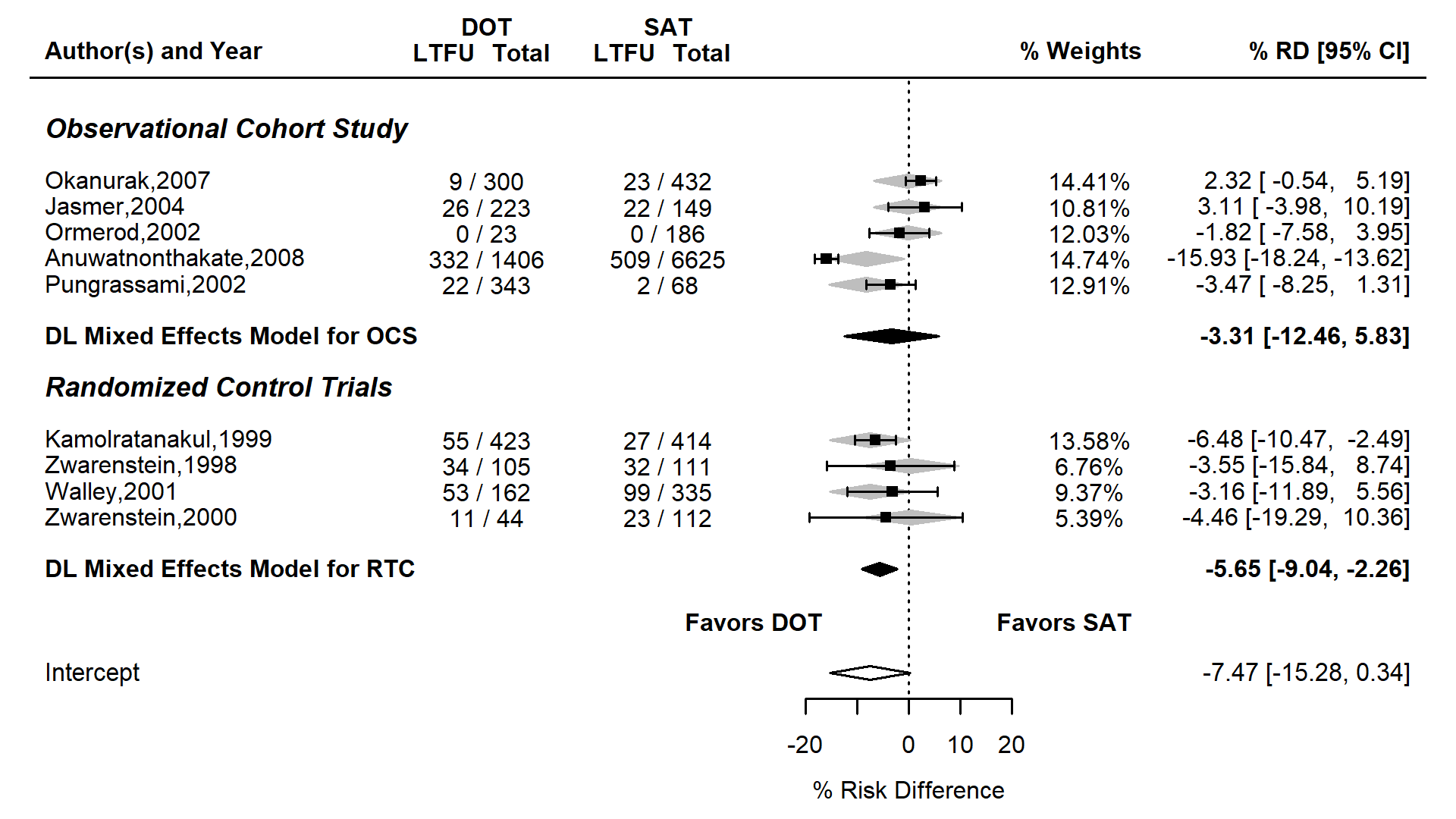

Supplement: S1 Folder — This folder contains all of the files described in the Supplementary Documentation. (ZIP) [file pone.0217219.s002.zip › Figures/Figure P.tif]

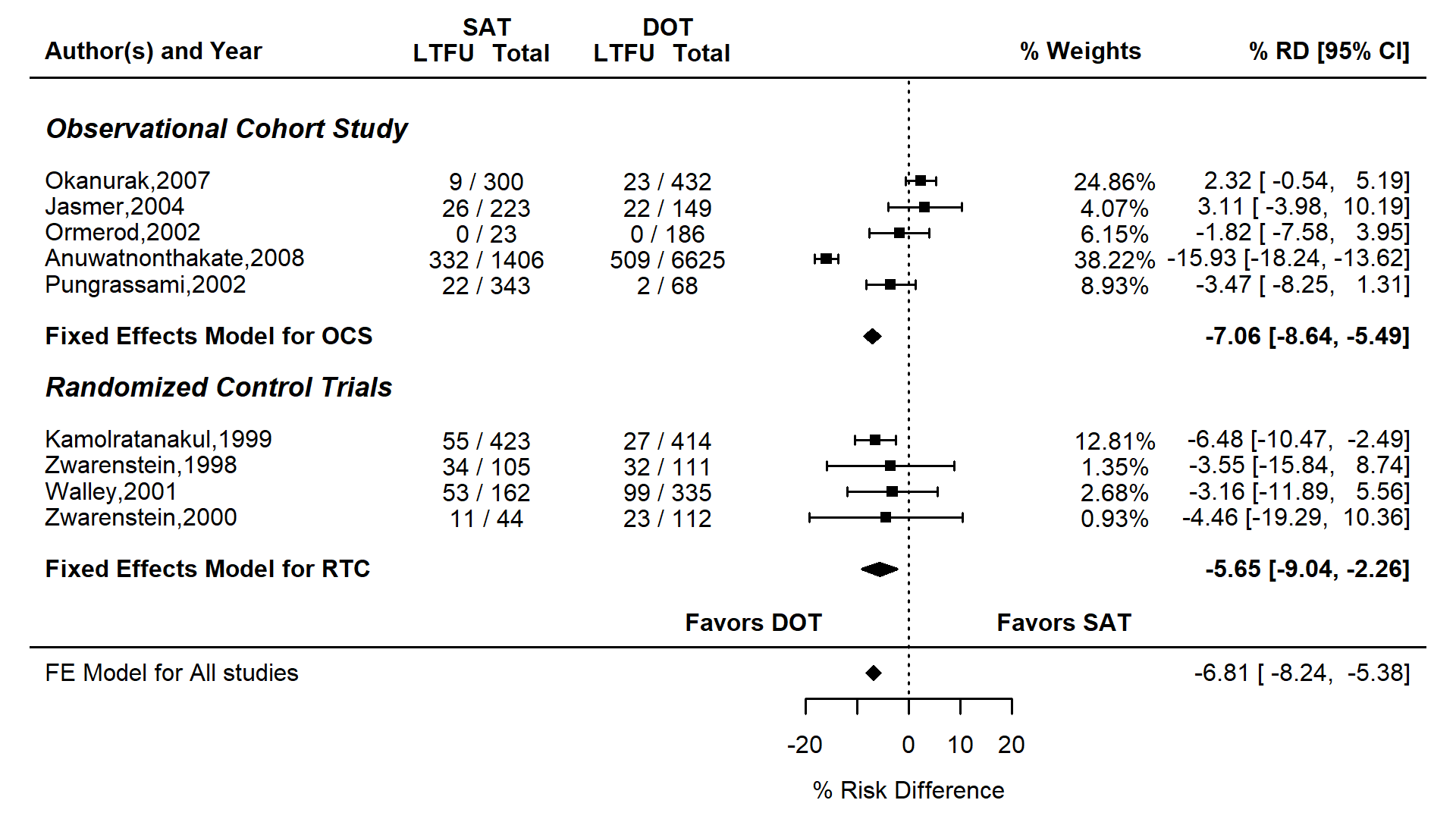

Supplement: S1 Folder — This folder contains all of the files described in the Supplementary Documentation. (ZIP) [file pone.0217219.s002.zip › Figures/Figure Q.tif]

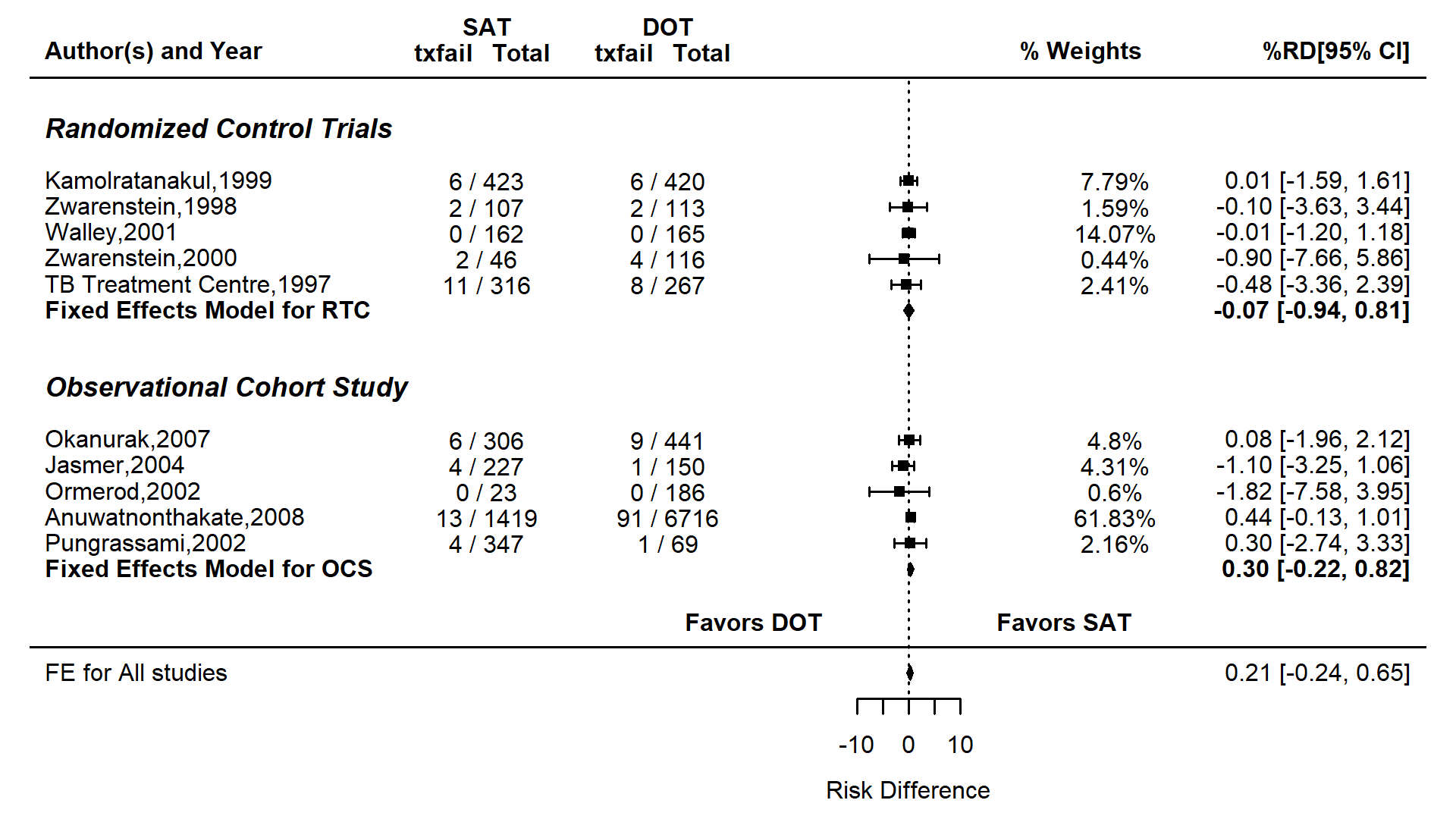

Supplement: S1 Folder — This folder contains all of the files described in the Supplementary Documentation. (ZIP) [file pone.0217219.s002.zip › Figures/Figure R.tif]
